# Covalent Template‐Directed Synthesis of a Spoked 18‐Porphyrin Nanoring**

**DOI:** 10.1002/anie.202302114

**Published:** 2023-03-24

**Authors:** Marcin A. Majewski, Wojciech Stawski, Jeff M. Van Raden, Michael Clarke, Jack Hart, James N. O'Shea, Alex Saywell, Harry L. Anderson

**Affiliations:** ^1^ Department of Chemistry University of Oxford Chemistry Research Laboratory Oxford OX1 3TA UK; ^2^ School of Physics & Astronomy University of Nottingham Nottingham NG7 2RD UK; ^3^ Current address: Faculty of Chemistry University of Wrocław ul. F. Joliot-Curie 14 50-383 Wrocław Poland

**Keywords:** Aromatic Compounds, Nanoring, Oxidative Coupling, Porphyrin, Template

## Abstract

Rings of porphyrins mimic natural light‐harvesting chlorophyll arrays and offer insights into electronic delocalization, providing a motivation for creating larger nanorings with closely spaced porphyrin units. Here, we demonstrate the first synthesis of a macrocycle consisting entirely of 5,15‐linked porphyrins. This porphyrin octadecamer was constructed using a covalent six‐armed template, made by cobalt‐catalyzed cyclotrimerization of an H‐shaped tolan with porphyrin trimer ends. The porphyrins around the circumference of the nanoring were linked together by intramolecular oxidative *meso‐meso* coupling and partial β‐β fusion, to give a nanoring consisting of six edge‐fused zinc(II) porphyrin dimer units and six un‐fused nickel(II) porphyrins. STM imaging on a gold surface confirms the size and shape of the spoked 18‐porphyrin nanoring (calculated diameter: 4.7 nm).

## Graphical abstract

Cobalt‐catalyzed alkyne trimerization followed by oxidating coupling and fusion of zinc porphyrin units was used to construct a porphyrin nanoring around a central covalent spoke. The size and shape of these nanorings (diameter: 4.7 nm) was confirmed by scanning tunnelling microscopy (STM). A combination of zinc(II) and nickel(II) porphyrin units was used to increase the efficiency of the synthesis and control the positions of oxidative fusion.

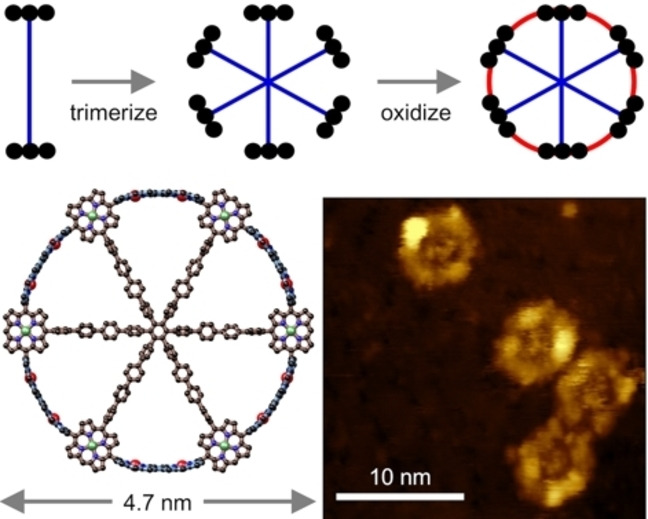

Cyclic arrays of chlorophyll molecules occur widely in photosynthetic light‐harvesting systems, such as those in purple bacteria.^[1]^ Synthetic rings of porphyrins are valuable models for understanding the photophysics of these light‐harvesting arrays.^[2]^ They are also studied as multitopic receptors for molecule recognition,^[3]^ and as expanded annulenes for exploring nanoscale aromaticity.^[4]^ Many large porphyrin‐based macrocycles have been synthesized using oligopyridine ligands as non‐covalent templates.^[5]^ Here we investigate the use of a covalent template for the synthesis of an 18‐porphyrin nanoring. The main advantage of a covalently attached template is that it allows coupling reactions to be carried out under conditions that would cause a non‐covalent template to dissociate. Previously, covalent templates have been used to synthesize a wide variety of macrocycles,^[6]^ including some spectacular giant spoked wheels,[^7^, ^8^, ^9^] but to the best of our knowledge, they have not been used to prepare cyclic porphyrin oligomers.

This work is part of a project directed towards the investigation of 5,15‐linked porphyrin nanorings **5,15‐P*N*** and edge‐fused porphyrin nanobelts **EF‐P*N*** (Figure 1). There are no previous reports of macrocycles of the type **5,15‐P*N***, although 5,10‐linked cyclic porphyrin oligomers have been synthesized.^[10]^ Recently, we achieved the synthesis of a 5,15‐linked 24‐porphyrin nanoring with a single butadiyne bridge, using non‐covalent oligopyridine templates.^[11]^ However, it has not yet been possible to synthesize fully 5,15‐linked porphyrin nanorings using that strategy, which is why we turned to covalent templates.

**Figure 1:**
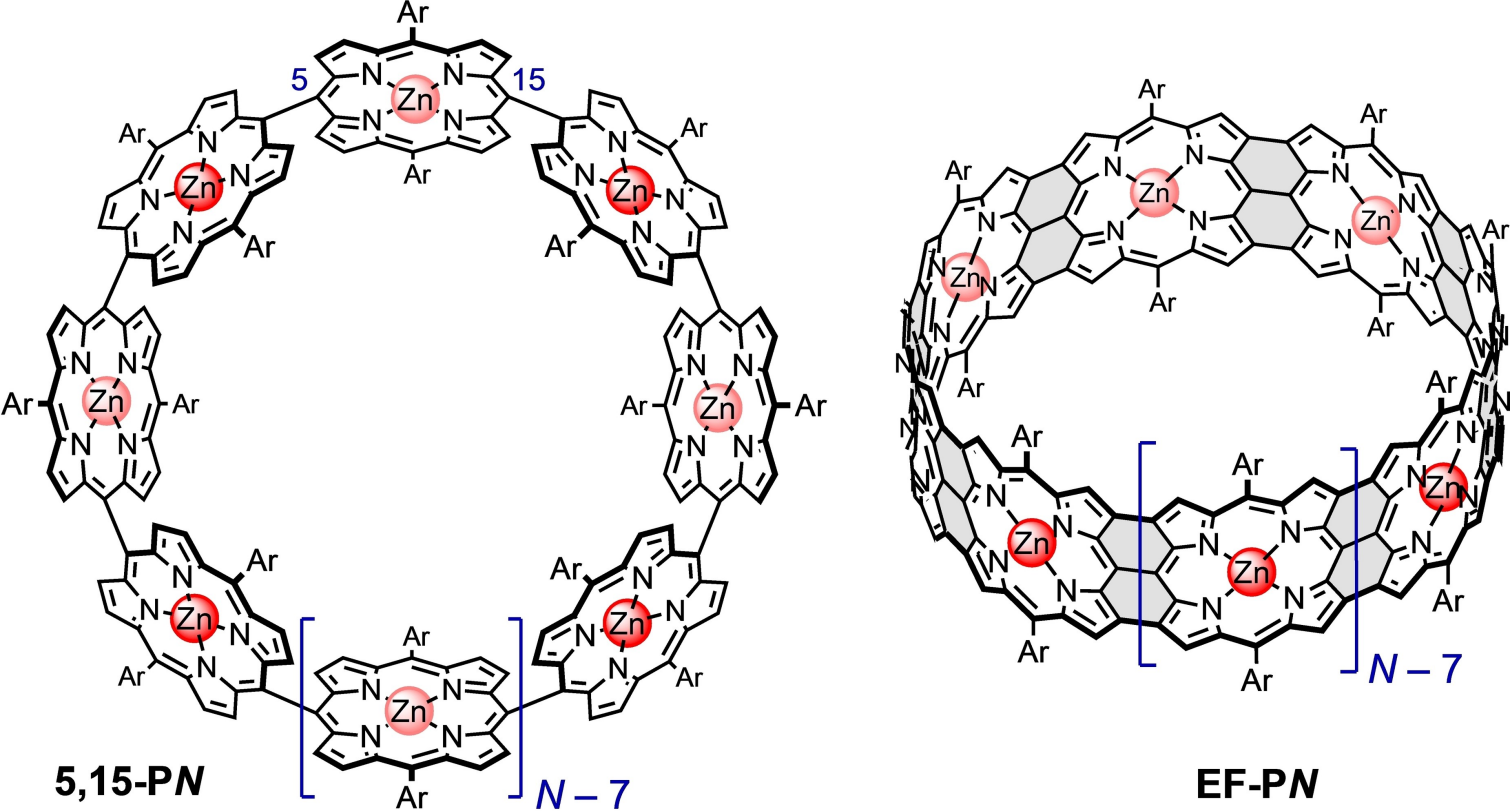
Generalized structures of a fully 5,15‐linked porphyrin nanoring **5**,**15‐P*N*** and an edge‐fused porphyrin nanobelt **EF‐P*N***. (*N* is the number of porphyrin units in the ring and Ar is an aryl solubilizing group.)

Osuka and co‐workers pioneered the synthesis of linear 5,15‐linked porphyrin oligomers, consisting of up to 1024 porphyrin units.^[12]^ These oligomers are not π‐conjugated, due to the severe twist between neighboring porphyrins, but there is strong exciton coupling along the chain, leading to ultra‐fast excited‐state energy migration,^[13]^ which makes it interesting to investigate the photophysics of **5,15‐P*N*** s. Fully 5,15‐linked porphyrin nanorings are also potential precursors to edge‐fused porphyrin nanobelts **EF‐P*N***. Molecular belts of this type have not yet been synthesized, although they were mentioned in a theoretical study.^[14]^ They are fascinating targets because the corresponding linear porphyrin tapes display exceptional electronic delocalization, leading to optical electronic transitions in the IR^[15]^ and single‐molecule conductance that is almost independent of length.^[16]^

We used a six‐armed covalent oligophenylene template to direct the formation of the 5,15‐linked 18‐porphyrin macrocycle **1**, by oxidative coupling of the non‐cyclic hexamer of trimers **2** (Scheme 1). These compounds have a mixture of zinc(II) and nickel(II) metalation to facilitate the synthesis: preliminary studies showed that *meso*‐bromo nickel(II) porphyrins undergo more efficient Suzuki coupling, whereas oxidative *meso‐meso* coupling occurs most easily with zinc(II) porphyrins.^[17]^ The most innovative step in this synthesis is perhaps the construction of **2** by trimerization of the H‐shaped porphyrin hexamer **3**. This trimerization strategy has the advantage that it generates **2** without forming closely related species that would be difficult to separate. Use of cobalt‐catalyzed alkyne trimerization in the synthesis of **1** was inspired by the syntheses of spoked wheel structures reported by Müllen^[7]^ and Höger.^[8]^

**Scheme 1:**
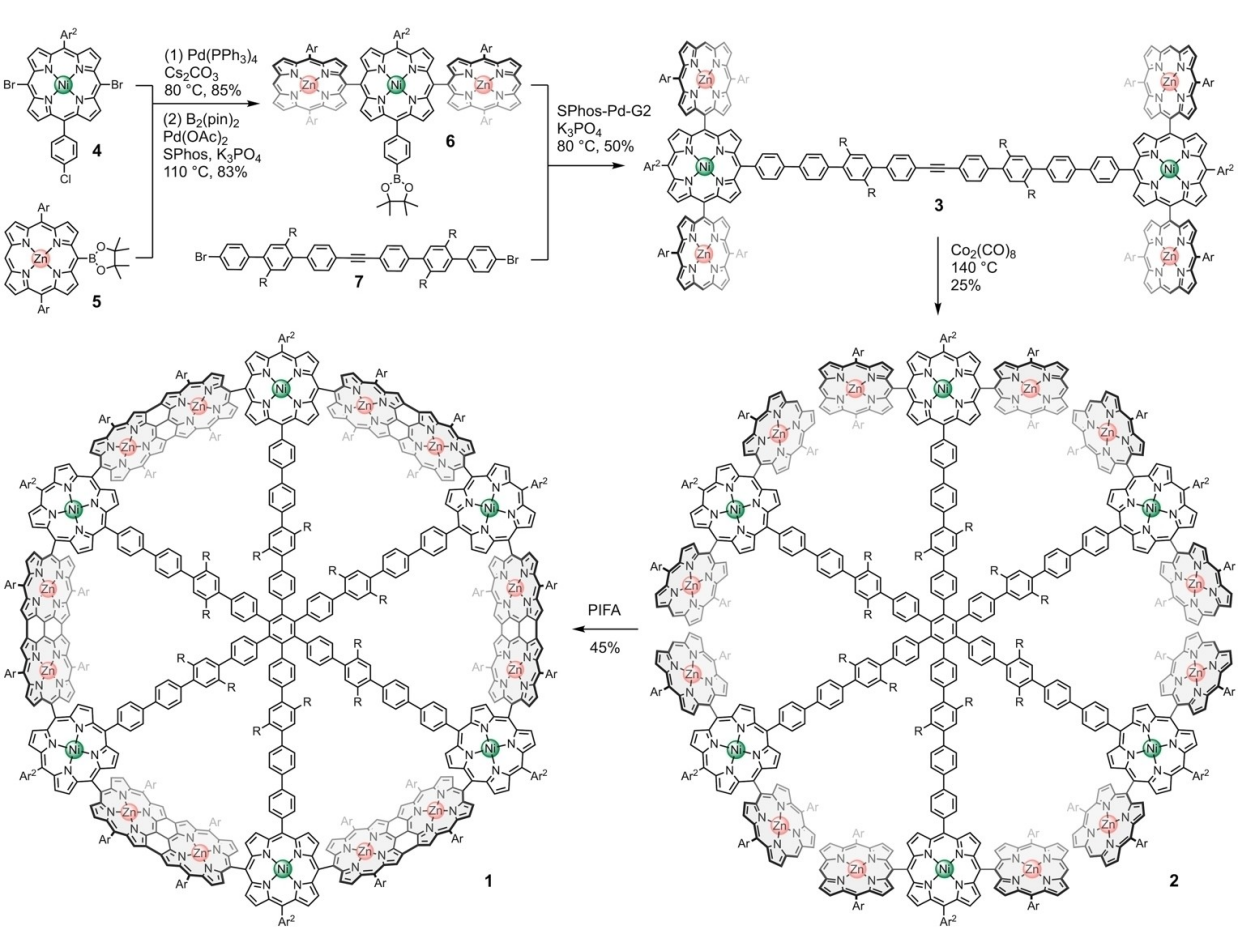
Synthesis of the partially fused, fully 5,15‐linked, porphyrin nanoring **1**. Ar=3,5‐bis(octyloxy)phenyl, Ar^2^=3,5‐bis(tri‐*n*‐hexylsilyl)phenyl, R=*n‐*C_8_H_17_. See Supporting Information for details of solvents, concentrations and reaction times.

Suzuki–Miyaura coupling of dibromo nickel(II) porphyrin **4** with two equivalents of zinc porphyrin boronic ester **5**, followed by Miyaura borylation of the *para‐*chlorophenyl substituent, gave porphyrin trimer **6** (70 % yield over two steps). Coupling of **6** with dibromide **7** generated porphyrin hexamer **3** in 50 % yield. Cobalt‐catalyzed trimerization of tolan **3** gave the porphyrin 18‐mer **2**, which was isolated in 25 % yield after purification by recycling gel‐permeation chromatography (GPC). Previously, this type of trimerization reaction has been used to synthesize radial porphyrin hexamers^[18]^ and phenylene‐based spoked wheels,[^7^, ^8^] but it has not been applied to create molecules in this size regime (molecular weight of **2**: 21.7 kDa). Our optimized reaction conditions for conversion of **3** to **2** require the use of one equivalent of Co_2_(CO)_8_ and a high concentration of **3** in toluene (35 mM) in a sealed tube at 140 °C for 16 hours. The high solubility of **3** and **2** in toluene, conferred by the octyloxy, tri‐*n*‐hexylsilyl and octyl sidechains, is crucial for the success of this reaction. The ^1^H NMR spectra of **3** and **2** are similar (Figure 2), indicating unhindered fast rotation of individual subunits on the NMR timescale. ^13^C NMR spectroscopy clearly shows disappearance of the alkyne signal at δ_C_=89.8 ppm on conversion of **3** to **2** (Figures S27, S35), while MALDI TOF mass spectrometry shows the expected three‐fold increase in molecular weight (Figures S28, S36).

**Figure 2:**
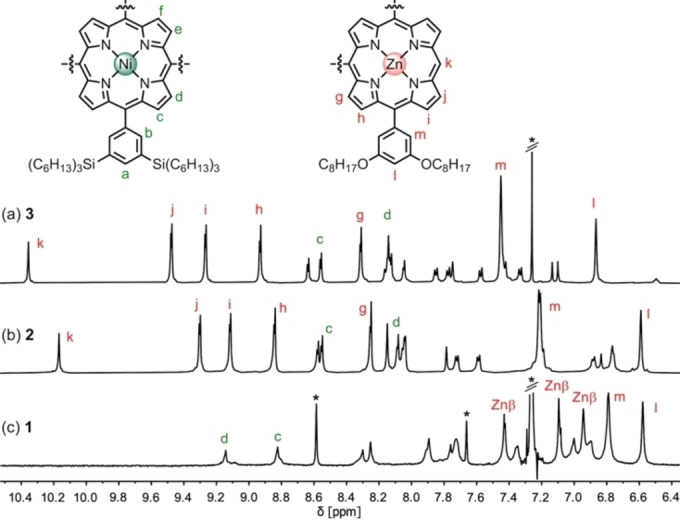
Aromatic region of ^1^H NMR spectra of: a) **3** (CDCl_3_), b) **2** (CD_2_Cl_2_) and: c) **1** (CDCl_3_+1 % *d_5_*‐pyridine; Znβ indicates (g,h,i); 600 MHz, 298 K; * indicates residual solvent peaks in *d_5_*‐pyridine or CDCl_3_, see Supporting Information for full assignments.

*Meso*‐*meso* coupling and fusion of all the zinc(II) porphyrin units in **2** was carried out to give nanoring **1**, without isolating the intermediate *meso*‐*meso*‐linked ring, using bis(trifluoroacetoxy)iodobenzene (PIFA) as the oxidant.[^17^, ^19^] The analytical GPC trace of the turquoise product from cyclization of **2** with PIFA showed a main fraction with the same retention time as **2**, indicating a similar molecular size. However, the electronic properties manifested in the UV/Vis‐NIR absorption spectra are completely different (Figure 3). The most red‐shifted absorption band of **1** is located at 1055 nm, in a spectral window characteristic of edge‐fused Zn^II^‐Zn^II^ porphyrin dimers.^[15]^ In comparison, absorption bands of both tolan **3** and octadecamer **2** do not extend beyond 600 nm. Moreover, the absorption spectra match well with those of previously reported linear mixed‐metal Ni^II^ monomer‐Zn^II^ fused dimer hybrids (see Figure S1,2).^[17]^

**Figure 3:**
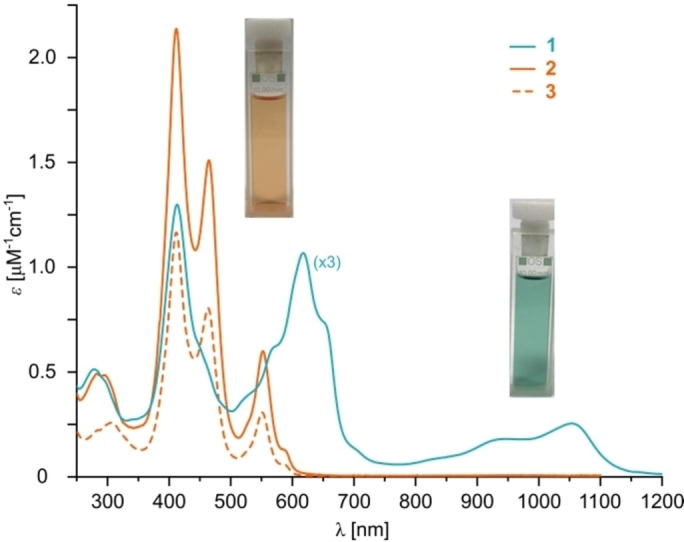
UV/Vis‐NIR absorption spectra of **1**, **2** and **3**. Insets: photographs of cuvettes of **1** (turquoise) and **2** (tawny). All samples in CH_2_Cl_2_, 25 °C.

The identity of nanoring **1** was confirmed by NMR spectroscopy (Figure 2c) and mass spectrometry. The ^1^H NMR spectrum shows the absence of a *meso* proton (*k*, δ_H_≈10.2 ppm), while retaining the symmetry of **2**. Most of the resonances in the ^1^H NMR spectrum of **1** were assigned using 2D COSY and NOESY spectra (Figures S48–S51). Insights into the 3D shapes of **1** and **2** were provided by scanning tunnelling microscopy (STM) characterization of molecules deposited on an Au(111) surface, held under vacuum conditions, by electrospray deposition from solutions in toluene/methanol (Figure 4a–c). Images for **1** are approximately circular: circumference 12±1 nm; long axis 4.1±0.3 nm; short axis 3.7±0.3 nm. These dimensions compared well with Ni⋅⋅⋅Ni diameter of 4.7 nm from semiempirical tight binding *xTB* calculations (Figure 4d). The central template is visible inside the ring in the STM images (Figure 4a). Images of **2** are similar to those of **1**, but the molecules are slightly larger and more elliptical (circumference 15±1 nm; long axis 5.5±0.3 nm; short axis 4.3±0.2 nm), and the template component is less clearly visible, which probably reflects the greater flexibility of **2** (Figure 4c). XPS spectra of samples of **1** and **2** on a gold surface confirmed the presence of zinc and nickel, and show a single nitrogen chemical environment, in agreement with the presence of metalated porphyrin species. Elemental composition ratios for Zn : Ni and N : Ni (2 : 1 and 6 : 1, respectively—Figure S58) agree with the expected stoichiometries of **1** and **2**.

**Figure 4:**
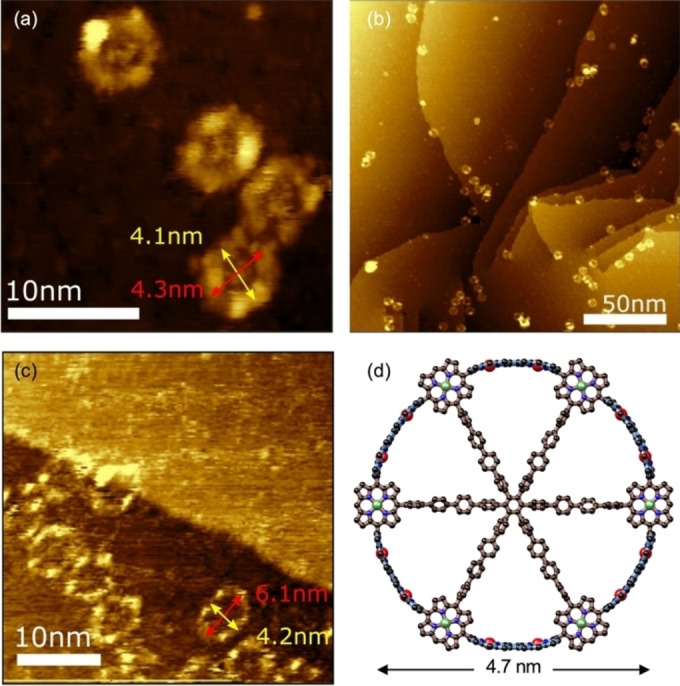
STM images of (a) and (b) **1** and c) **2** deposited onto a Au(111) surface. Image parameters: (a) and (c) sample bias −2.0 V, set‐point current 15 pA; b) sample bias −1.0 V, set‐point current 20 pA. d) Projection of the calculated geometry of **1** from tight binding calculations using the *xTB* software (version 6.4.1) with *GFN1* parameters; see Supporting Information for details. (Alkyl and aryl solubilizing groups were replaced by hydrogen atoms to simplify the calculations; H atoms are not shown in the graphic.)

In conclusion, we have demonstrated an efficient synthesis of the first example of a partially fused, directly 5,15*‐*linked porphyrin nanoring, without any linkers between the adjacent porphyrin units. The new spoked nanoring was characterized both in solution and on surface by STM. This work prepares the way for the synthesis of similar covalently templated **5,15‐P*N*** nanorings using a template with cleavable links (e.g. esters), so that the template can be removed and the whole belt can be fused to give an edge‐fused nanobelt **EF‐P*N***.

## Conflict of interest

The authors declare no conflict of interest.

## Supporting information

As a service to our authors and readers, this journal provides supporting information supplied by the authors. Such materials are peer reviewed and may be re‐organized for online delivery, but are not copy‐edited or typeset. Technical support issues arising from supporting information (other than missing files) should be addressed to the authors.

Supporting Information

## Data Availability

The data that support the findings of this study are available in the Supporting Information of this article.
