# Supplementary material for: Covalent Template‐Directed Synthesis of a Spoked 18‐Porphyrin Nanoring
Source: Angew Chem Int Ed Engl. 2023 Mar 24;62(18):e202302114. doi: 10.1002/anie.202302114 (PMC10947019; doi:10.1002/anie.202302114)
Supplement: Supplementary file 1 — Supporting Information [file ANIE-62-0-s001.pdf]

Angewandte Chemie
Eine Zeitschrift der Gesellschaft Deutscher Chemiker

Supporting Information

Covalent Template-Directed Synthesis of a Spoked 18-Porphyrin Nanoring

M. A. Majewski, W. Stawski, J. M. Van Raden, M. Clarke, J. Hart, J. N. O'Shea, A. Saywell, H. L. Anderson**

Table of Contents

Section 1. General Methods S2
Section 2. Synthetic Procedures S3
Section 3. Comparison of UV-vis-NIR Spectra S10
Section 4. NMR and Mass Spectra S11
Section 5. STM Studies S35
Section 6. Calculated Molecular Geometries S39
Section 7. References S50

1. General Methods

Dichloromethane, toluene and DMF for reactions were obtained from an MBraun MBSPS-5-BenchTop solvent purification system (SPS) under nitrogen. Chloroform-*d* for NMR was stored over K_2CO_3 and passed through a short neutral alumina plug prior to use. Bis(pinacolato)diboron was recrystallized from warm pentane. All other reagents and solvents were obtained from commercial suppliers and used as received unless otherwise stated. 1,4-Diiodo-2,5-dioctylbenzene **S1**, 3,5-bis(trihexylsilyl)benzaldehyde and 5-(4,4,5,5-tetramethyl-1,3,2-dioxaborolan-2-yl)-10,20-bis(3,5-dioctyloxyphenyl)porphyrinato zinc(II) **5** were prepared as described in the literature.[1-3]

Thin-layer chromatography (TLC) was carried out using commercially available (Merck) aluminum sheets precoated with silica gel with fluorescence indicator and visualized under UV light at 254 or 360 nm. Purification by column chromatography was carried out on silica gel (SiO_2, 60 Å, 40−63 µm, Merck). Size exclusion chromatography (SEC) was performed on gravity columns filled with Bio Beads S-X1. Analytical GPC was carried out using Jaigel-3H-A (8 × 500 mm) and Jaigel-4H-A (8 × 500 mm) columns in THF + 1% pyridine as eluent with a flow rate of 1.0 mL/min. Semipreparative GPC was carried out on a Shimadzu recycling GPC system equipped with a LC-20 AD pump, SPD20A UV detector and a set of JAIGEL 3H (20 × 600 mm) and JAIGEL 4H (20 × 600 mm) columns in toluene + 1% pyridine as the eluent at a flow rate of 3.5 mL/min. Pyridine after separations was removed fully on a rotary evaporator by adding toluene and evaporating to dryness and repeating the process four times.

^1H and ^{13}C NMR spectra were recorded on either a Bruker AVIII HD 400, a Bruker AVIII HD 500, or a Bruker AVIII 600 with a broadband cryo-probe. Chemical shift values are quoted in ppm and coupling constants (*J*, reported $^3J_{H\text{-}H}$ if not indicated differently) in Hertz to the nearest 0.1 Hz. Multiplicity is described as follows: s – singlet, d – doublet, t – triplet, m – multiplet, br. – broadened. ^1H and ^{13}C NMR spectra are referenced against the residual solvent peak ($CHCl_3$ δ_H = 7.26 ppm, $CDCl_3$ δ_C = 77.16 ppm). UV-vis-NIR measurements were carried out in a 1 cm path length glass cuvette at 298 K using either a Perkin Lambda 20 or a Jasco V770 spectrophotometer (for NIR-absorbing compounds). Mass spectra were recorded using MALDI-TOF method using Bruker Autoflex instrument with DCTB as a matrix. Calibration was performed before each measurement using Peptide Standard II for the 700–3500 Da Protein Standard I for the 5–20 kDa window (Bruker). Mass spectra of compounds with molecular weight up to 3.5 kDa were measured in a reflectron mode whereas for larger mass a linear mode was used.

2. Synthetic Procedures

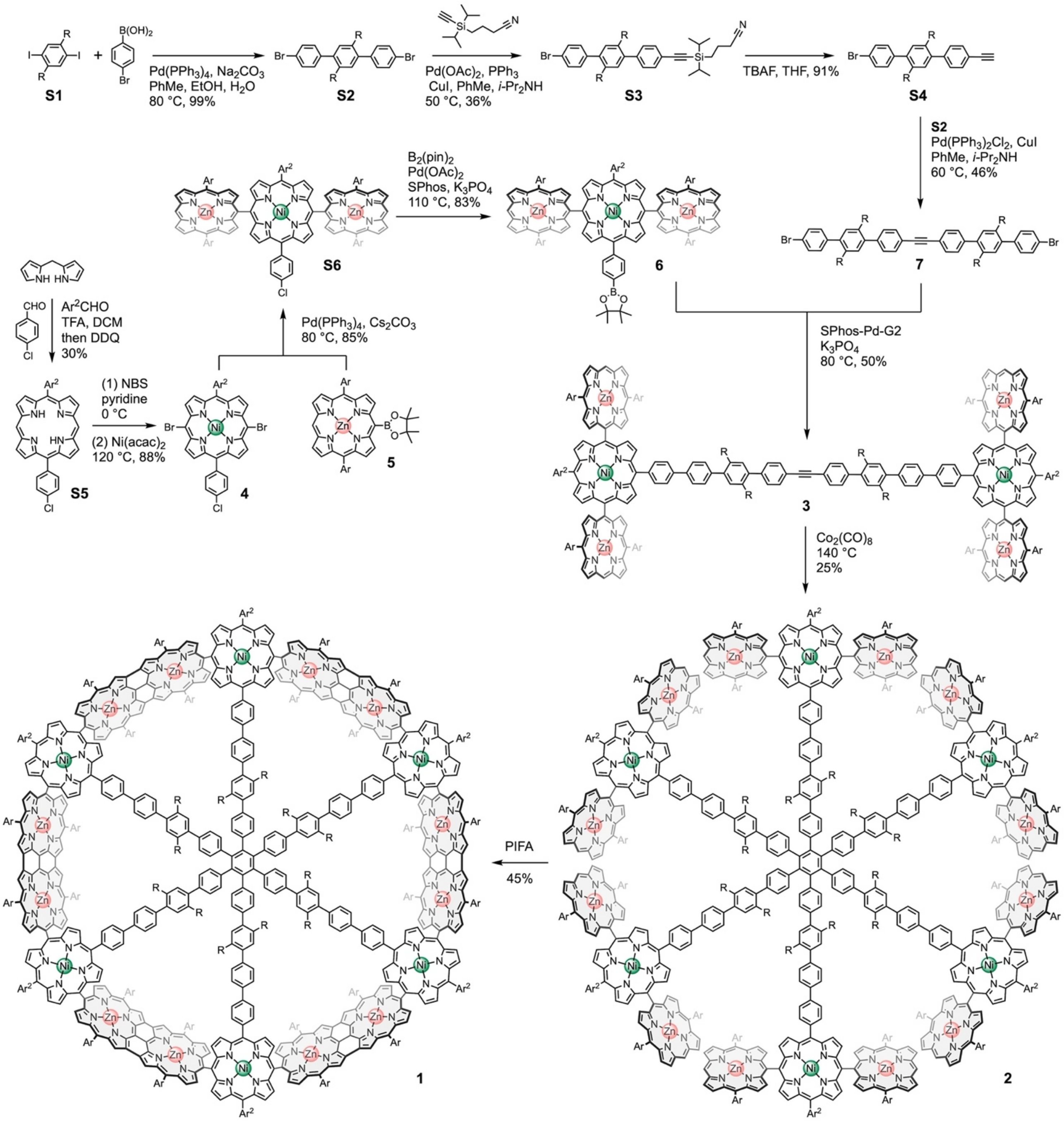

Scheme S1. Summary of synthetic route. (Ar = 3,5-bis(octyloxy)phenyl, Ar^2 = 3,5-bis(tri-*n*-hexylsilyl)phenyl, R = *n*-C_8H_{17}.)

Synthesis of 5-(3,5-bis(trihexylsilyl)phenyl)-15-(4-chlorophenyl)porphyrin S5.

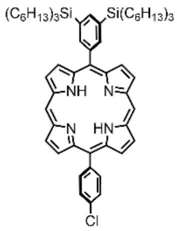

3,5-Bis(trihexylsilyl)benzaldehyde[2] (1.52 g, 1 equiv., 2.26 mmol), 4-chlorobenzaldehyde (1.27 g, 4 equiv., 9.06 mmol) and dipyrromethane (1.65 g, 5 equiv., 11.3 mmol) were dissolved in dichloromethane (1.5 L). The solution was purged with nitrogen, then trifluoroacetic acid (3.1 g, 2.1 mL, 12 equiv., 27.2 mmol) was added and the mixture was stirred at room temperature for 3 h in the dark. DDQ (3.85 g, 7.5 equiv., 17.0 mmol) was added and stirring was continued for 40 min. Finally, triethylamine (8.02 g, 11.0 mL, 35 equiv., 79.2 mmol) was added and the mixture was stirred for an additional 30 min. The solution was concentrated and passed through two silica plugs using dichloromethane/petroleum ether (1:4). Solvents were removed giving a mixture of different porphyrins. The crude mixture was purified on silica gel, first with

DCM/PE, 1:10 to separate bis-THS condensed byproduct, then the desired product was eluted using DCM/PE, 1:5, followed by another byproduct (bis(chlorophenyl)porphyrin) eluted in DCM. Organic fractions were concentrated, precipitated from DCM/methanol on a rotary evaporator and dried, yielding **S5** as a purple semisolid (710 mg, 30%).

^{1}H NMR (400 MHz, $CDCl_3$, 298 K): δ_H 10.34 (s, 2H, *meso*-H), 9.43 (d, *J* = 4.4 Hz, 2H, β-H), 9.41 (d, *J* = 4.4 Hz, 2H, β-H), 9.10 (d, *J* = 4.4 Hz, 2H, β-H), 9.07 (d, *J* = 4.4 Hz, 2H, β-H), 8.36 (d, *J* = 1.2 Hz, 2H, *o*-Ph), 8.23 (d, *J* = 8.2 Hz, 2H, Ph(Cl)-H), 8.03 (t, *J* = 1.2 Hz, 1H, *p*-Ph), 7.81 (d, *J* = 8.2 Hz, 2H, Ph(Cl)-H), 1.51 (p, 12H, overlapping with H_2O, CH_2), 1.39 (p, 12H, CH_2), 1.32 (m, 24H, CH_2), 0.95 (m, 12H, CH_2), 0.88 (t, *J* = 6.9 Hz, 18H, CH_3), –3.08 (s, 2H, NH).

^{13}C NMR (151 MHz, $CDCl_3$) δ_C 147.5, 146.9, 145.3, 145.2, 141.0, 140.0, 139.4, 139.3, 135.8, 135.4, 134.2, 131.8, 131.5, 131.3, 130.6, 127.2, 120.8, 117.1, 105.3, 33.5, 31.6, 24.0, 22.6, 14.1, 12.7.

HRMS (MALDI): *m/z* calcd for $C_{68}H_{97}ClN_4Si_2$: 1060.694 $[M]^+$; found: 1060.505.

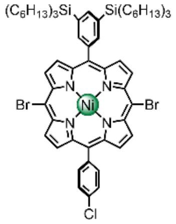

<u>Synthesis of 5-(3,5-bis(trihexylsilyl)phenyl)-15-(4-chlorophenyl)porphyrinato nickel(II) 4.</u> Porphyrin **S5** (1.29 g, 1 equiv., 1.22 mmol) was dissolved in chloroform (235 mL) and pyridine (12 mL), then NBS (432 mg, 2 equiv., 2.43 mmol) was added as a $CHCl_3$/pyridine solution (12 mL + 0.6 mL) and the mixture was stirred for 3 h at 0 °C. The resulting crude was subjected to a short chromatography column in chloroform. The first fraction was collected and evaporated to leave a dark green solid and was used without further purification. The crude product and nickel acetylacetonate dihydrate (2.11 g, 6 equiv., 7.21 mmol) were dissolved in xylenes (120 mL), purged with argon, and heated at 120 °C on an oil bath for 4 h. The crude product was passed through a short silica plug in DCM and precipitated from DCM and MeOH on a rotary evaporator to give a red residue. Purification by column chromatography using DCM/PE (1:10), evaporation of organic fractions and additional was with MeOH gave **4** as a red semisolid (1.35 g, 88%).

^{1}H NMR (400 MHz, $CDCl_3$, 298 K): δ_H 9.46 (d, *J* = 4.9 Hz, 2H, β-H), 9.44 (d, *J* = 4.9 Hz, 2H, β-H), 8.72 (d, *J* = 4.9 Hz, 2H, β-H), 8.68 (d, *J* = 4.9 Hz, 2H, β-H), 8.01 (d, *J* = 1.1 Hz, 2H, *o*-Ph), 7.92 (t, *J* = 1.1 Hz, 1H, *p*-Ph), 7.87 (d, *J* = 8.3 Hz, 2H, Ph(Cl)-H), 7.68 (d, *J* = 8.3 Hz, 2H, Ph(Cl)-H), 1.47–1.26 (overlapping m, 60H, CH_2), 0.90–0.84 (m, 18H, CH_3).

^{13}C NMR (151 MHz, $CDCl_3$, 298 K): δ_C 144.0, 142.9, 142.8, 142.7, 139.7, 139.6, 138.5, 138.0, 135.6, 134.6, 134.5, 134.0, 133.7, 133.5, 133.2, 127.3, 121.5, 118.1, 102.8, 33.5, 31.6, 24.0, 22.6, 14.1, 12.6.

HRMS (MALDI): *m/z* calcd for $C_{68}H_{93}Br_2ClN_4NiSi_2$: 1272.434 $[M]^+$; found 1272.474.

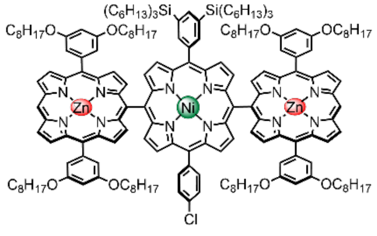

<u>Synthesis of porphyrin trimer S6.</u> 5-(4,4,5,5-Tetramethyl-1,3,2-dioxaborolan-2-yl)-10,20-bis(3,5-dioctyloxyphenyl)porphyrinato zinc(II) **5**[3] (170 mg, 2.2 equiv., 0.15 mmol), **4** (85 mg, 1.0 equiv., 67 µmol) and cesium carbonate (0.13 g, 6 equiv., 0.40 mmol) were placed in a dry Schlenk vessel and dried under vacuum at 40 °C for 1 h, then purged with argon 5 times. Tetrakis(triphenylphosphine)palladium (23 mg, 0.3 equiv., 20 µmol), toluene (12.0 mL) and DMF (6.00 mL) were added and the mixture was purged with argon. The reaction was heated at 80 °C on an oil bath for 18 h under argon. After completion, it was passed through a short silica gel plug in 10% EtOAc in DCM and evaporated. The crude was subjected to column chromatography in DCM/PE (1:2), followed by SEC column in 1% pyridine in toluene to give **S6** as a red solid (180 mg, 85%).

^{1}H NMR (400 MHz, $CDCl_3$, 298 K): δ_H 10.38 (s, 2H, *meso*-H), 9.49 (d, *J* = 4.4 Hz, 4H, Zn-β-H), 9.28 (d, *J* = 4.4 Hz, 4H, Zn-β-H), 8.93 (d, *J* = 4.4 Hz, 4H, Zn-β-H), 8.56 (d, *J* = 5.0 Hz, 2H, Ni-β-H), 8.52 (d, *J* = 5.0 Hz, 2H, Ni-β-H), 8.30 (d, *J* = 4.4 Hz, 4H, Zn-β-H), 8.12 (two overlapping d, 4H, Ni-β-H and *o*-Ph-Ni), 8.08 (d, *J* = 5.0 Hz, 2H, Ni-β-

H), 7.99 (d, J = 8.9 Hz, 2H, Ph(Cl)-H), 7.72 (s, 1H, p-Ph-Ni), 7.52 (d, J = 8.9 Hz, 2H, Ph(Cl)-H), 7.43 (bd, 8H, o-Ph-Zn), 6.86 (t, J = 2.2 Hz, 4H, p-Ph-Zn), 4.10 (bt, 16H, OOct-CH_2), 1.83 (m, 16H, OOct-CH_2), 1.46 (m, 16H, OOct -CH_2), 1.37–1.11 (overlapping m, 74 H, -CH_2), 1.12 (m, 16H, -CH_2), 1.05-0.95 (overlapping m, 22H, -CH_2), 0.81 (t, J = 6.7 Hz, 24H, OOct -CH_3), 0.70 (m, 12H, THS -CH_2), 0.47 (t, J = 6.8 Hz, 18H, THS -CH_3)

^{13}C NMR (151 MHz, $CDCl_3$) δ 158.5, 154.2, 150.7, 150.1, 149.8, 147.8, 147.8, 144.4, 143.5, 142.8, 139.7, 139.6, 139.4, 139.1, 135.2, 134.8, 134.7, 134.2, 133.7, 132.8, 132.4, 132.4, 132.0, 131.6, 127.1, 122.4, 121.5, 119.2, 119.1, 117.9, 114.5, 114.4, 106.9, 101.2, 68.5, 33.4, 31.9, 31.5, 29.5 (two overlapping peaks), 29.4, 26.3, 24.0, 22.8, 22.5, 14.2, 14.0, 12.6.

HRMS (MALDI): m/z calcd for $C_{196}H_{259}ClN_{12}NiO_8Si_2Zn_2$: 3185.739 $[M]^+$; found 3185.411.

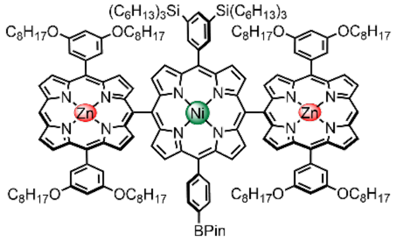

<u>Synthesis of porphyrin trimer 6.</u> Porphyrin trimer **S6** (450 mg, 1 equiv., 141 µmol), bis(pinacolato)diboron (358 mg, 10 equiv., 1.41 mmol), $Pd(OAc)_2$ (31.6 mg, 1 equiv., 141 µmol), potassium phosphate (239 mg, 8 equiv., 1.13 mmol) and SPhos (116 mg, 2 equiv., 282 µmol) were placed in a dry flask, degassed and purged with argon 3 times. Then dry 1,4-dioxane (20.0 mL) was added, followed by degassing by three freeze-pump-thaw cycles and the mixture was stirred at 110 °C for 16 h. After removing the solvent in vacuo, the residue was dissolved in chloroform, washed with brine 3 times, dried over anhydrous Na_2SO_4 and evaporated. The crude material was subjected to column chromatography in 2:3 DCM/PE with 1% pinacol added to separate fraction containing deborylated compound, then in DCM separate the main fraction, which was then recrystallized from DCM/MeOH and subjected to SEC chromatography in THF. The main fraction was collected, solvent was removed and recrystallization from DCM/MeOH provided **6** as a red solid (385 mg, 83%).

^{1}H NMR (400 MHz, $CDCl_3$, 298 K): δ_H 10.38 (s, 2H, meso-H), 9.49 (d, J = 4.4 Hz, 4H, Zn-β-H), 9.27 (d, J = 4.4 Hz, 4H, Zn-β-H), 8.93 (d, J = 4.5 Hz, 4H, Zn-β-H), 8.55 (d, J = 5.1 Hz, 2H, Ni-β-H), 8.54 (d, J = 5.1 Hz, 2H, Ni-β-H), 8.31 (d, J = 4.5 Hz, 4H, Zn-β-H), 8.12 (two overlapping d, 4H, Ni-β-H and o-Ph-Ni), 8.05 (two overlapping d, 4H, Ni-β-H and Ph(BPin)-H), 7.97 (d, J = 8.6 Hz, 2H, Ph(BPin)-H), 7.72 (s, 1H, p-Ph-Ni), 7.43 (m, 8H, o-Ph-Zn), 6.85 (t, J = 2.3 Hz, 4H, p-Ph-Zn), 4.10 (bt, 16H, OCH_2), 1.86-1.79 (m, 16H, CH_2), 1.50–1.42 (m, 16H, CH_2), 1.37–1.20 (m, 72H, CH_2), 1.31 (s, 12H, Bpin), 1.19–1.09 (m, 12H, CH_2), 1.03–0.96 (m, 24H, CH_2), 0.82 (t, J = 6.7 Hz, 24H, OOct -CH_3), 0.73–0.65 (m, 12H, CH_2), 0.47 (t, J = 6.8 Hz, 18H, THS -CH_3).

^{13}C NMR (151 MHz, $CDCl_3$) δ_C 158.5, 154.3, 150.7, 150.1, 149.9, 147.7, 144.5, 144.1, 143.4, 143.0, 139.6, 139.3, 139.3, 135.1, 134.7, 134.6, 133.8, 133.4, 133.2, 132.8, 132.4, 132.3, 132.0, 122.3, 121.5, 120.8, 118.9, 118.1, 114.52, 114.47, 106.9, 101.2, 84.1, 68.5, 33.4, 32.0, 31.5, 29.6, 29.4, 26.3, 25.1, 24.0, 22.8, 22.5 (two overlapping peaks), 14.2, 14.0, 12.6. Two aromatic peaks are not visible, they either overlap with some others or correspond to quaternary carbons and are very weak.

HRMS (MALDI): m/z calcd for $C_{202}H_{271}BN_{12}NiO_{10}Si_2Zn_2$: 3277.863 $[M]^+$; found 3277.932.

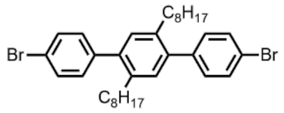

<u>Synthesis of 4,4''-dibromo-2',5'-dioctyl-1,1':4',1''-terphenyl S2.</u> 1,4-Diiodo-2,5-dioctylbenzene **S1**[1] (1.00 g, 1 equiv., 1.80 mmol), (4-bromophenyl)boronic acid (1.09 g, 3 equiv., 5.41 mmol) and sodium carbonate (765 mg, 4 equiv., 7.22 mmol) were dissolved in a mixture of toluene (20.0 mL), ethanol (4.00 mL) and water (4.00 mL), then degassed via three freeze-pump-thaw cycles. $Pd(PPh_3)_4$ (104 mg, 0.05 equiv., 90.2 µmol) was then added and the reaction stirred at 80 °C for 18 h. The crude mixture was allowed to cool, extracted with DCM from water, washed with brine, dried over anhydrous Na_2SO_4, evaporated and subjected to column chromatography in PE 40/60, after evaporation providing **S2** as a white solid (1.10 g, 99%).

^{1}H NMR (400 MHz, $CDCl_3$, 298 K): δ_H 7.55 (d, J = 8.1 Hz, 4H, Ph), 7.22 (d, J = 8.1 Hz, 4H, Ph), 7.07 (s, 2H, Ph), 2.51 (t, J = 8.1 Hz, 4H, -CH_2), 1.46–1.40 (m, 4H, -CH_2), 1.29-1.17 (m, 20H, -CH_2), 0.86 (t, J = 6.8 Hz, 6H, -CH_3).

^{13}C NMR (150 MHz, $CDCl_3$, 298 K): δ_C 140.7, 139.8, 137.5, 131.2, 131.0, 130.8, 121.0, 32.5, 31.8, 31.4, 29.5, 29.2, 29.1, 22.6, 14.1.

HRMS (MALDI): *m/z* calcd for $C_{34}H_{44}Br_2$: 610.180 $[M]^+$; found 610.233.

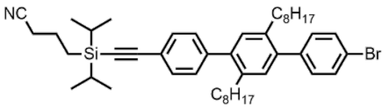

<u>Synthesis of 4-(((4''-bromo-2',5'-dioctyl-[1,1':4',1''-terphenyl]-4-yl)ethynyl)diisopropylsilyl)butanenitrile S3.</u> An oven dried and argon flushed Schlenk tube was charged with **S2** (400 mg, 1.0 equiv., 653 µmol), dry toluene (4.00 mL) and *i*Pr_2NH (4.00 mL) and the mixture was degassed by argon bubbling for 10 min. Then 4-(ethynyldiisopropylsilyl)butanenitrile (0.15 g, 0.17 mL, 1.1 equiv., 0.73 mmol), palladium(II) acetate (16.1 mg, 0.11 equiv., 71.8 µmol), triphenylphosphine (54.8 mg, 0.32 equiv., 209 µmol) and copper(I) iodide (37.3 mg, 0.3 equiv., 196 µmol) were added and the mixture was bubbled again for 5 min. The solution was stirred at 50 °C for 2.5 h, then allowed to cool, diluted with ethylacetate (60 mL), washed with brine and water, the organic phase dried over anhydrous Na_2SO_4 and evaporated. Column chromatography in DCM/PE (1:2) gave **S3** as yellow-white oily solid (175 mg, 36%), accompanied by recovered **S2** (130 mg) in the first fraction.

^{1}H NMR (400 MHz, $CDCl_3$, 298 K): δ_H 7.56 (d, *J* = 8.3 Hz, 2H, Ph), 7.53 (d, *J* = 8.3 Hz, 2H, Ph), 7.30 (d, *J* = 8.3 Hz, 2H, Ph), 7.23 (d, *J* = 8.3 Hz, 2H, Ph), 7.07 (s, 2H, Ph), 2.52 (m, 4H, alkyl), 2.45 (t, *J* = 7.0 Hz, 2H, alkyl), 1.90 (m, 2H, alkyl), 1.44 (m, 4H, alkyl), 1.27–1.09 (overlapping m, 36H, alkyl), 0.88–0.83 (m, 6H, alkyl).

^{13}C NMR (151 MHz, $CDCl_3$) δ_C 142.4, 140.8, 140.3, 139.8, 137.54, 137.52, 131.8, 131.2, 131.0, 130.79, 130.76, 129.3, 121.4, 121.0, 119.8, 107.9, 89.5, 32.5, 31.8, 31.8, 31.4, 31.3, 29.47, 29.45, 29.3, 29.2, 29.1, 29.1, 22.7, 22.6, 21.4, 20.8, 18.2, 18.0, 14.1, 11.8, 9.7.

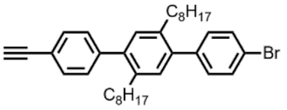

<u>Synthesis of 4-bromo-4''-ethynyl-2',5'-dioctyl-1,1':4',1''-terphenyl S4.</u> Terphenyl **S3** was dissolved in dry THF (6.00 mL) and TBAF in THF (1.0 M, 310 mg, 1.18 mL, 5 equiv., 1.18 mmol) was added. The reaction mixture was stirred at room temperature for 15 min and checked via TLC, after completion quenched with water (3 mL) and extracted with AcOEt, dried over anhydrous Na_2SO_4 and evaporated. Column chromatography in DCM/PE (1:6) gave pure **S4** as yellowish oil that solidified after few hours (120 mg, 91%).

^{1}H NMR (400 MHz, $CDCl_3$, 298 K): δ_H 7.56 (d, *J* = 8.4 Hz, 2H, Ph), 7.54 (d, *J* = 8.4 Hz, 2H, Ph), 7.31 (d, *J* = 8.5 Hz, 2H, Ph), 7.23 (d, *J* = 8.3 Hz, 2H, Ph), 7.08 (s, 1H, Ph), 7.07 (s, 1H, Ph), 3.12 (s, 1H, acet.), 2.53 (m, 4H, $-CH_2$), 1.44 (m, 4H, CH_2), 1.28–1.16 (overlapping m, 20H, $-CH_2$), 0.86 (two overlapping t, *J* = 6.8 Hz, 6H, $-CH_3$).

^{13}C NMR (151 MHz, $CDCl_3$, 298 K): δ_C 142.5, 140.7, 140.3, 139.8, 137.5, 137.5, 131.8, 131.2, 131.0, 130.8, 130.7, 129.3, 120.9, 120.5, 83.6, 32.5, 31.8, 31.4, 29.4, 29.2, 29.1, 22.6, 14.1.

HRMS (MALDI): *m/z* calcd for $C_{36}H_{45}Br$, 556.270 $[M]^+$; found 556.346

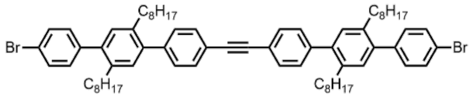

<u>Synthesis of 1,2-bis(4''-bromo-2',5'-dioctyl-[1,1':4',1''-terphenyl]-4-yl)ethyne 7.</u> An oven dried and argon flushed Schlenk tube was charged with **S2** (54.9 mg, 5 equiv., 89.7 µmol), dry toluene (0.50 mL) and *i*Pr_2NH (0.50 mL) and the mixture was degassed by argon bubbling for 10 min. Then **S4** (10.0 mg, 1 equiv., 17.9 µmol), bis(triphenylphosphine)palladium(II) dichloride (2.52 mg, 0.2 equiv., 3.59 µmol) and copper(I) iodide (0.68 mg, 0.2 equiv., 3.59 µmol) were added and the mixture was bubbled again for 5 min. The solution was stirred at 60 °C for 1 h on an oil bath, diluted with AcOEt, washed with brine, water, organic phase was dried over anhydrous Na_2SO_4 and evaporated. Column chromatography was then performed in pure PE, then switching to DCM/PE (1:2) which gave **7** as a white solid (9 mg, 46%), accompanied by recovered **6** in first fraction (in PE).

^{1}H NMR (400 MHz, $CDCl_3$, 298 K): δ_H 7.60 (d, *J* = 8.3 Hz, 4H, Ph), 7.56 (d, *J* = 8.3 Hz, 4H, Ph), 7.35 (d, *J* = 8.3 Hz, 4H, Ph), 7.23 (d, *J* = 8.3 Hz, 4H, Ph), 7.10 (s, 2H, Ph), 7.08 (s, 2H, Ph), 2.56 (m, 8H, $-CH_2$), 1.47 (m, 8H, $-CH_2$), 1.29–1.09 (m, 40H, $-CH_2$), 0.86 (overlapping t, 12H, $-CH_3$).

^{13}C NMR (151 MHz, $CDCl_3$, 298 K): δ_C 141.9, 140.8, 140.5, 139.8, 137.6, 137.5, 131.3, 131.2, 131.0, 130.8, 129.4, 121.8, 121.0, 89.7, 32.63, 32.58, 31.9, 31.4, 29.52, 29.49, 29.3, 29.2, 22.7, 14.1.

HRMS (MALDI): *m/z* calcd for $C_{70}H_{88}Br_2$: 1086.525 $[M]^+$; found: 1086.471.

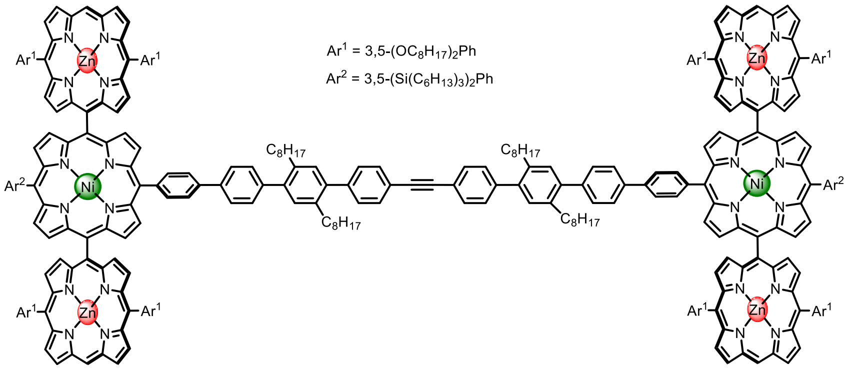

<u>Synthesis of hexaporphyrin tolan 3.</u> Tolan **7** (22.0 mg, 1 equiv., 20.2 µmol), porphyrin trimer **6** (199 mg, 3 equiv., 60.6 µmol) and SPhos-Pd-G2 (14.6 mg, 1 equiv., 20.2 µmol), were placed in a dry Schlenk and dried under high vacuum for 30 min, then dry 1,4-dioxane (6.0 mL) was added. A degassed, aqueous solution of potassium phosphate (2.0 M, 0.3 mL, 30 equiv., 606 µmol) was then added and the mixture was subjected to degassing by four freeze-pump-thaw cycles. The reaction mixture was heated under argon at 80 °C on an oil bath for 16 h, extracted with DCM/H_2O, the organic phase was dried over anhydrous Na_2SO_4 and evaporated. The crude mixture was subjected to column chromatography (DCM/PE, 1:1, after elution of smaller by-products change to DCM), followed by preparative GPC separation (1% pyridine in toluene), evaporation of all solvents and precipitation from DCM/MeOH on a rotary evaporator to yield **3** as a red solid (73 mg, 50%).

^{1}H NMR (600 MHz, $CDCl_3$, 298 K): δ_H 10.36 (s, 4H), 9.48 (d, *J* = 4.3 Hz, 8H), 9.27 (d, *J* = 4.3 Hz, 8H), 8.93 (d, *J* = 4.3 Hz, 8H), 8.64 (d, *J* = 5.1 Hz, 4H), 8.56 (d, *J* = 5.1 Hz, 4H), 8.31 (d, *J* = 4.3 Hz, 8H), 8.19–8.10 (overlapping t,d,d, 12H), 8.05 (d, *J* = 5.1 Hz, 4H), 7.85 (d, *J* = 8.8 Hz, 4H), 7.77 (d, *J* = 8.8 Hz, 4H), 7.75 (s, 2H), 7.57 (d, *J* = 8.8 Hz, 4H), 7.45 (d, *J* = 2.3 Hz, 16H), 7.43 (d, *J* = 8.8 Hz, 4H), 7.33 (d, *J* = 8.8 Hz, 4H), 7.14 (s, 2H), 7.10 (s, 2H), 6.87 (t, *J* = 2.3 Hz, 8H), 4.14–4.09 (overlapping m, 32H), 2.56 (overlapping m, 8H), 1.85 (p, *J* = 6.8 Hz, 32H), 1.52–1.43 (overlapping, 32H), 1.39–1.19 (overlapping m, 168H), 1.19–1.08 (overlapping m, 60H), 1.08–0.98 (overlapping m, 48H), 0.86–0.79 (m, 48H), 0.76–0.71 (overlapping m, 32H), 0.52 (t, *J* = 7.1 Hz, 36H).

^{1}H signals assignment – see Fig. S26.

^{13}C NMR (151 MHz, $CDCl_3$) δ 158.4, 154.2, 150.6, 150.0, 149.8, 147.9, 144.7, 143.5, 143.4, 143.1, 142.1, 141.2, 140.7, 140.3, 140.1, 139.6, 139.3, 139.1, 137.8, 137.6, 136.1, 135.1, 134.6, 134.3, 133.7, 132.7, 132.3, 131.9, 131.4, 131.1, 130.9, 130.0, 129.5, 126.9, 125.4, 122.5, 122.2, 121.8, 121.4, 120.4, 119.2, 117.8, 114.6, 114.5, 106.8, 101.0, 89.8, 68.5, 33.4, 32.8, 31.9, 31.9, 31.5, 29.63, 29.59, 29.55, 29.54, 29.53, 29.4, 29.3, 29.24, 29.20, 26.3, 24.0, 22.8, 22.7, 22.5, 14.2, 14.0, 12.6. Seven alkyl peaks are not resolved as they overlap with other peaks in the alkyl region.

MS (MALDI): *m/z* calcd for $C_{462}H_{606}N_{24}Ni_2O_{16}Si_4Zn_4$: 7243.235 $[M]^+$; found: 7245.023.

UV-vis: (CH_2Cl_2, 298 K) λ, log ε: 411 (6.07), 463 (5.91), 551 (5.49).

Synthesis of 18-porphyrin radial oligomer 2. Tolan **3** (23 mg, 1 equiv., 3.18 µmol) and octacarbonylcobalt(II)* (1.1 mg, 1.0 equiv, 3.18 µmol) were placed in a dry 1 mL pressure tube with Teflon cap, atmosphere was changed to argon, dry toluene (90 µL) was added. The tube was sealed under stream of argon, pre-stirred for 15 min and then heated at 140 °C on an oil bath for 16 h. After completion, the crude mixture was passed through a short silica plug in DCM and evaporated, then subjected to GPC separation (1% pyridine in toluene) followed by evaporation of all solvents and precipitation from DCM/MeOH on a rotary evaporator to yield cyclotrimer **2** as a dark red solid (5.75 mg, 25%).

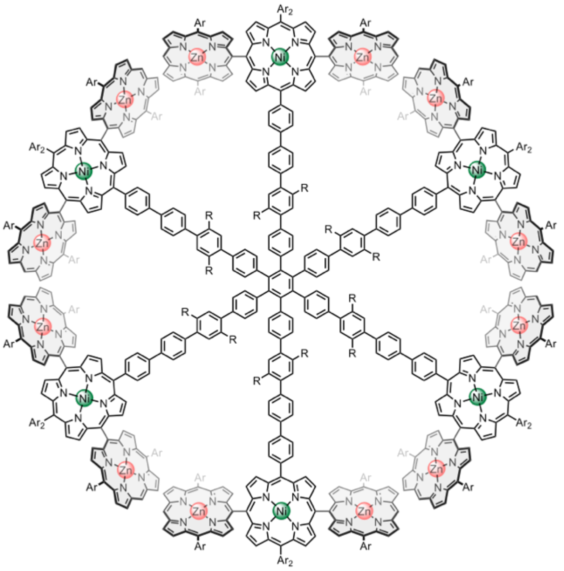

*Note: $Co_2(CO)_8$ is not stable in the air and care must be taken when handling and storing the chemical – it has to be orange/brown. A black/violet color indicates substantial decomposition and the use of the catalyst of that quality was found not to lead to formation of the desired product. We recommend storing this compound in separate vials under argon in a freezer to avoid decomposition of the whole package.

^{1}H NMR (600 MHz, CD_2Cl_2): δ_H 10.20 (s, 12H), 9.33 (d, J = 4.6 Hz, 24H), 9.15 (d, J = 4.6 Hz, 24H), 8.87 (d, J = 4.5 Hz, 24H), 8.61 (d, J = 5.0 Hz, 12H), 8.58 (d, J = 5.0 Hz, 12H), 8.28 (d, J = 4.5 Hz, 24H), 8.18 (s, 12H), 8.12 (d, J = 4.9 Hz, 12H), 8.10–8.05 (overlapping d and t, 24H), 7.82 (s, 6H), 7.75 (d, J = 8.4 Hz, 12H), 7.62 (d, J = 8.4 Hz, 12H), 7.24 (d, J = 6.6 Hz, 48H), 7.22 (overlapping d, 12H), 6.91 (d, J = 8.4 Hz, 12H), 6.87 (s, 6H), 6.79 (overlapping s and d, 18H), 6.62 (t, J = 2.3 Hz, 24H), 3.91 (m, 48H), 3.85 (m, 48H), 2.37 (m, 12H), 2.22 (m, 12H), 1.72 (p, J = 6.9 Hz, 48H), 1.58 (p, J = 7.5 Hz, 48H), 1.38 (overlapping p, J = 7.5 Hz, 48H), 1.33–1.20 (overlapping m, 348H), 1.20–1.14 (overlapping m, 96H), 1.14–0.97 (overlapping m, 360H), 0.95–0.83 (overlapping m, 96H), 0.83–0.74 (overlapping m and t, 144H), 0.63 (t, J = 7.1 Hz, 72H, OOct -CH_3), 0.49 (t, J = 7.1 Hz, 108H, THS -CH_3).

^{1}H signals assignment – see Fig. S33.

^{13}C NMR (151 MHz, CD_2Cl_2, 298 K): δ_C 158.1, 153.9, 150.4, 149.8, 149.6, 147.6, 144.1, 143.3, 143.0, 141.29, 141.27, 140.7, 140.6, 139.9, 139.0, 138.9, 138.6, 137.4, 137.1, 135.2, 134.2, 133.3, 132.5, 132.1, 131.7, 131.2, 130.5, 129.7, 127.5, 126.4, 125.2, 122.3, 121.3, 120.5, 118.8, 117.6, 114.5, 114.4, 106.6, 100.9, 68.4, 68.3, 33.3, 32.4, 31.9, 31.8, 31.7, 31.6, 31.4, 29.7, 29.34, 29.24, 29.21, 29.17, 29.13, 29.04, 28.95, 25.6, 25.9, 23.8, 22.6, 22.44, 22.37, 13.8, 13.7, 13.64, 13.61, 12.4. Two of the alkyl signals are not resolved due to overlapping with other signals. Seven aromatic signals are not visible too due to low signal to noise ratio which results from line broadening and aggregation, even though we recorded the spectrum for a long time using a cryoprobe (Fig. S35). To compensate for that, we recorded a series of 2D experiments (see Fig. S37–S41).

MS (MALDI): *m/z* calcd for $C_{1386}H_{1818}N_{72}Ni_6O_{48}Si_{12}Zn_{12}$: 21729.705 $[M]^+$; found: 21738.003.

UV-vis: (CH_2Cl_2, 298 K) λ, log ε: 412 (6.33), 464 (6.18), 552 (5.78).

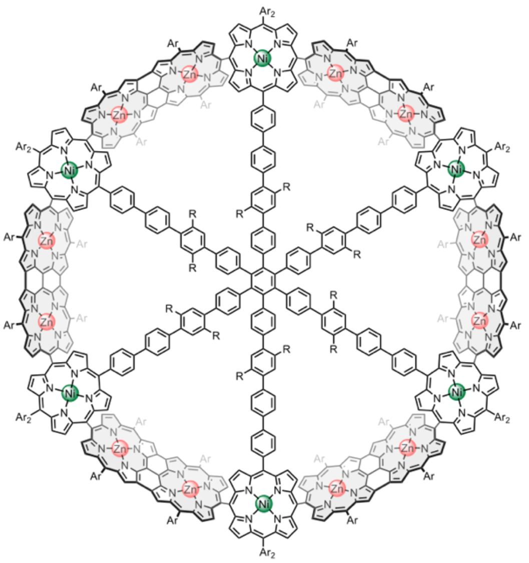

<u>**Synthesis of nanoring 1.**</u> Porphyrin octadecamer **2** (10.0 mg, 0.46 µmol, 1 equiv.) was dissolved in dry, degassed DCM (39 mL) under inert gas atmosphere and the solution was cooled to –78 °C. A solution of bis(trifluoroacetoxy)iodobenzene (PIFA, 2.97 mg, 6.9 µmol, 15 equiv.) in dry, degassed DCM (1 mL) was added. The cooling bath was removed and the mixture was stirred at room temperature for 2.5 h which was followed by a color change from orange to turquoise. After quenching the reaction by adding a suspension of $NaBH_4$ (1.0 mg, 27.6 µmol, 60 equiv.) in MeOH (0.3 mL), the solvents were evaporated and the solid residue was washed with methanol. Separation by GPC (1% pyridine in toluene) followed by evaporation of all solvents and precipitation from DCM/MeOH on a rotary evaporator and filtration gave nanoring **1** as a turquoise solid (4.5 mg, 45%).

^{1}H NMR (600 MHz, $CDCl_3$ + 1% pyridine-d_5, 298 K): δ_H 9.14 (br., 12H), 8.82 (br., 12H), 8.30 (br., 12H), 8.25 (br., 12H), 7.89 (overlapping s and d*, 18H), 7.76 (s, 6H), 7.73 (two overlapping d*, 24H), 7.43 (br. d, 24H), 7.35 (d*, 12H), 7.10 (br., 24H), 7.00 (br., 18H), 6.94 (br., 24H), 6.90 (br., 12H), 6.79 (br., 48H), 6.58 (br, 24H), 3.99 (m, 48H), 3.94 (m, 48H), 2.47 (m, 12H), 2.36 (m, 12H), 1.78 (m, 48H), 1.69 (m, 48H), 1.57–0.96 (overlapping m, 720H), 0.91–0.78 (overlapping m, 324H), 0.78 – 0.67 (overlapping t, 216H).

*The *para*-phenylene signals from the template core become more resolved when the spectrum is measured in $C_2D_2Cl_4$ at 100 °C (Fig. S44). Partial ^{1}H signals assignment – see Fig. S42. Due to substantial broadening and overlapping of signals it was not possible to assign all of them.

^{13}C NMR (from ^{1}H-^{13}C HSQC experiment, 151 MHz, $CDCl_3$ + 1% pyridine-d_5, 298 K): δ_C 139.8, 139.4, 133.9, 132.7, 131.6, 131.0, 130.8, 130.7, 129.8, 127.8, 126.9, 126.4, 112.9, 112.2, 100.4, 68.2, 40.1, 36.8, 32.9, 32.7, 31.7, 29.6, 29.3, 27.4, 26.5, 26.2, 24.0, 22.6, 20.1, 19.4, 14.1, 12.7. We did not manage to measure ^{13}C NMR spectrum directly due to substantial line broadening and aggregation. Instead, we measured a ^{1}H-^{13}C HSQC NMR spectrum.

HRMS (MALDI): *m/z* calcd for $C_{1386}H_{1782}N_{72}Ni_6O_{48}Si_{12}Zn_{12}$: 21693.424 [*M*]$^+$; found: 21693.111.

UV-vis-NIR: (CH_2Cl_2, 298 K) λ, log ε: 413 (5.64), 618 (5.55), 934 (4.77), 1054 (4.93).

3. Comparison of UV-vis-NIR spectra

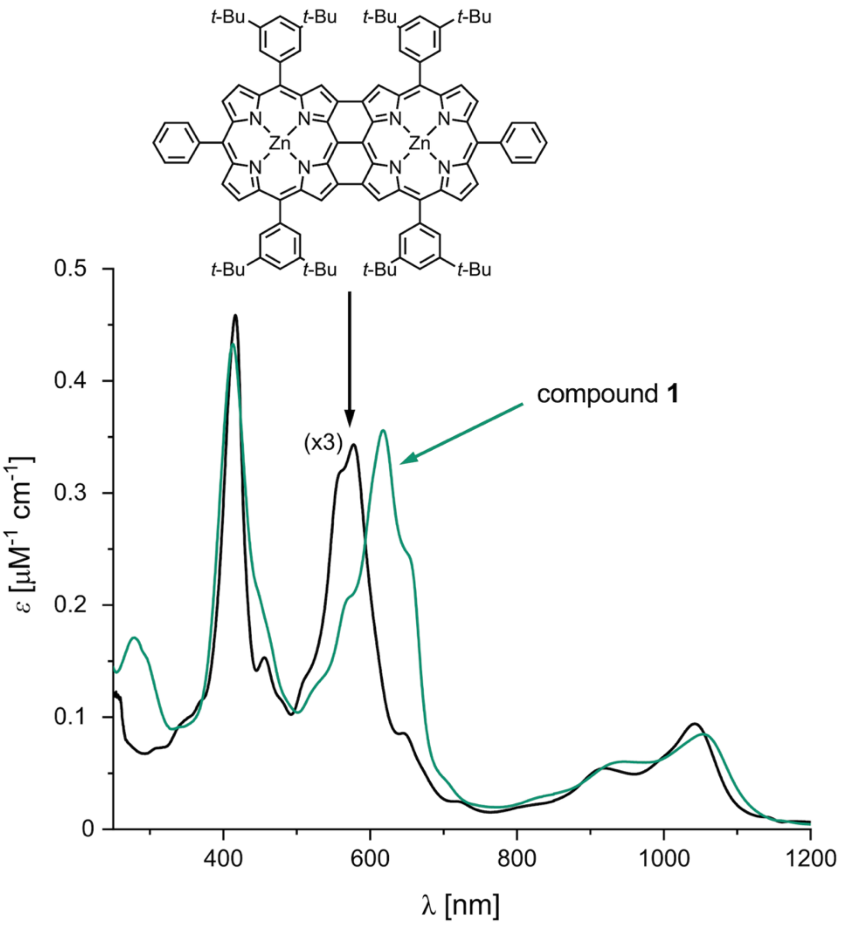

Figure S1. Comparison of the UV-vis-NIR spectrum of porphyrin 18-mer **1** (green line) with that of an edge-fused porphyrin dimer (black line), both spectra recorded in CH_2Cl_2 at 298 K.

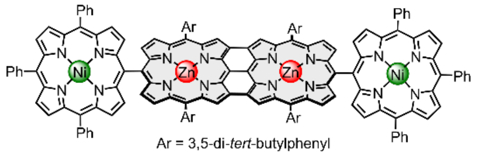

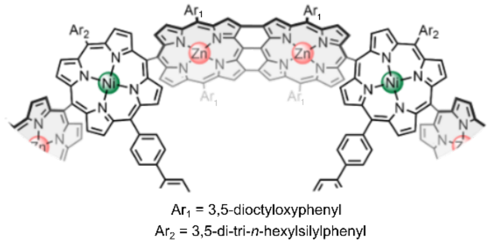

1055 nm	1054 nm
938 nm	934 nm
602 nm	618 nm
416 nm	413 nm

Figure S2. Comparison between the observed absorption maxima for **1** (right) and for a known linear tetramer (left).[4]

4. NMR and Mass Spectra

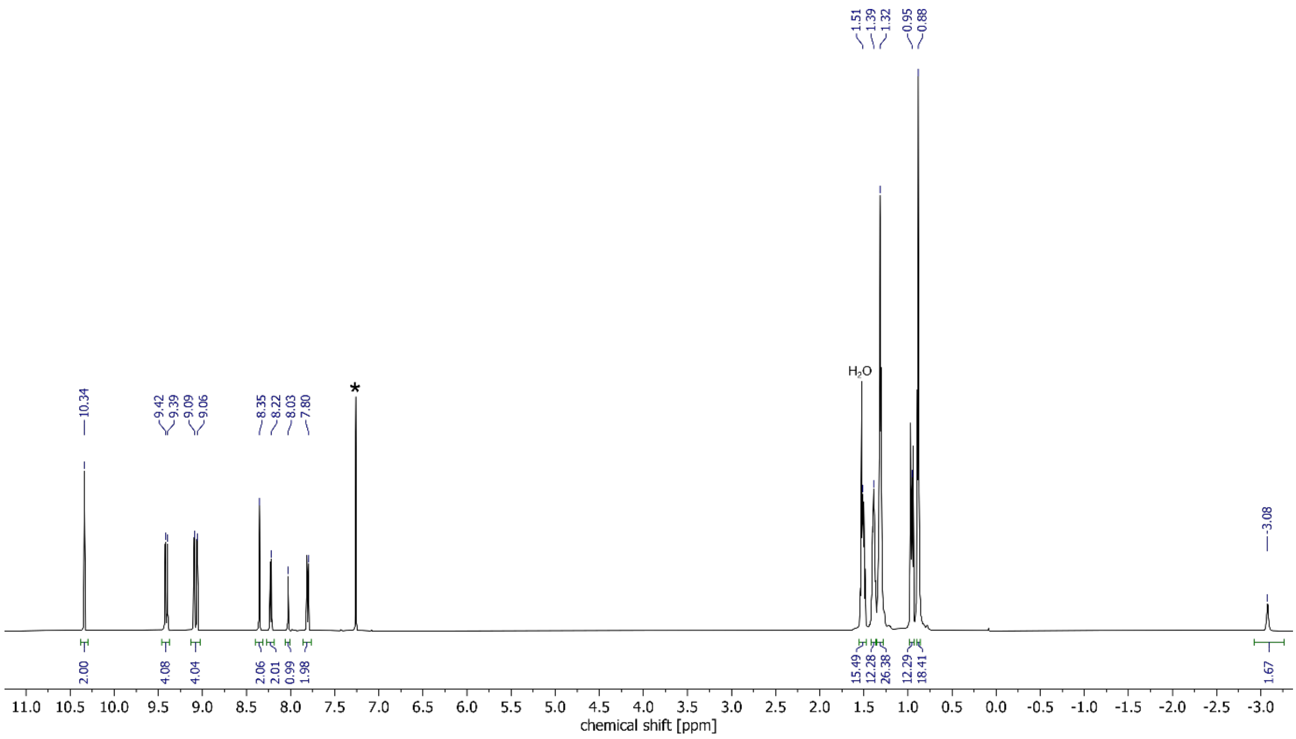

Figure S3. ^{1}H NMR spectrum of **S5**, $CDCl_3$, 600 MHz, 298 K.

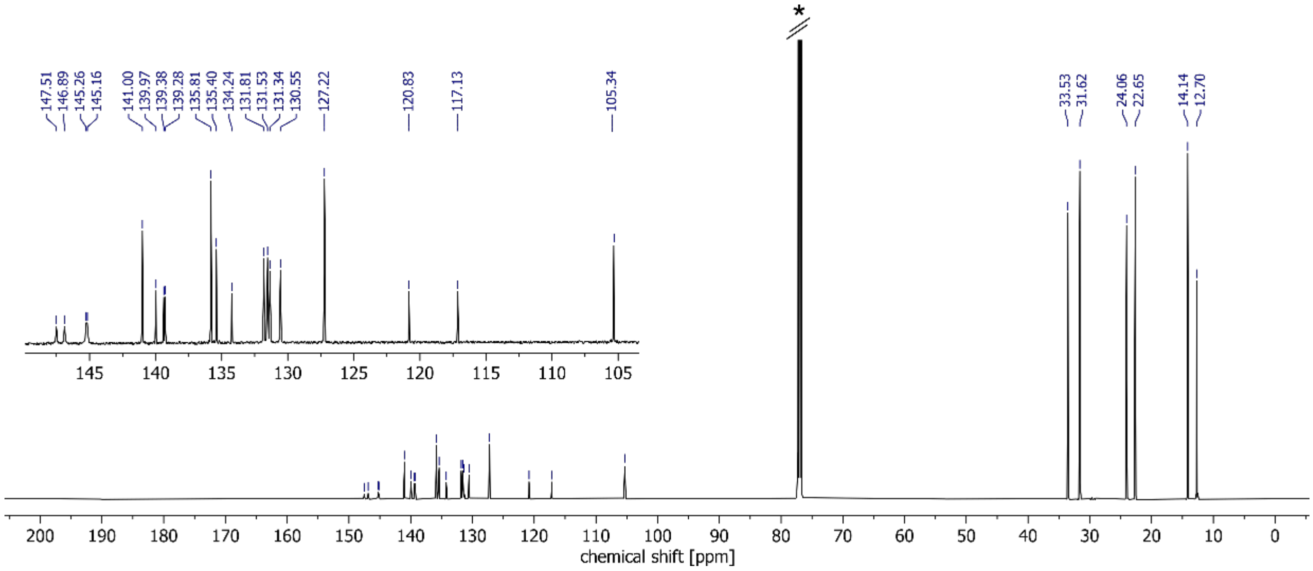

Figure S4. ^{13}C NMR spectrum of **S5**, $CDCl_3$, 151 MHz, 298 K.

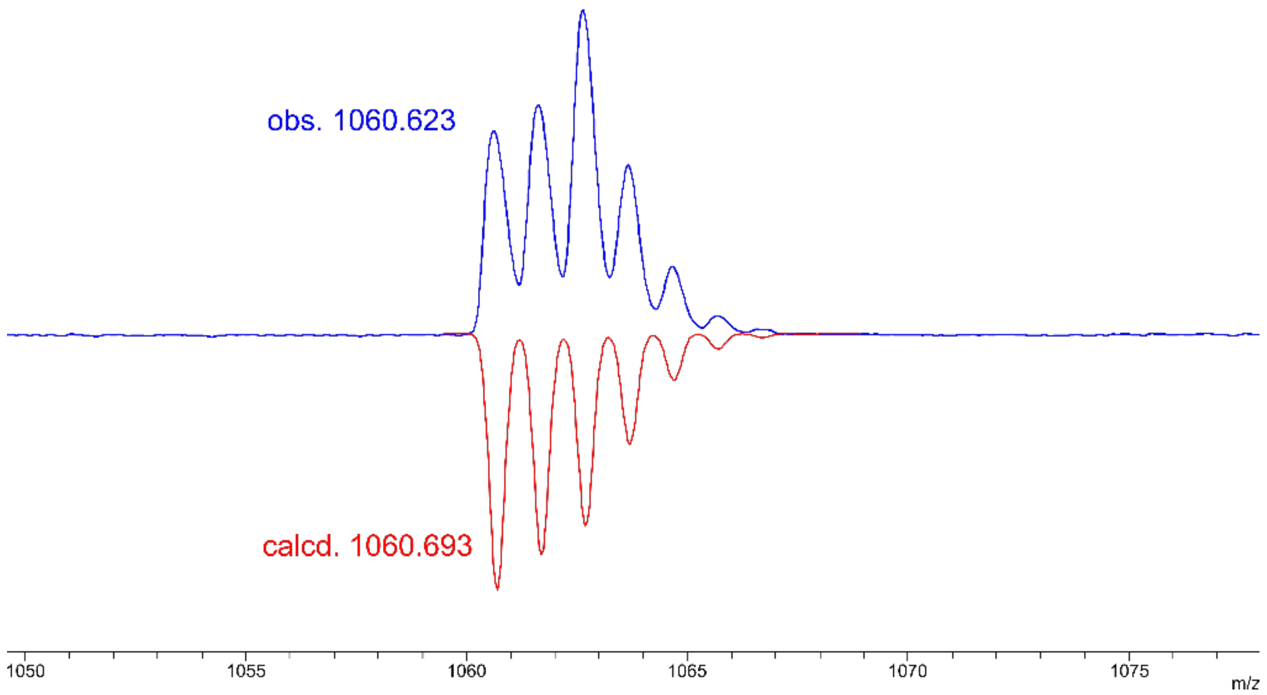

Figure S5. Selected region of a calculated (red) and recorded (blue) MALDI mass spectra of **S5**, DCTB matrix.

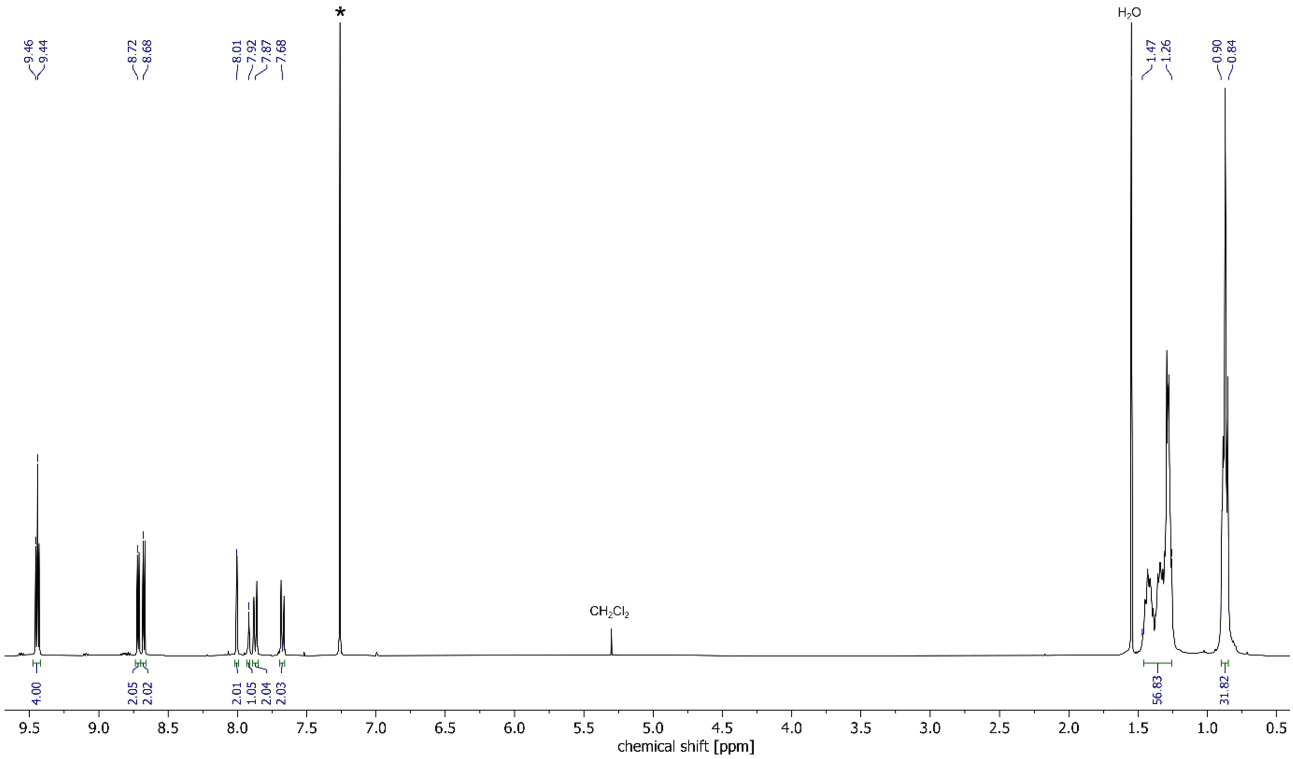

Figure S6. ^{1}H NMR spectrum of **4**, $CDCl_3$, 600 MHz, 298 K.

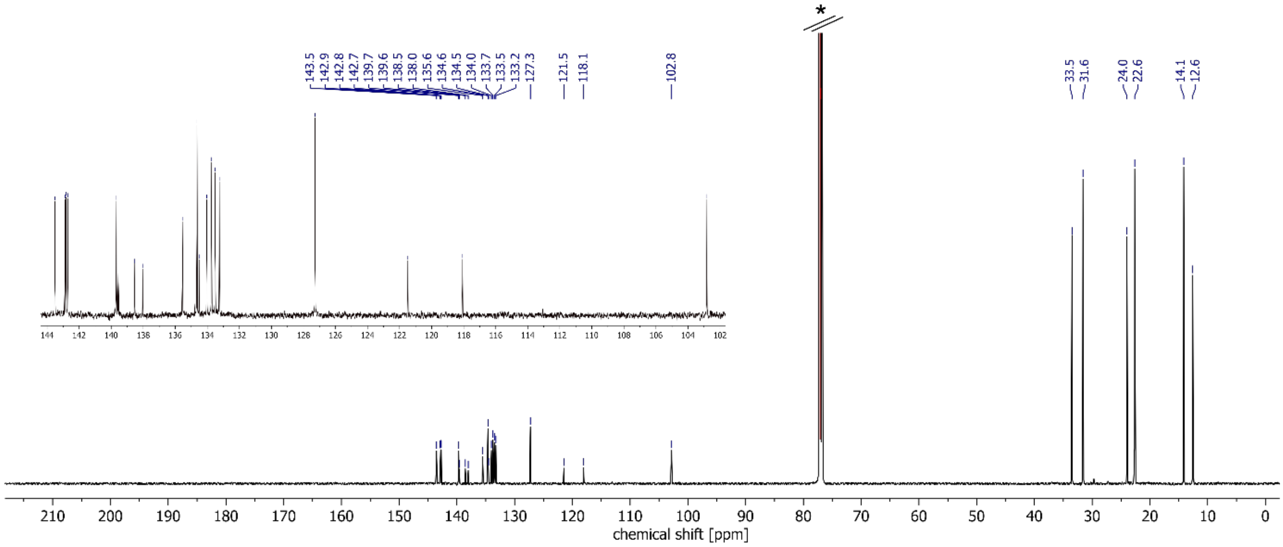

Figure S7. ^{13}C NMR spectrum of **4**, $CDCl_3$, 151 MHz, 298 K.

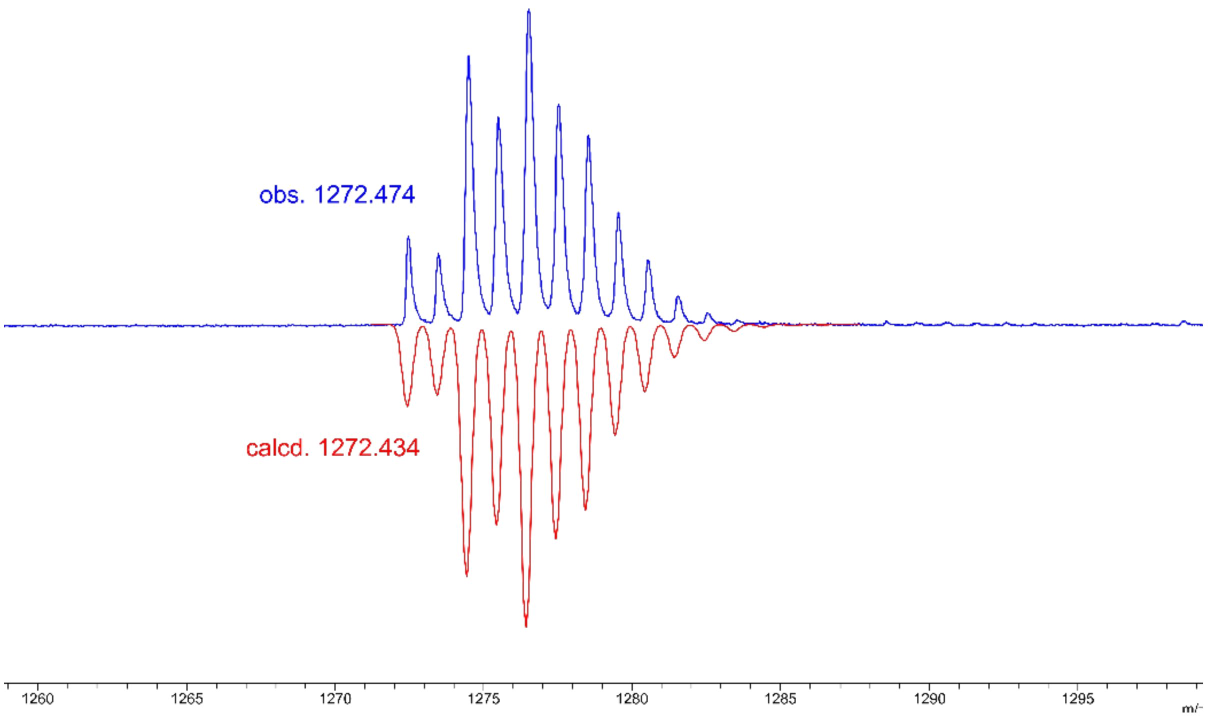

Figure S8. Selected region of a calculated (red) and recorded (blue) MALDI mass spectra of **4**, DCTB matrix.

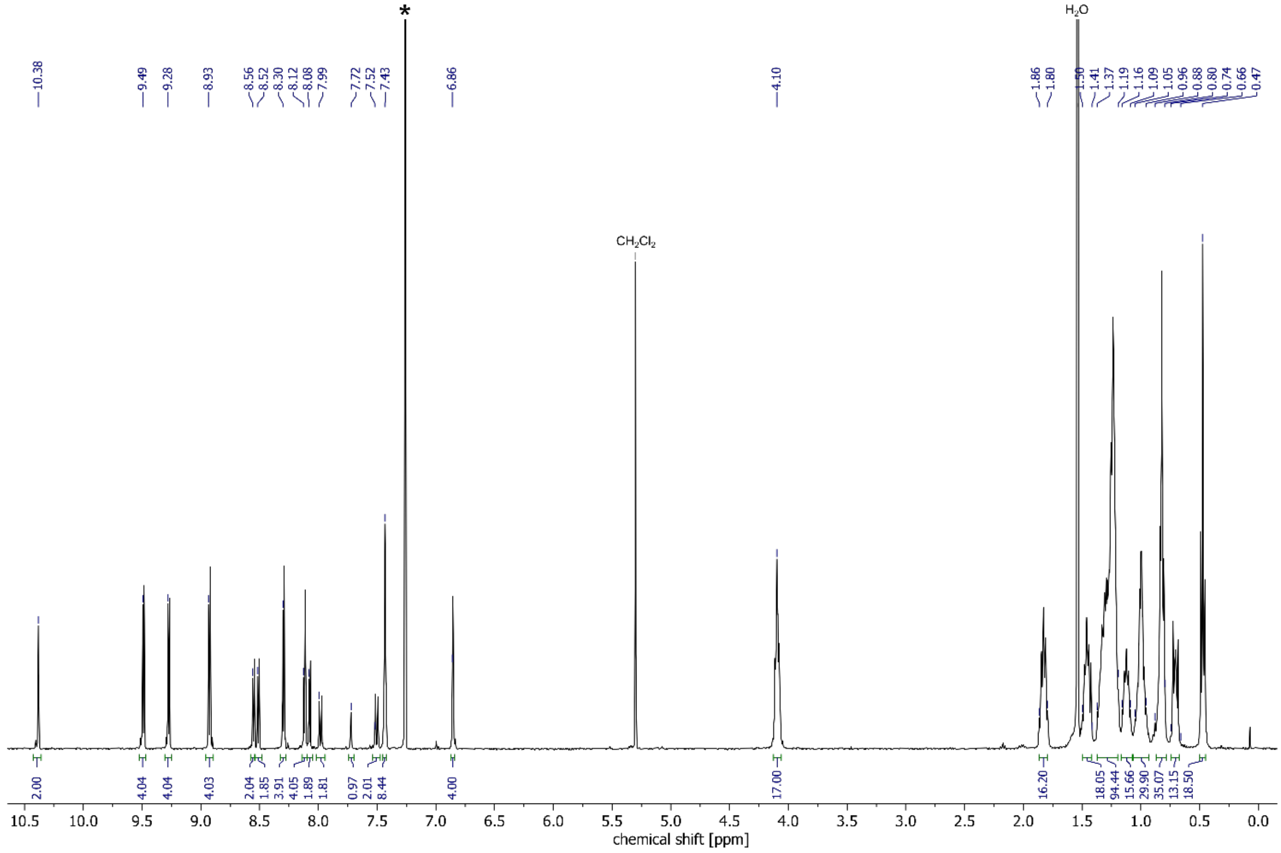

Figure S9. ^{1}H NMR spectrum of **S6**, $CDCl_3$, 600 MHz, 298 K.

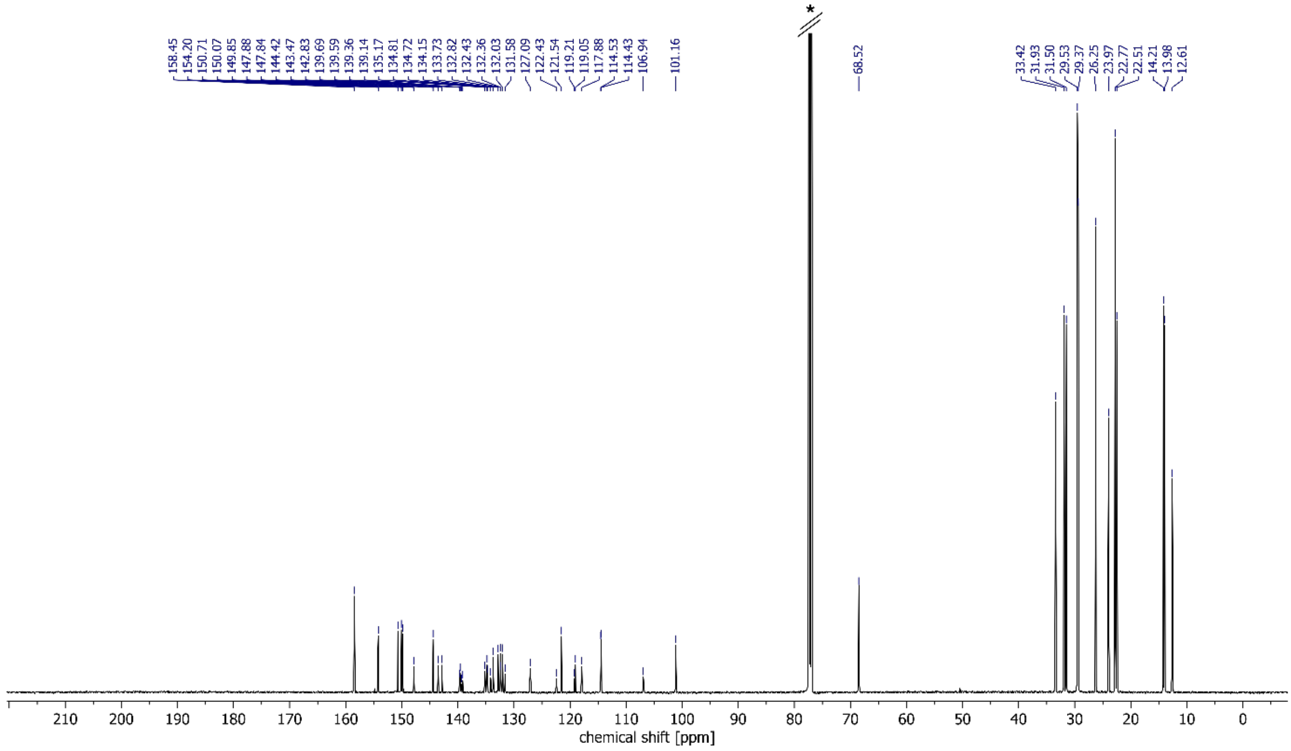

Figure S10. ^{13}C NMR spectrum of **S6**, $CDCl_3$, 151 MHz, 298 K.

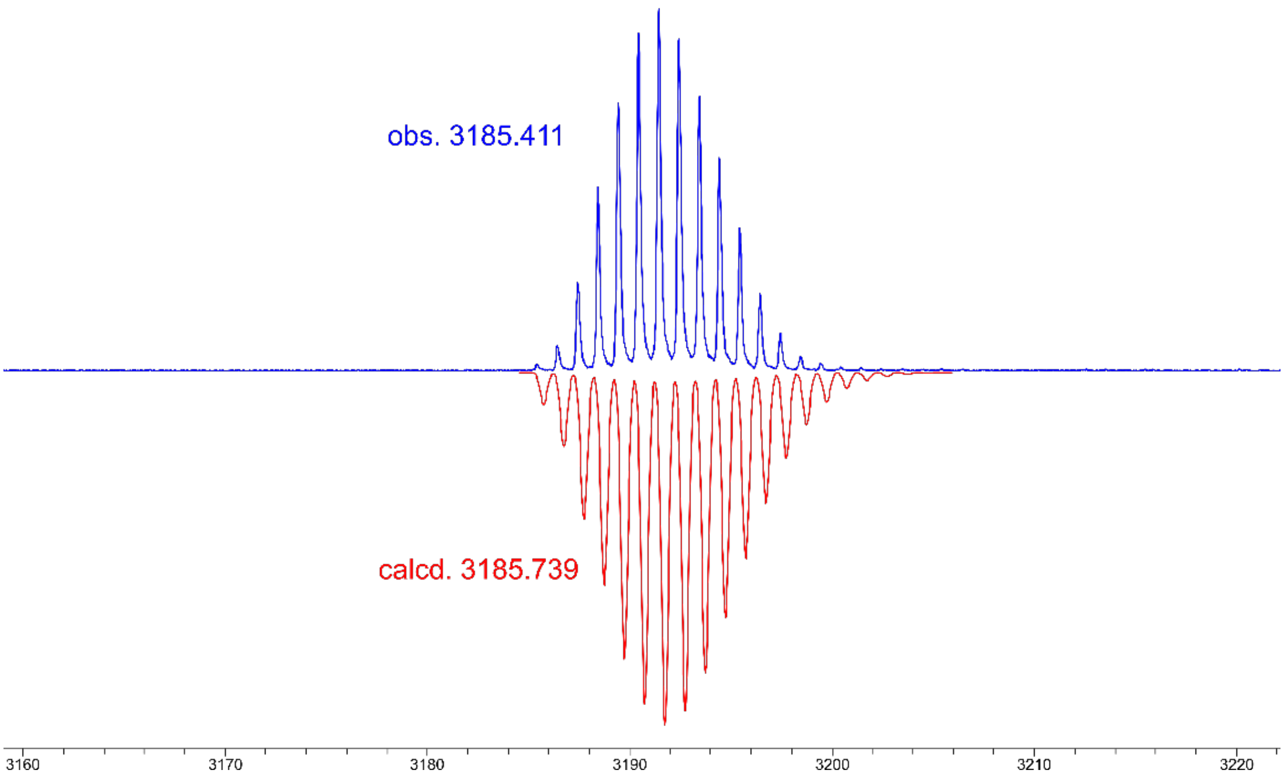

Figure S11. Selected region of calculated (red) and recorded (blue) MALDI mass spectra of **S6**, DCTB matrix.

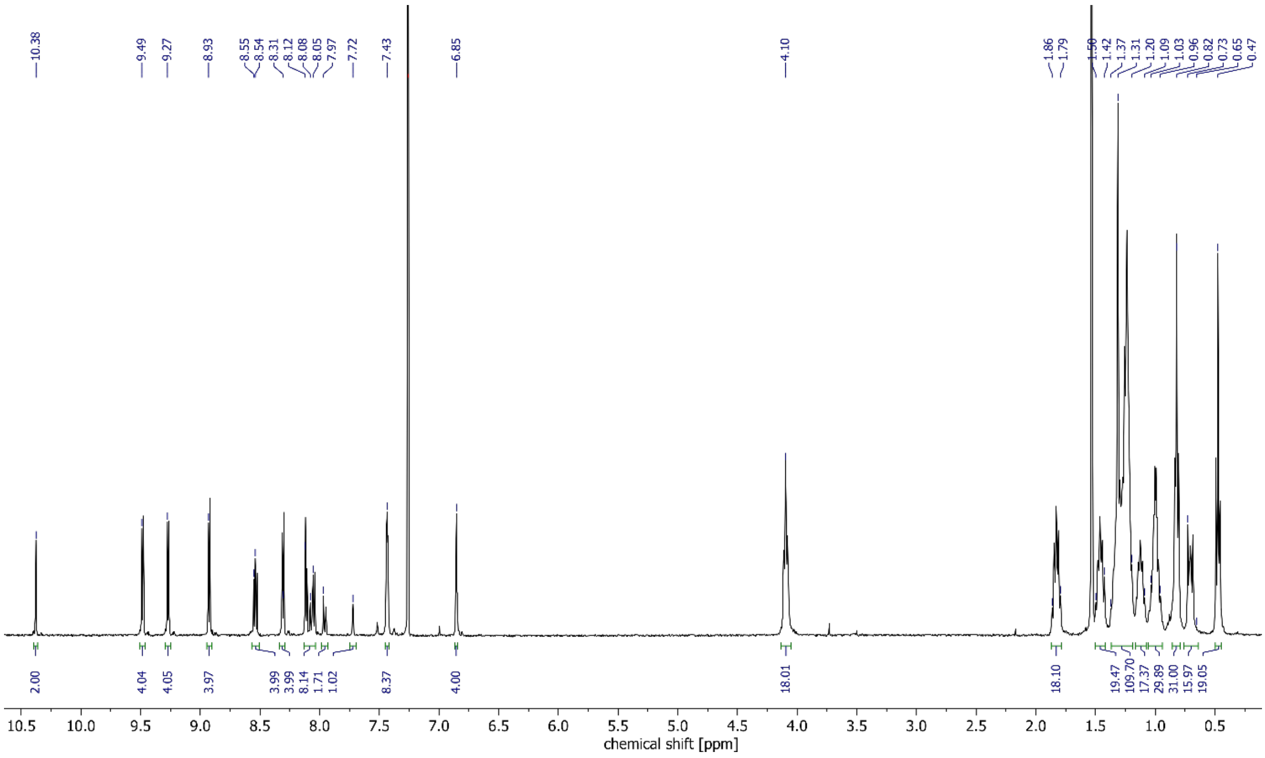

Figure S12. ^{1}H NMR spectrum of **6**, $CDCl_3$, 600 MHz, 298 K.

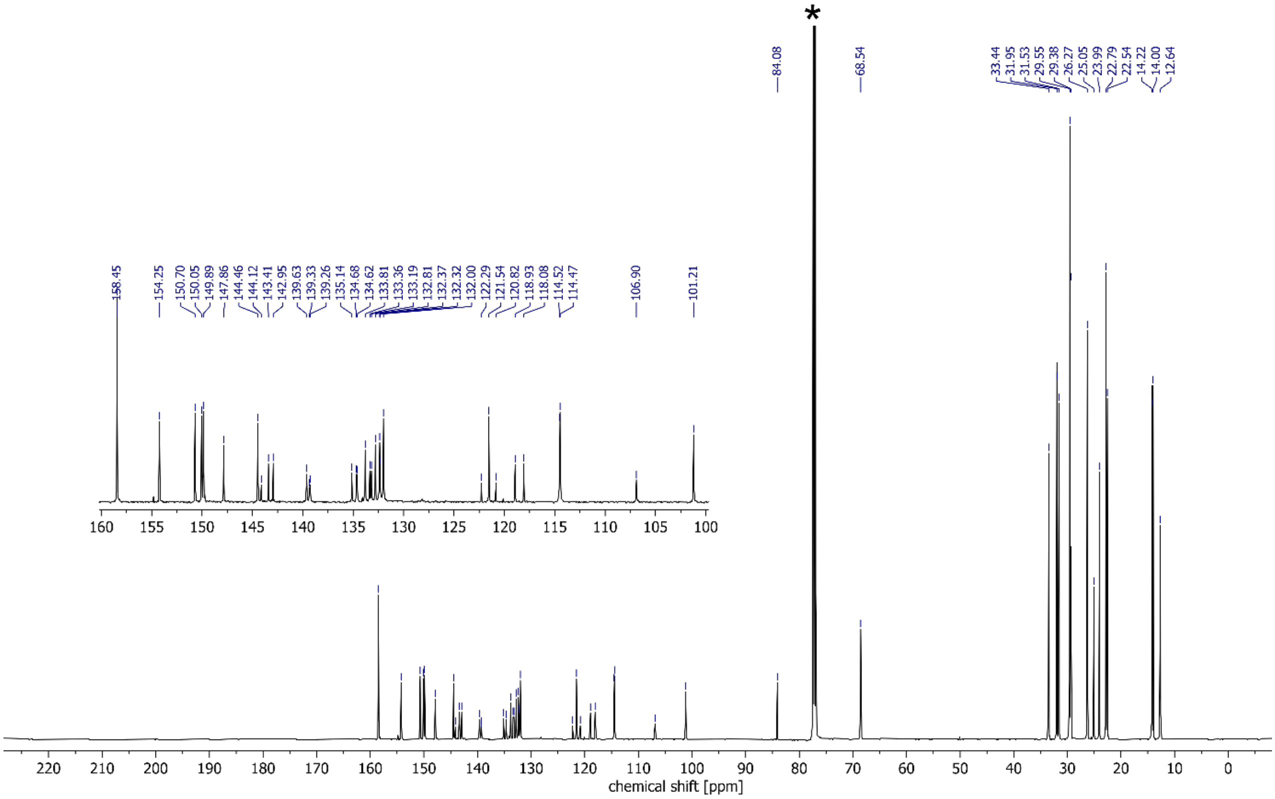

Figure S13. ^{13}C NMR spectrum of **6**, $CDCl_3$, 151 MHz, 298 K.

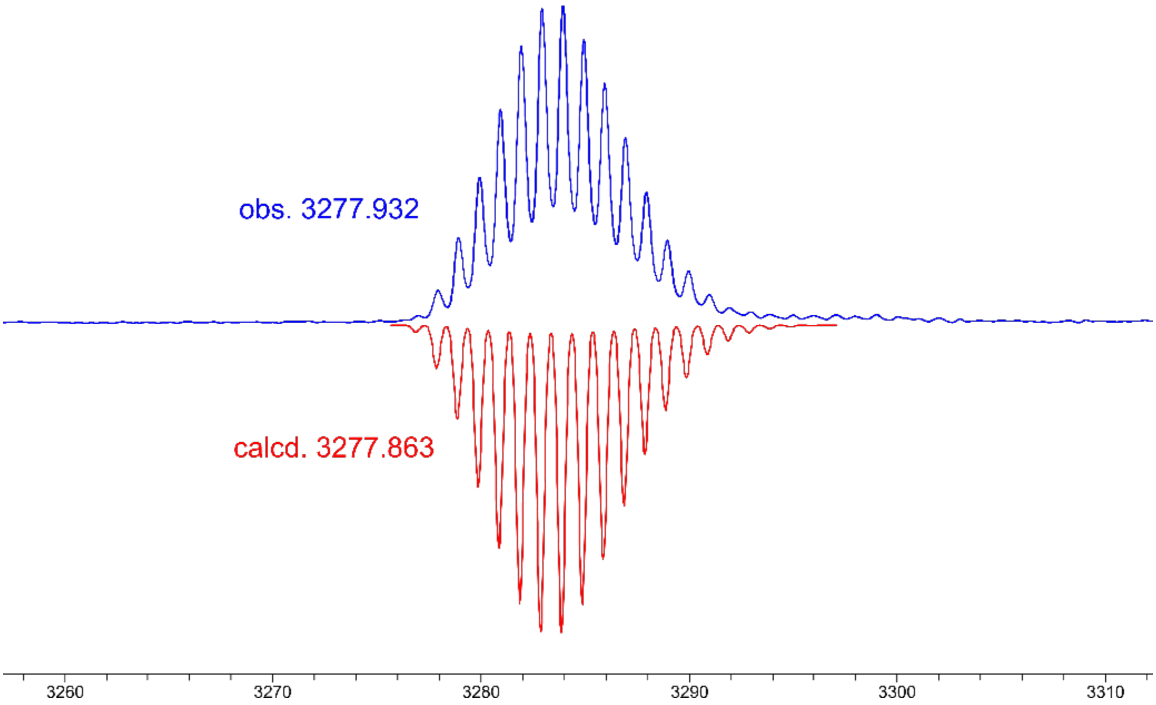

Figure S14. Selected region of calculated (red) and recorded (blue) MALDI mass spectra of **6**, DCTB matrix.

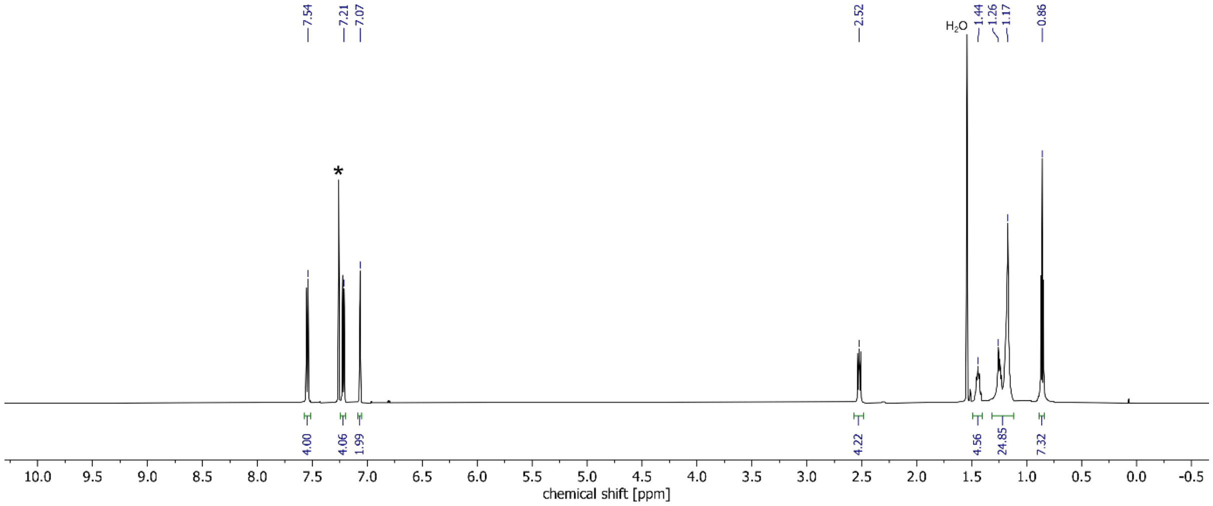

Figure S15. ^{1}H NMR spectrum of **S2**, $CDCl_3$, 600 MHz, 298 K.

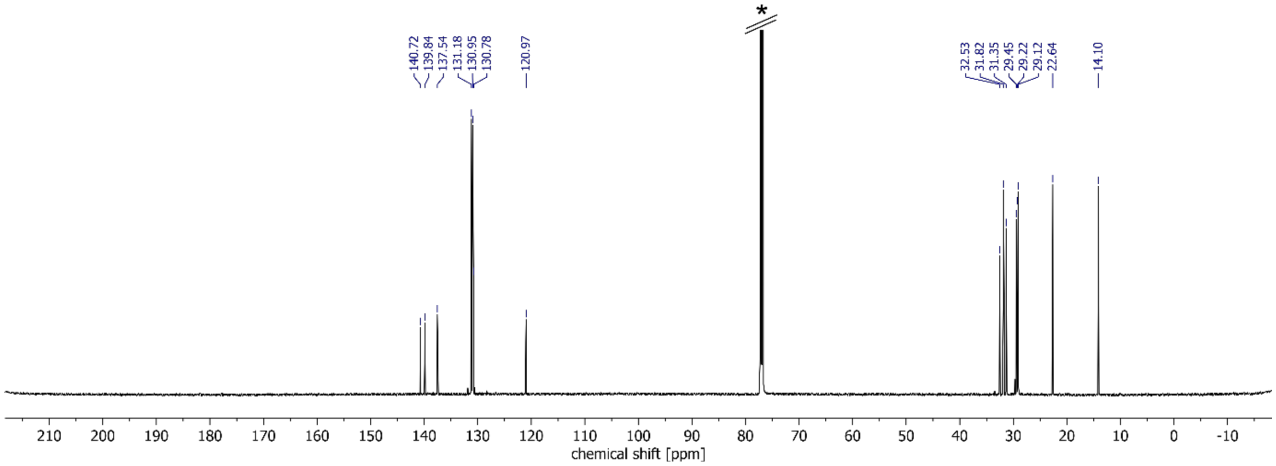

Figure S16. ^{13}C NMR spectrum of **S2**, $CDCl_3$, 151 MHz, 298 K.

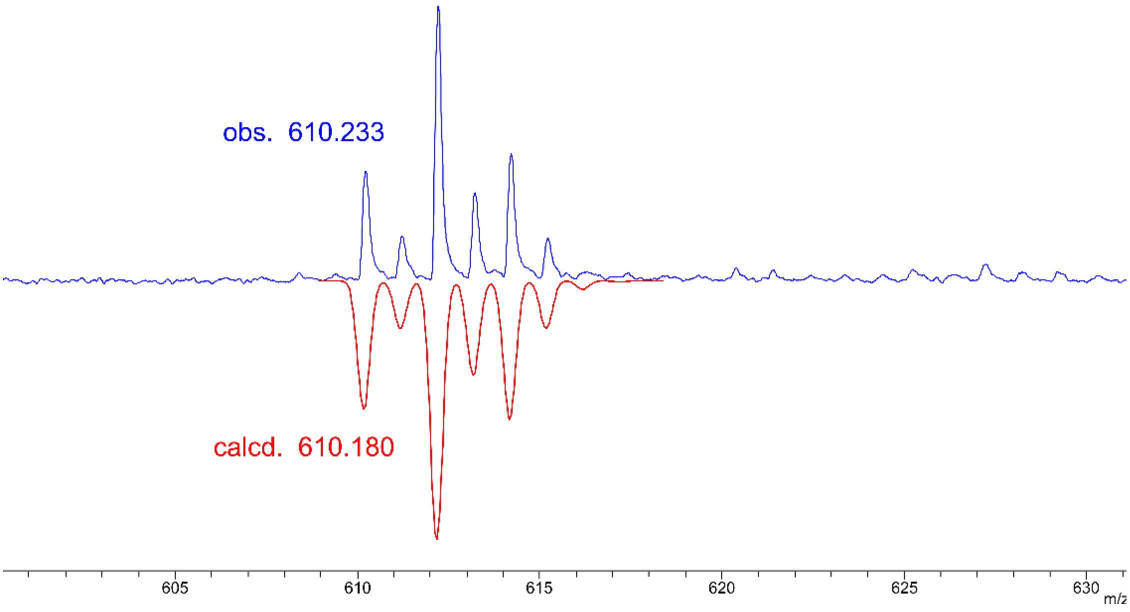

Figure S17. Selected region of a calculated (red) and recorded (blue) MALDI mass spectra of **S2**, DCTB matrix.

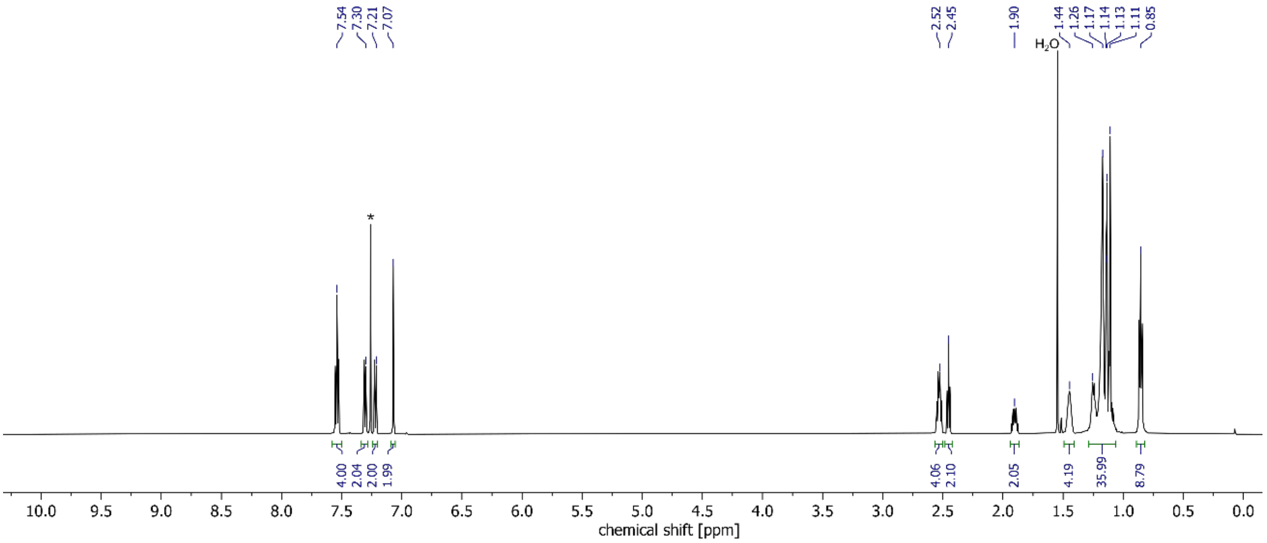

Figure S18. ^{1}H NMR spectrum of **S3**, $CDCl_3$, 600 MHz, 298 K.

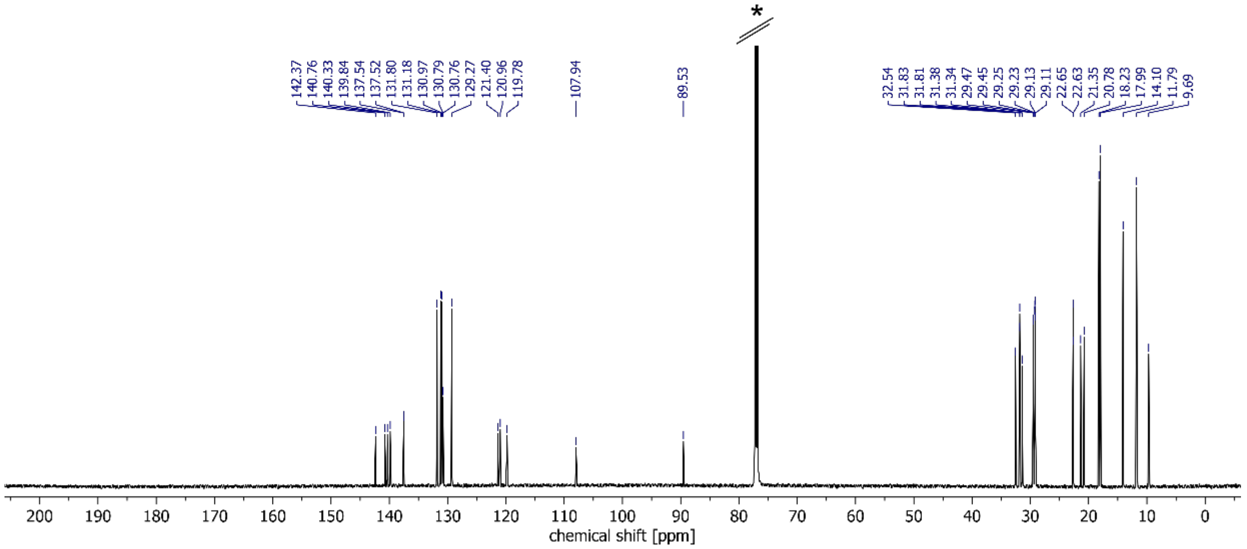

Figure S19. ^{13}C NMR spectrum of **S3**, $CDCl_3$, 151 MHz, 298 K.

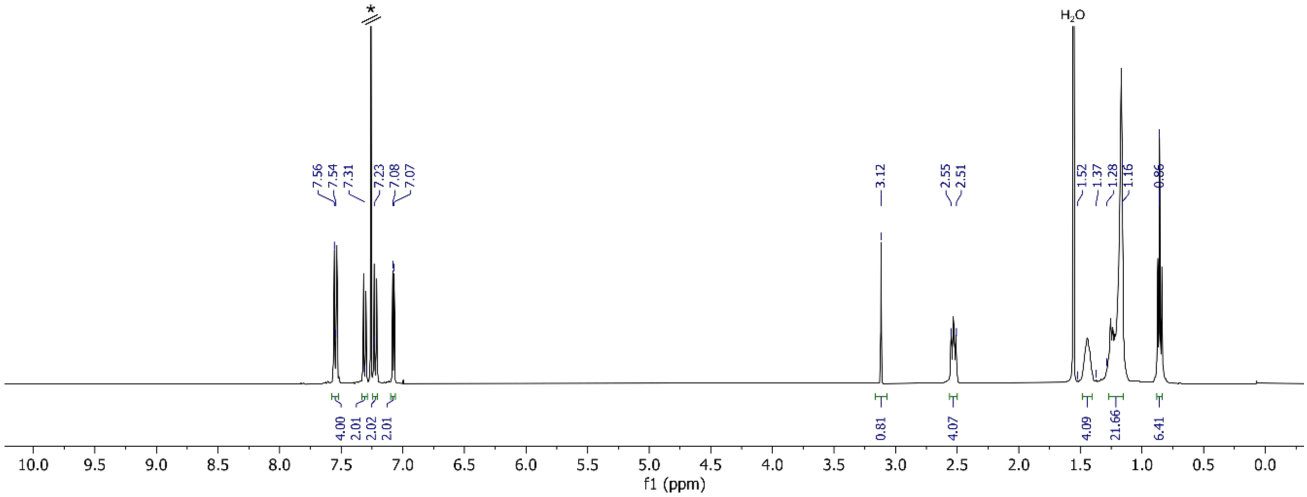

Figure S20. ^{1}H NMR spectrum of **S4**, $CDCl_3$, 600 MHz, 298 K.

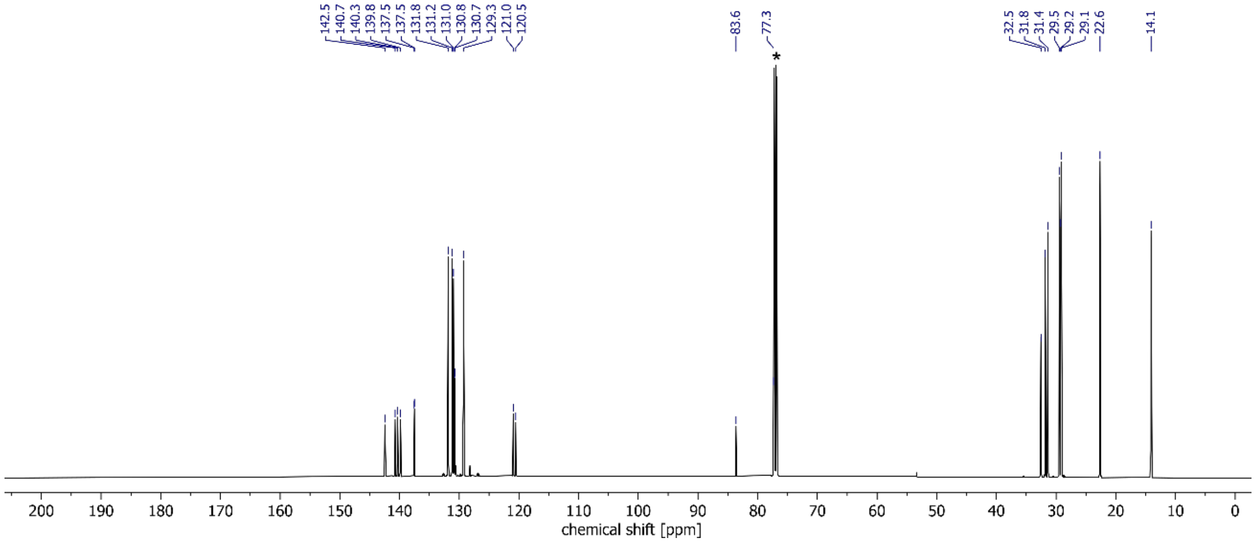

Figure S21. ^{13}C NMR spectrum of **S4**, $CDCl_3$, 151 MHz, 298 K.

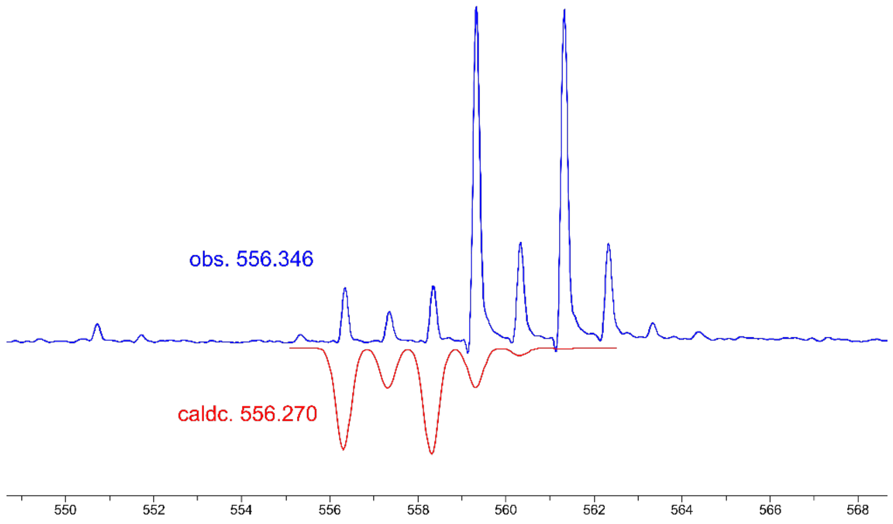

Figure S22. Selected region of calculated (red) and recorded (blue) MALDI mass spectra of **S4**, DCTB matrix.

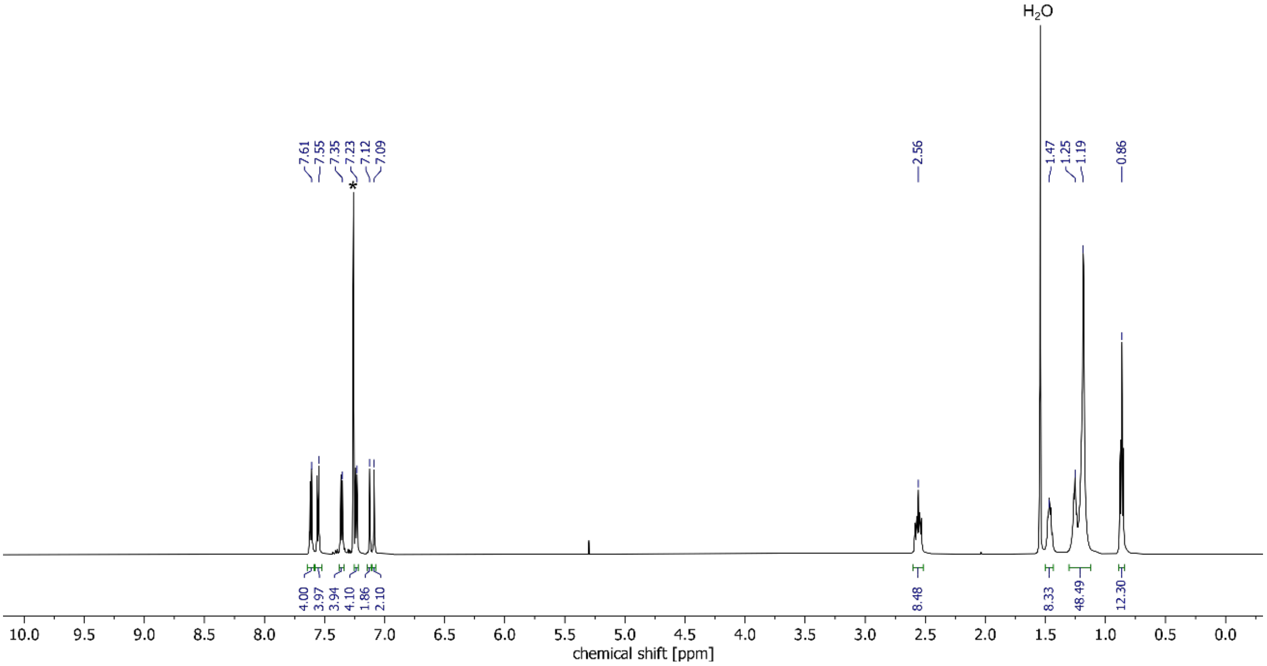

Figure S23. ^{1}H NMR spectrum of **7**, $CDCl_3$, 600 MHz, 298 K.

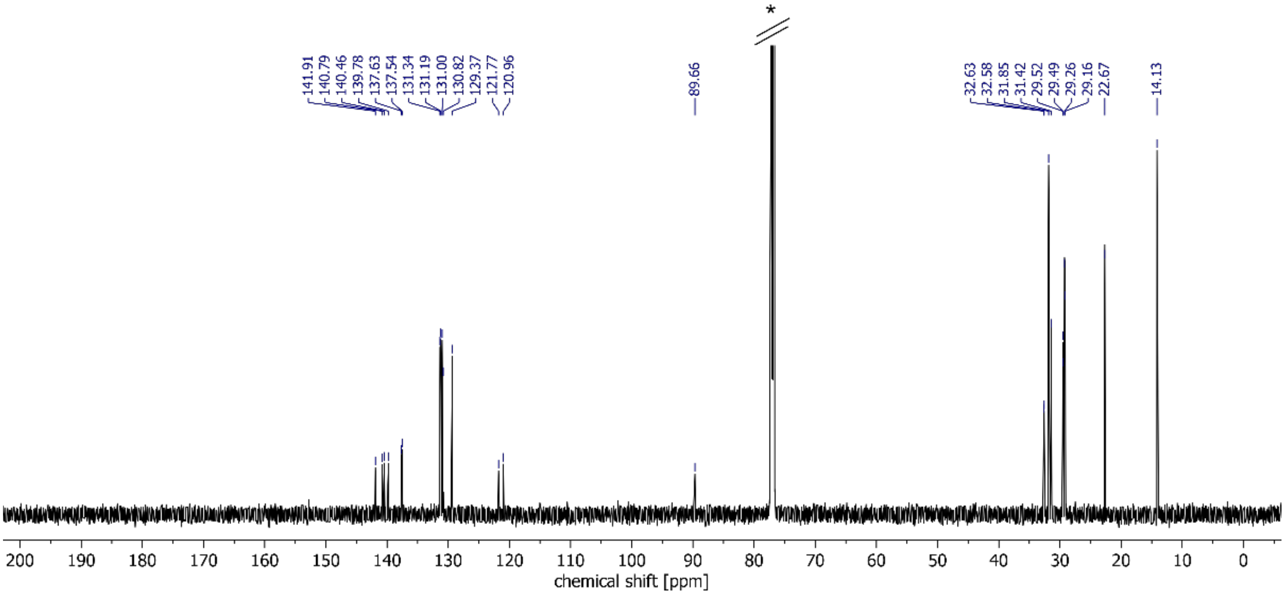

Figure S24. ^{13}C NMR spectrum of **7**, $CDCl_3$, 151 MHz, 298 K.

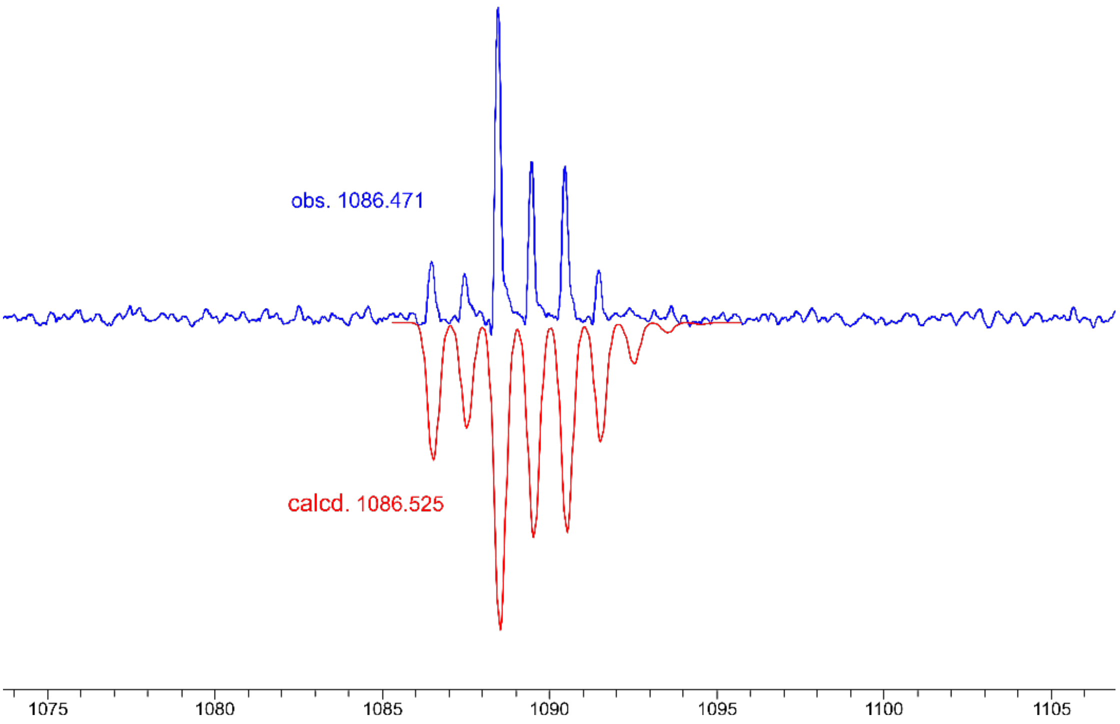

Figure S25. Selected region of calculated (red) and recorded (blue) MALDI mass spectra of **7**, DCTB matrix.

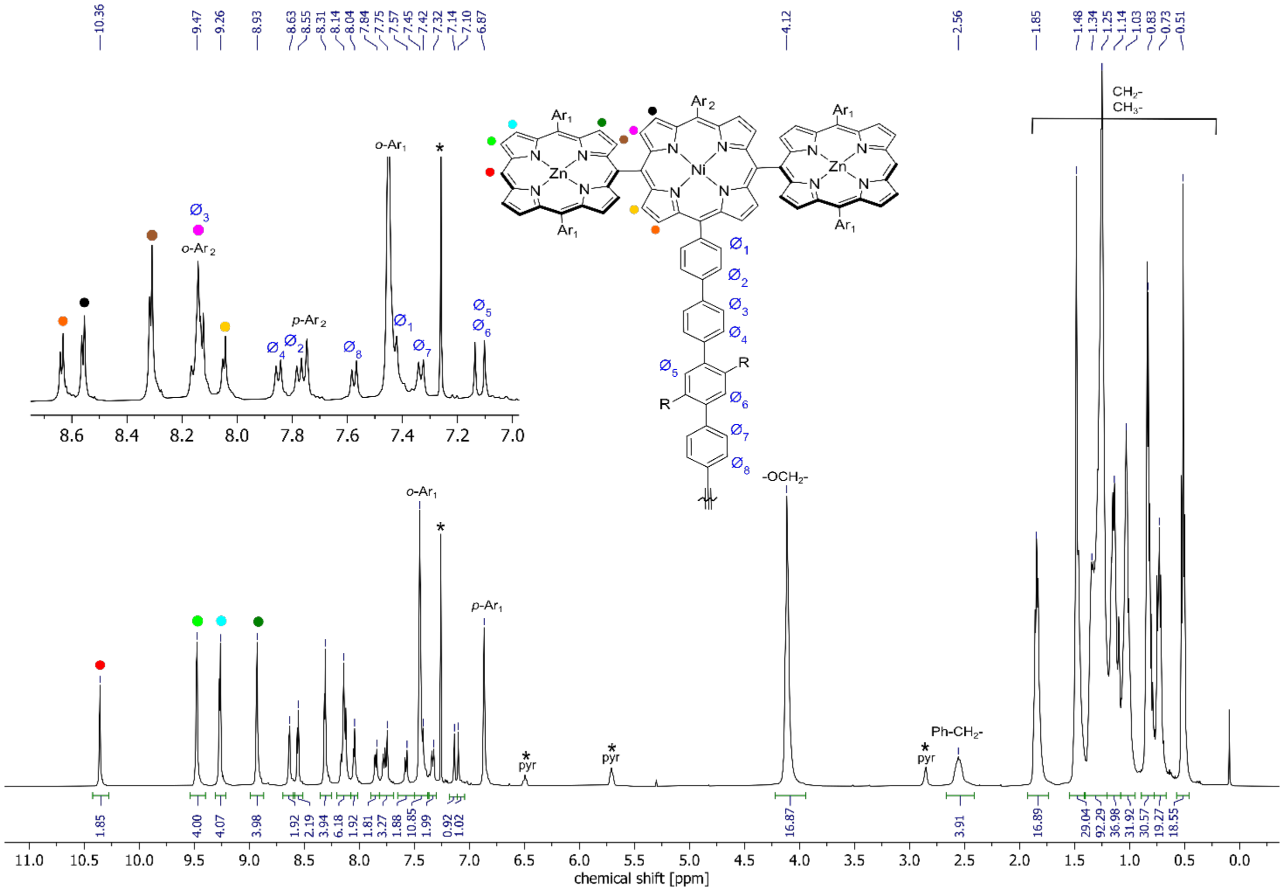

Figure S26. ^{1}H NMR spectrum of **3**, $CDCl_3$, 600 MHz, 298 K.

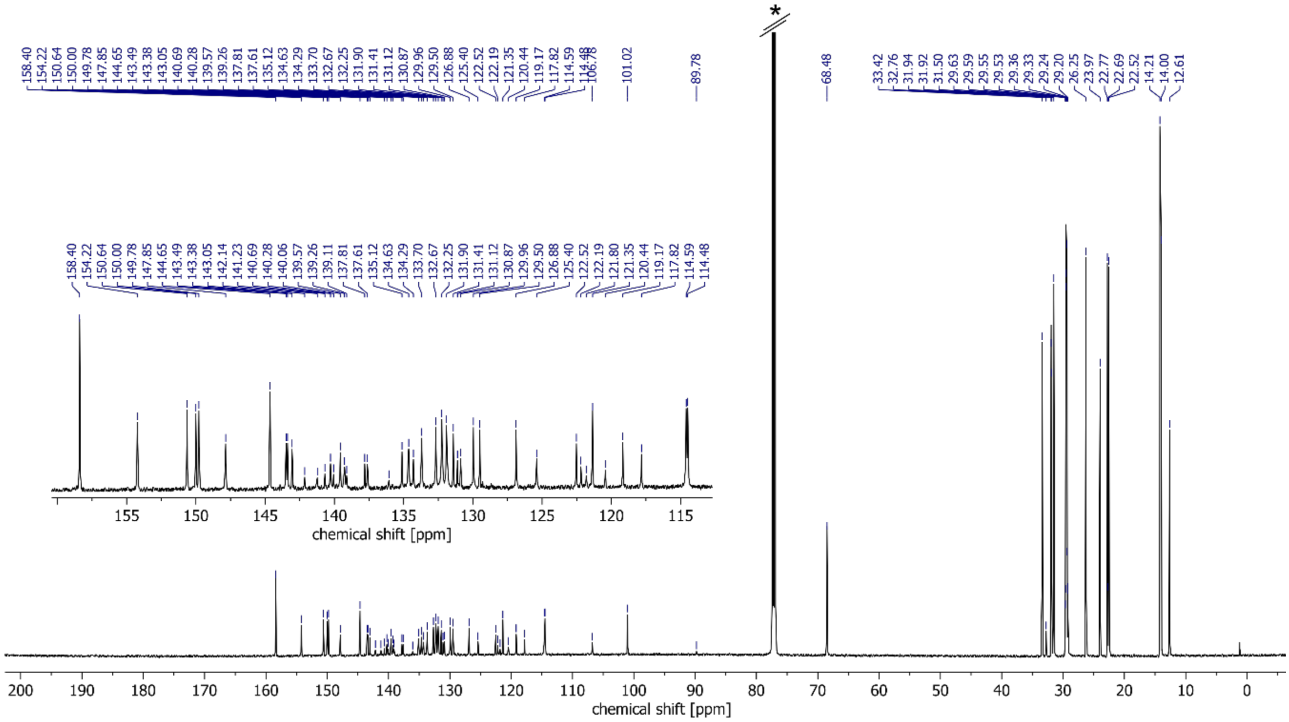

Figure S27. ^{13}C NMR spectrum of **3**, $CDCl_3$, 151 MHz, 298 K.

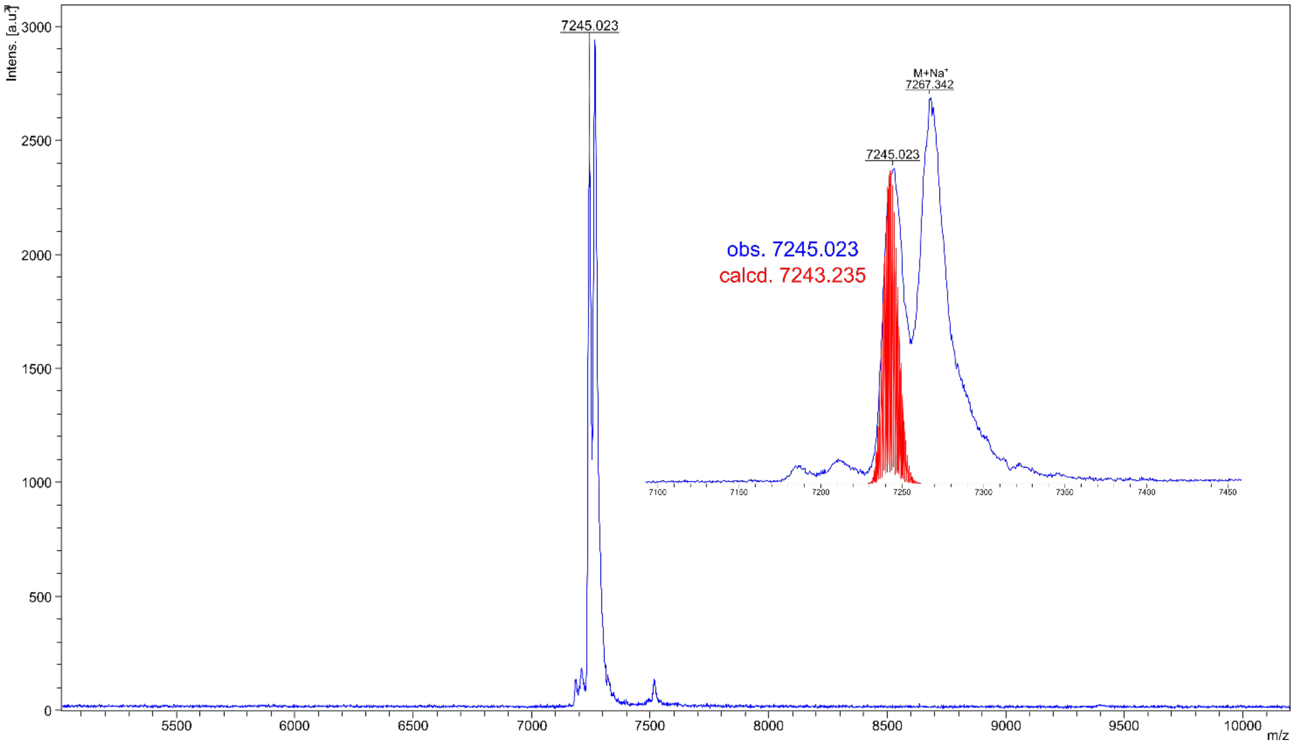

Figure S28. Selected region of calculated (red) and recorded (blue) MALDI mass spectra of **3**, DCTB matrix.

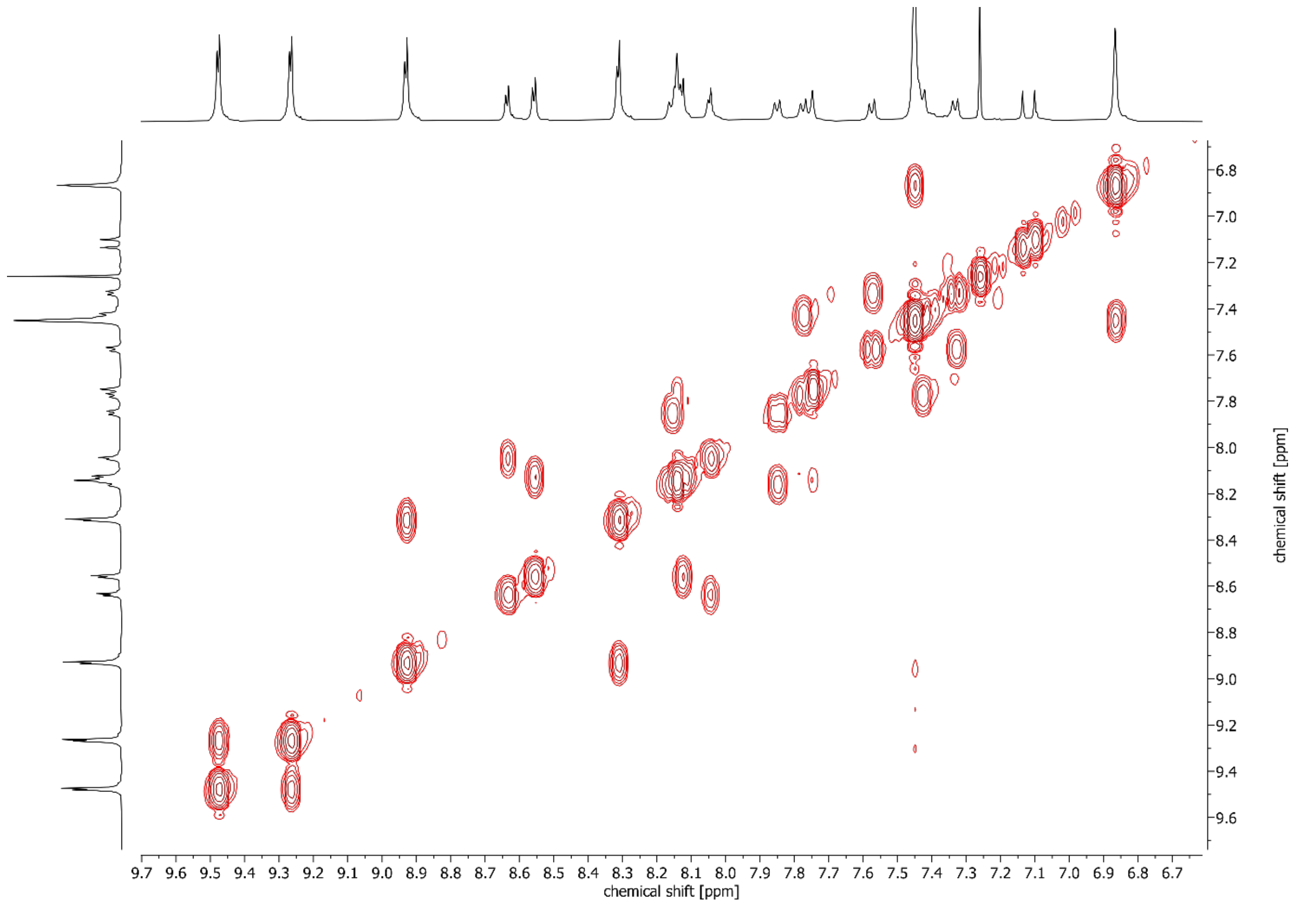

Figure S29. Selected aromatic region of a ^{1}H-^{1}H COSY NMR spectrum of **3**, $CDCl_3$, 600 MHz, 298 K.

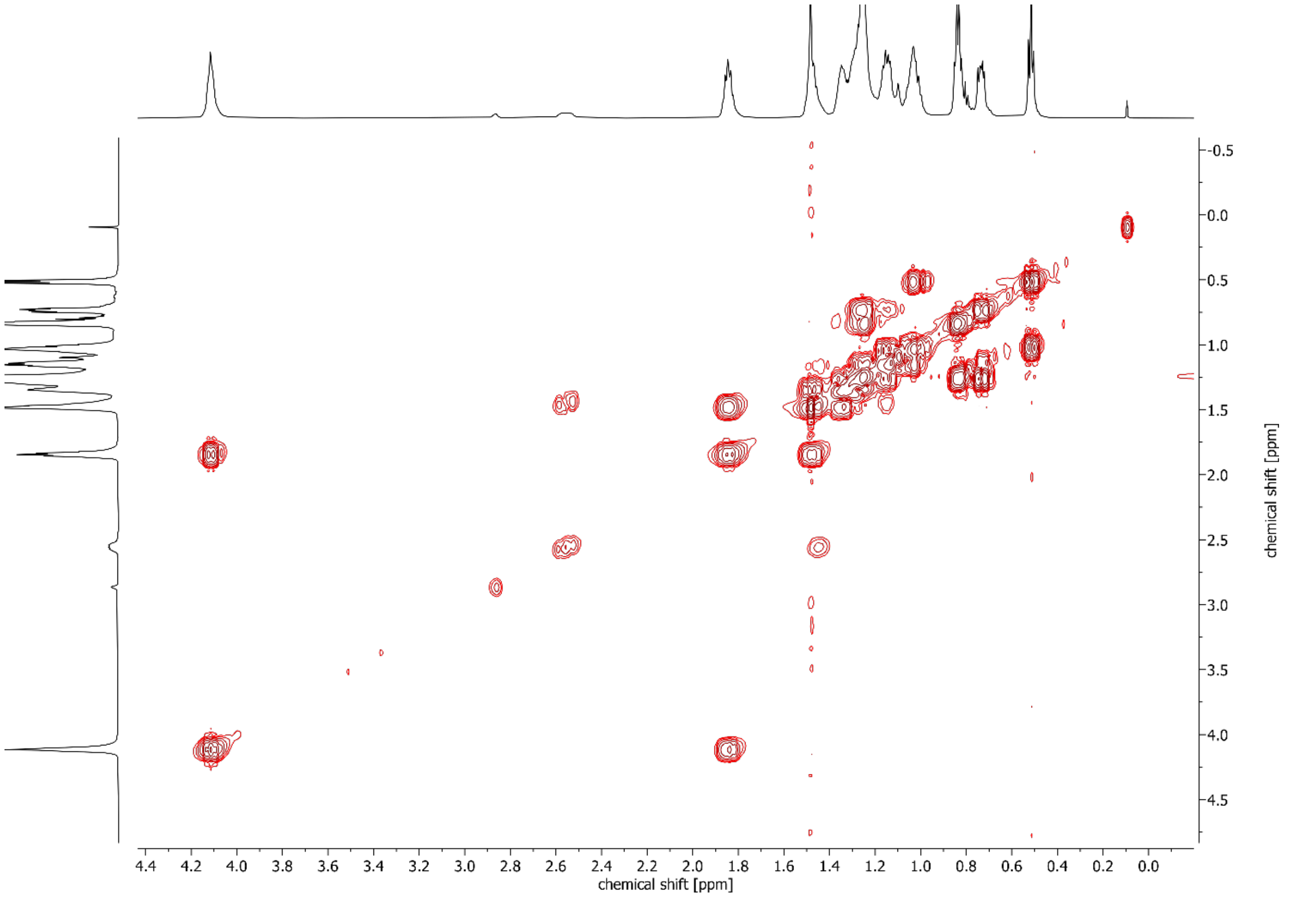

Figure S30. Selected alkyl region of a ^{1}H-^{1}H COSY NMR spectrum of **3**, $CDCl_3$, 600 MHz, 298 K.

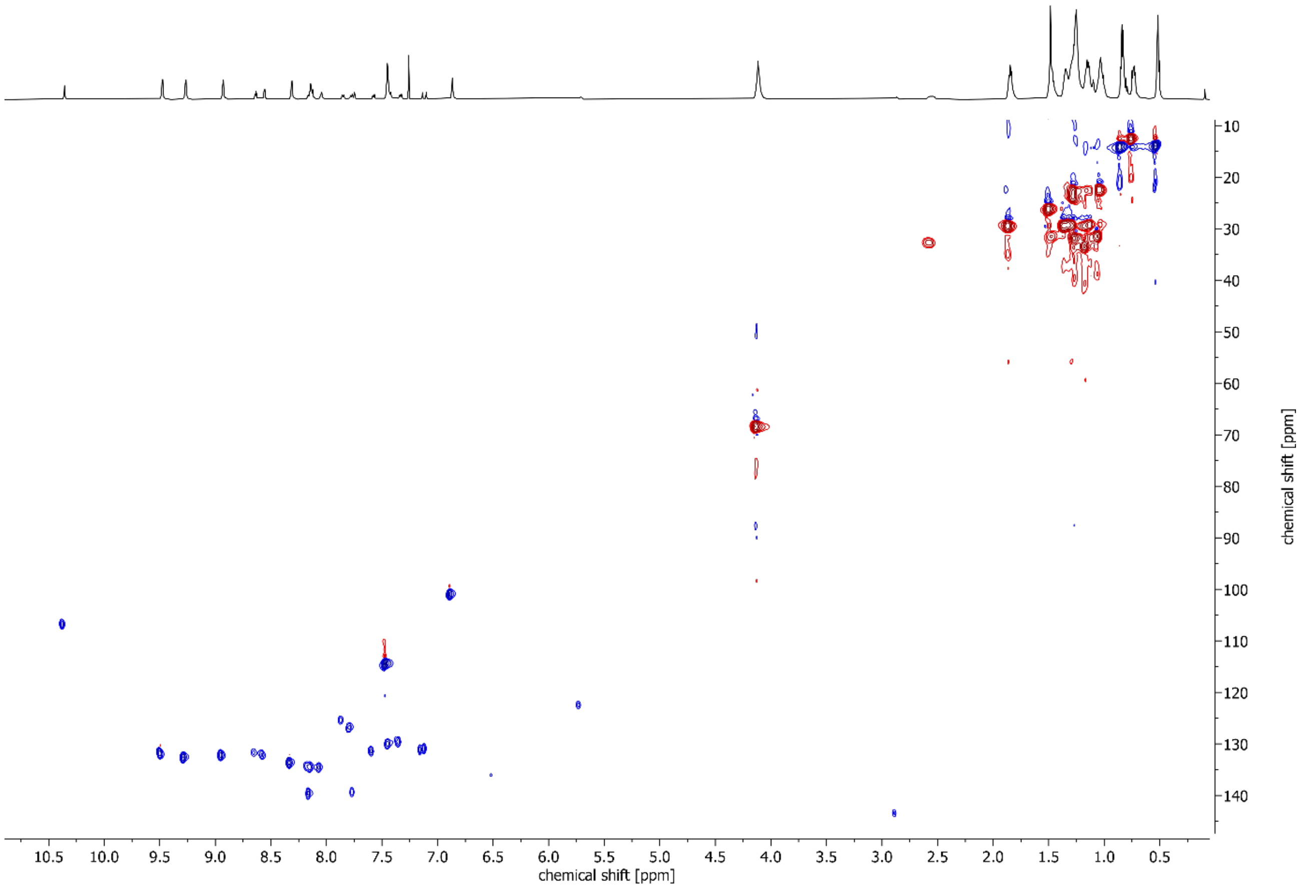

Figure S31. ^{1}H-^{13}C HSQC NMR spectrum of **3**, $CDCl_3$, 600 MHz, 298 K.

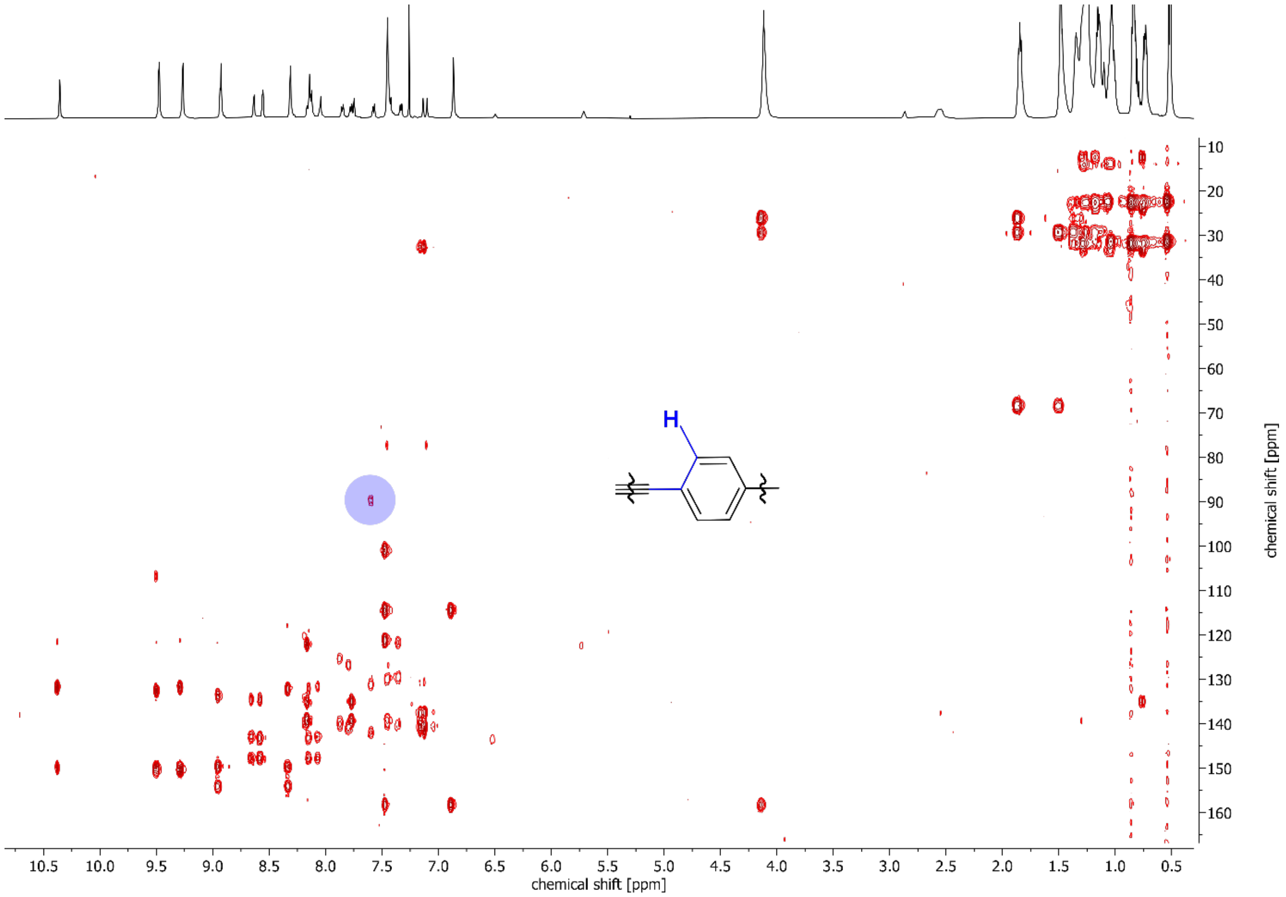

Figure S32. ^{1}H-^{13}C HMBC NMR spectrum of **3**, $CDCl_3$, 600 MHz, 298 K. Correlation between acetylene carbon and neighboring phenyl proton is marked in blue.

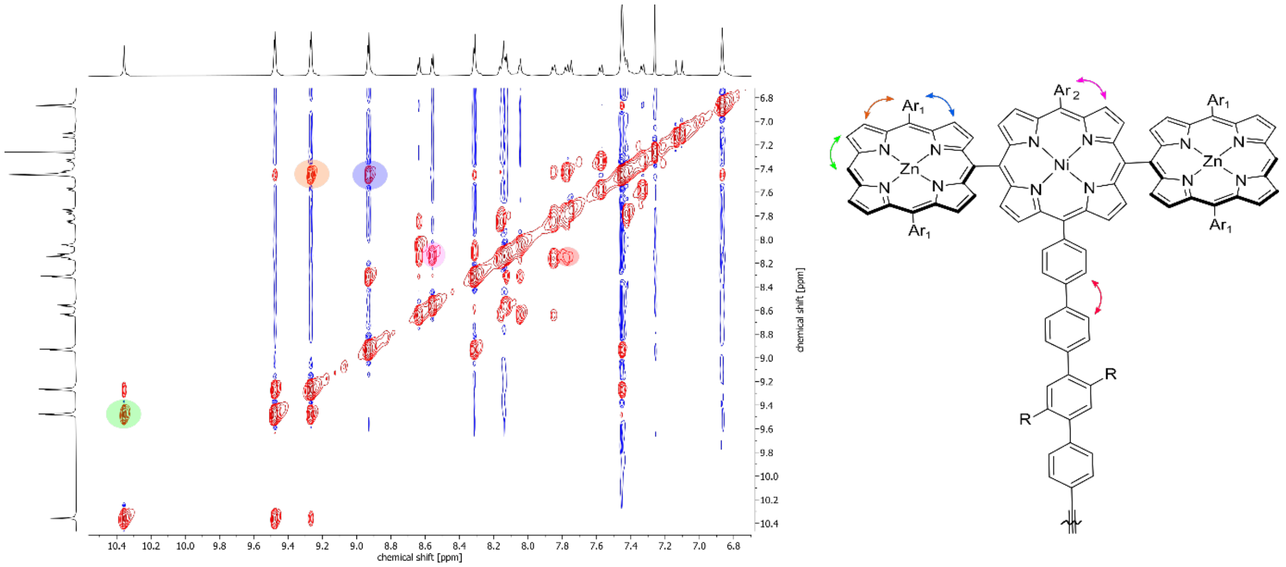

Figure S33. Selected aromatic region of a ^{1}H-^{1}H NOESY NMR spectrum of **3**, $CDCl_3$, 600 MHz, 298 K with assigned correlations crucial for signal assignment.

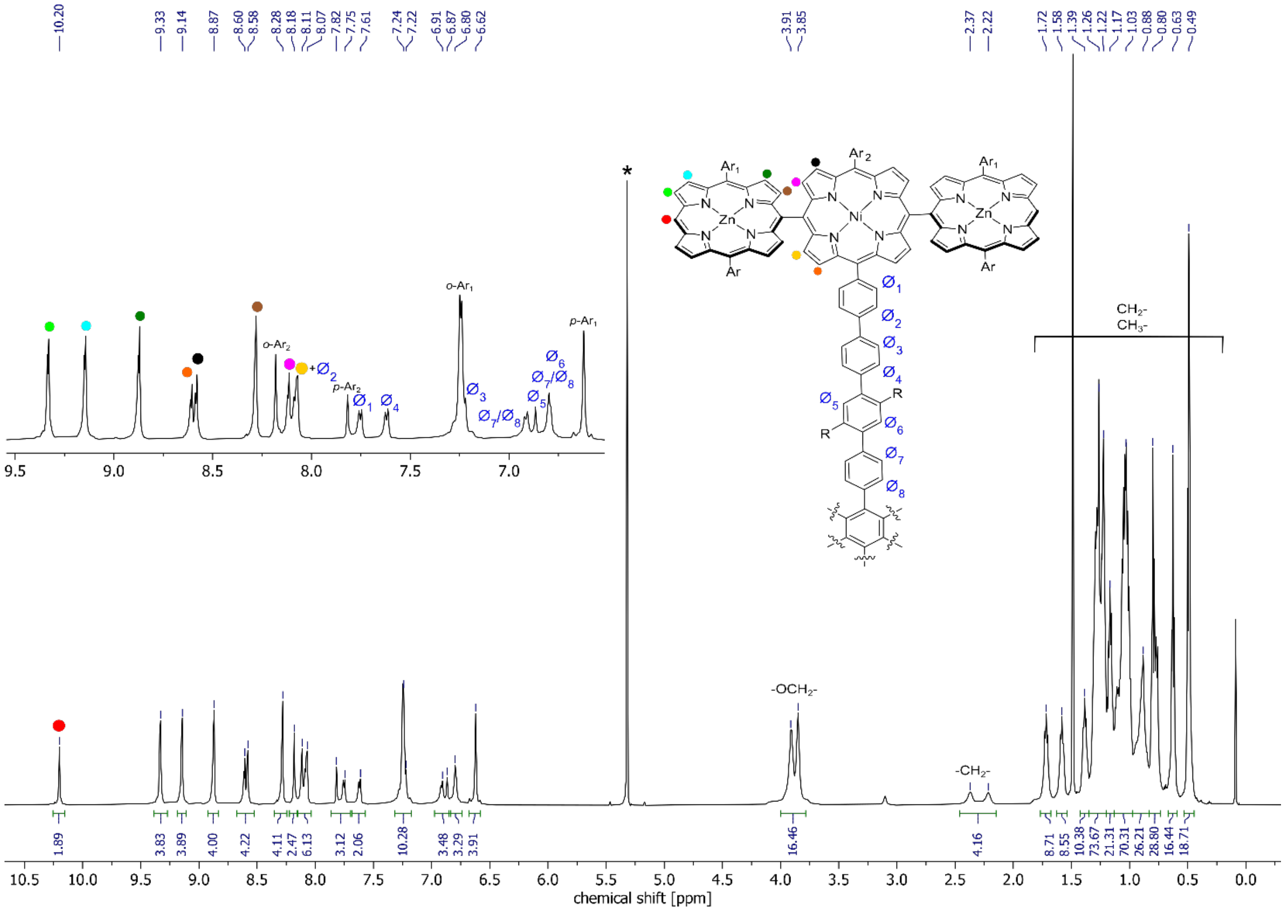

Figure S34. ^{1}H NMR spectrum of **2**, CD_2Cl_2, 600 MHz, 298 K.

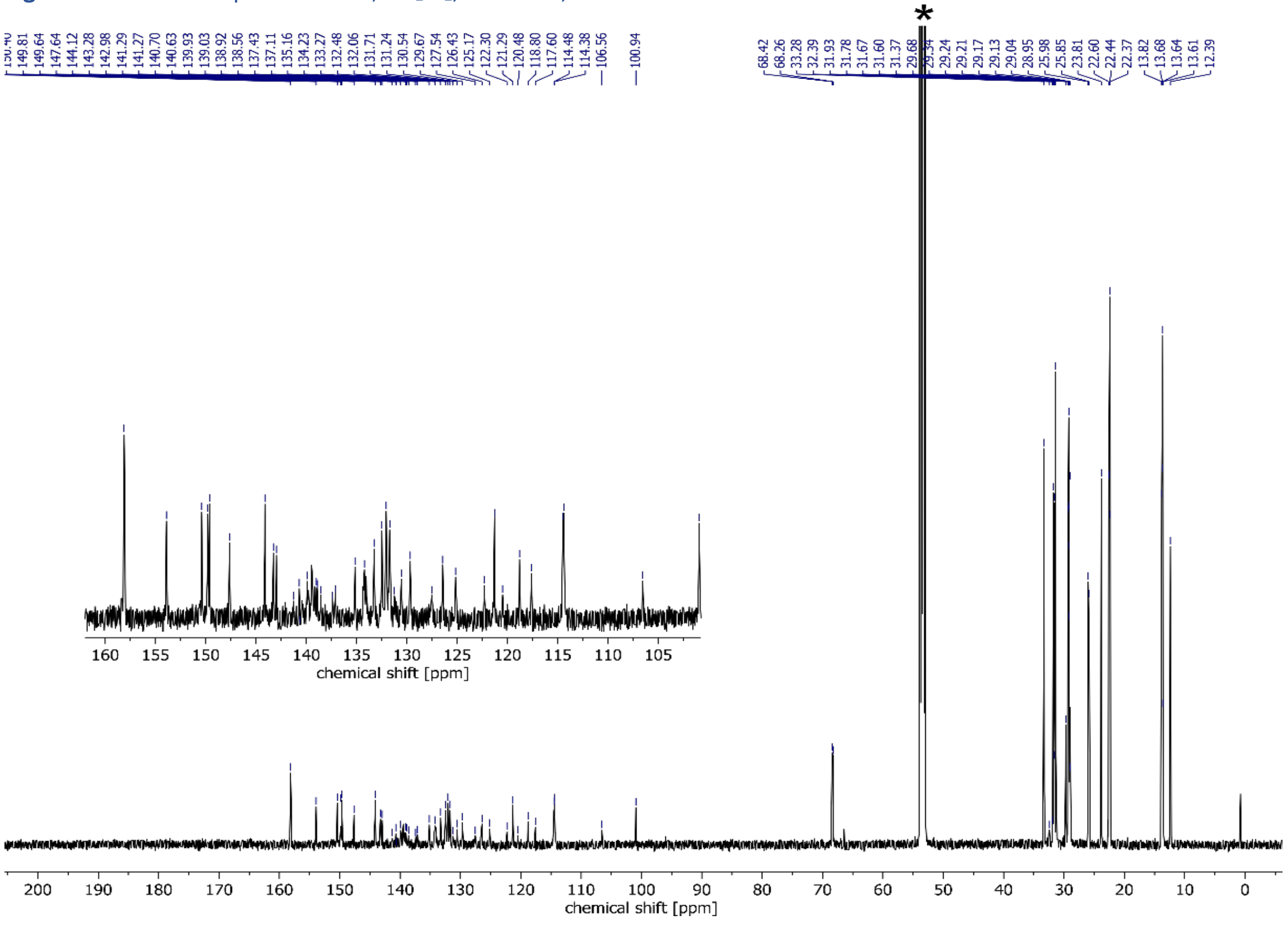

Figure S35. ^{13}C NMR spectrum of **2**, CD_2Cl_2, 151 MHz, 298 K.

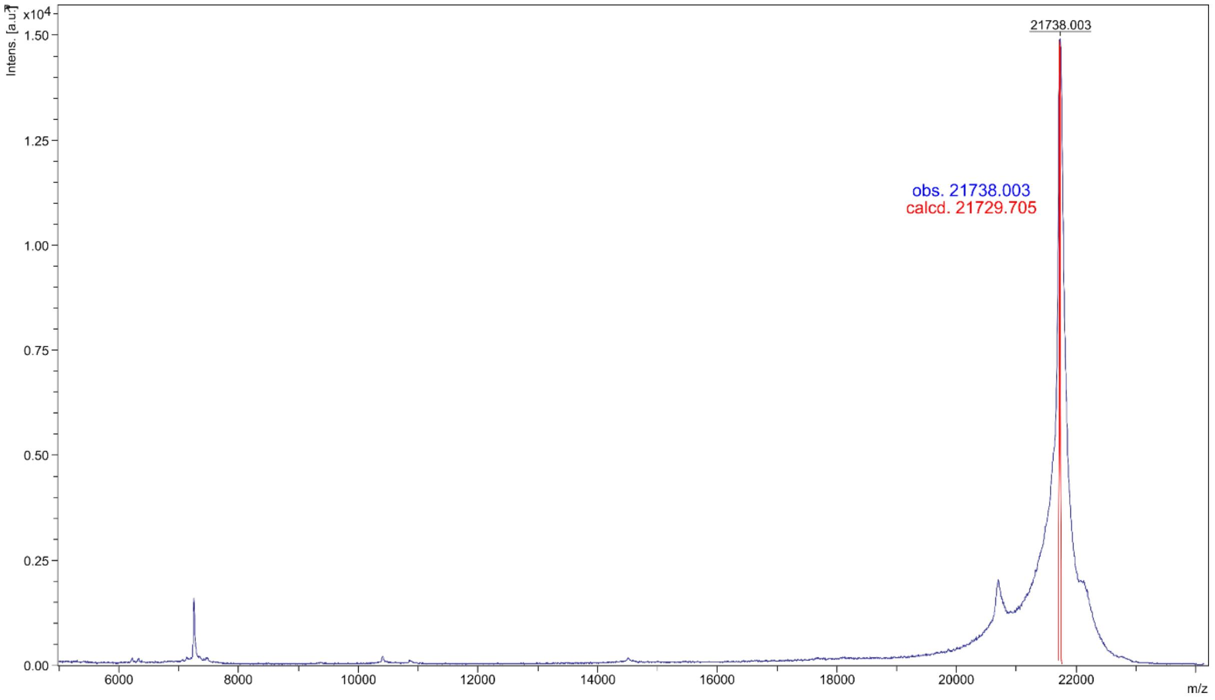

Figure S36. Calculated (red) and recorded (blue) MALDI mass spectra of **2**, DCTB matrix.

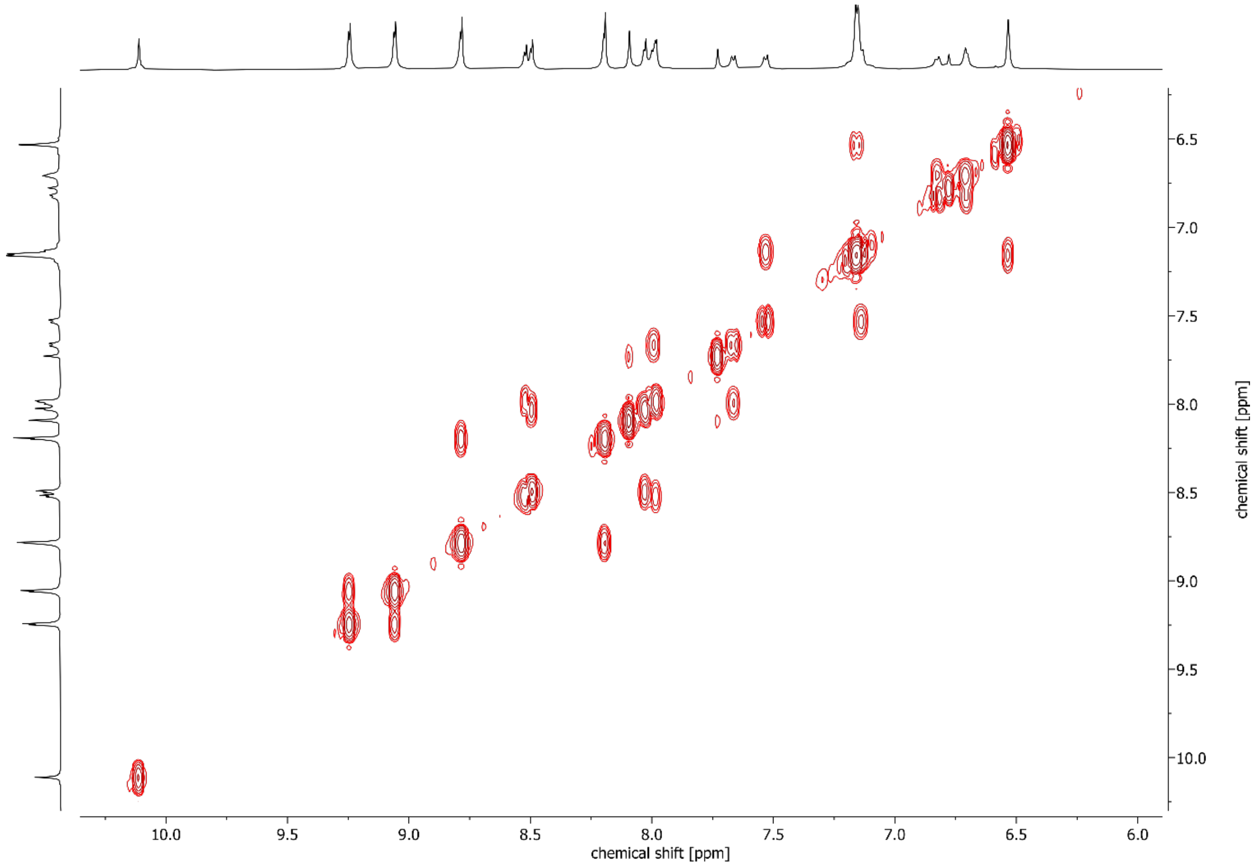

Figure S37. Selected aromatic region of a ^{1}H-^{1}H COSY NMR spectrum of **2**, CD_2Cl_2, 600 MHz, 298 K.

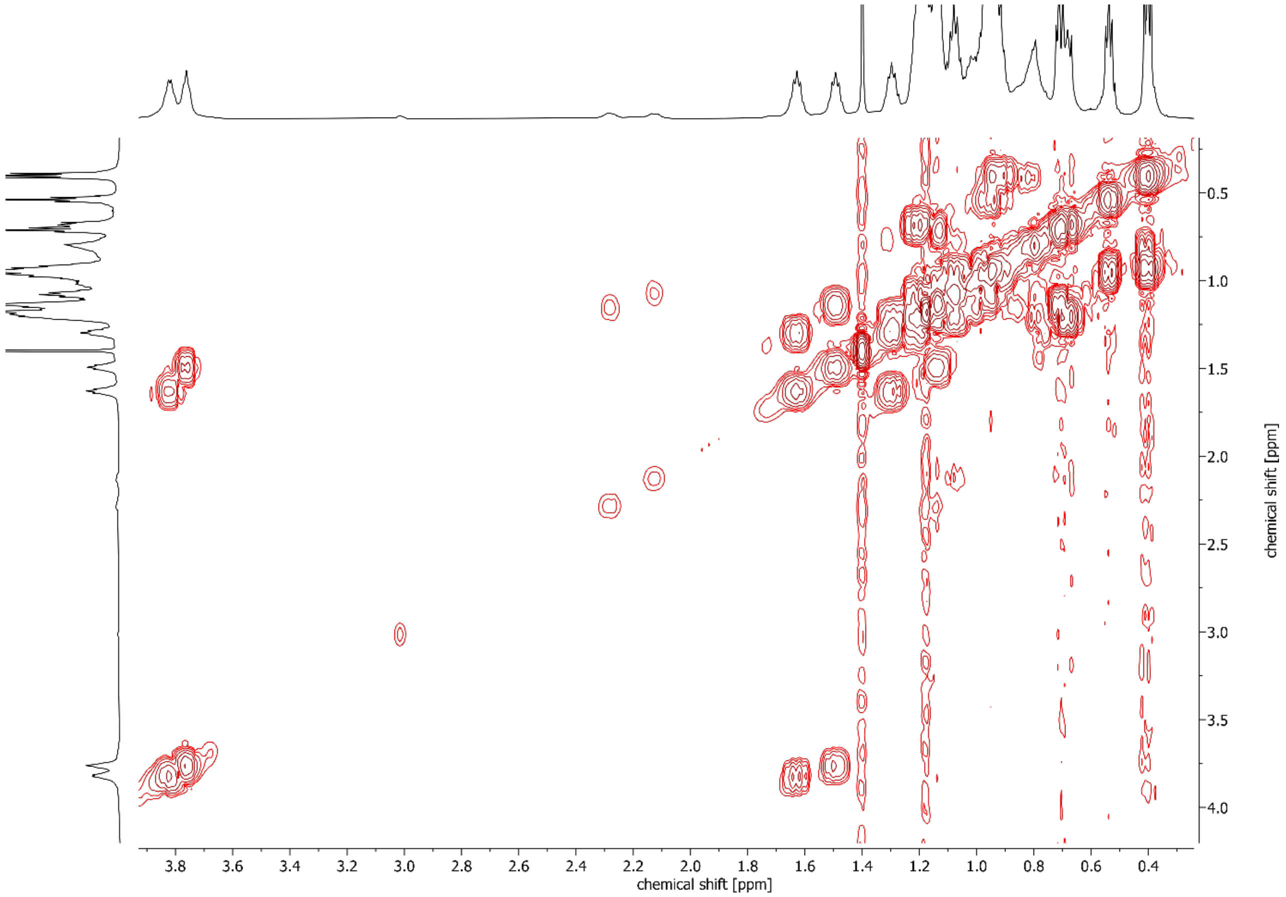

Figure S38. Selected alkyl region of a ^{1}H-^{1}H COSY NMR spectrum of **2**, CD_2Cl_2, 600 MHz, 298 K.

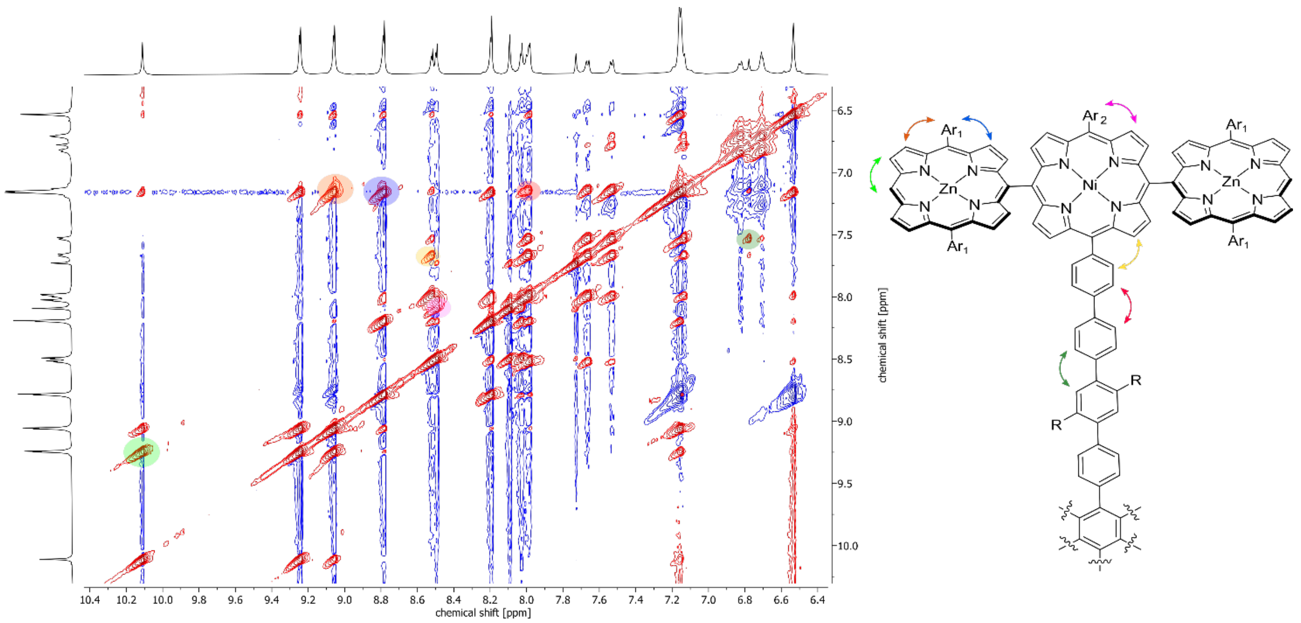

Figure S39. Selected aromatic region of a ^{1}H-^{1}H NOESY NMR spectrum of **2**, CD_2Cl_2, 600 MHz, 298 K with assigned correlations crucial for signal assignment.

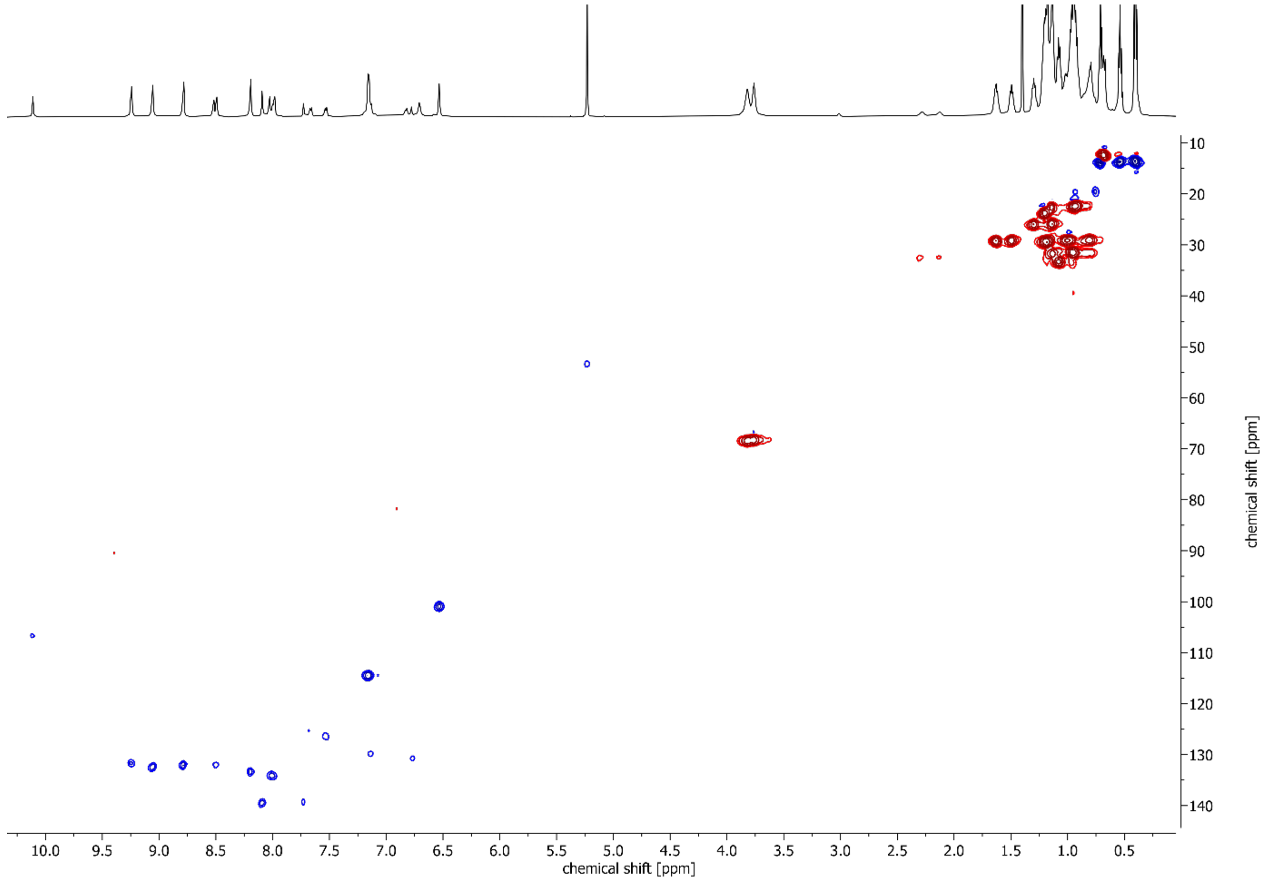

Figure S40. ^{1}H-^{13}C HSQC NMR spectrum of **2**, CD_2Cl_2, 600 MHz, 298 K.

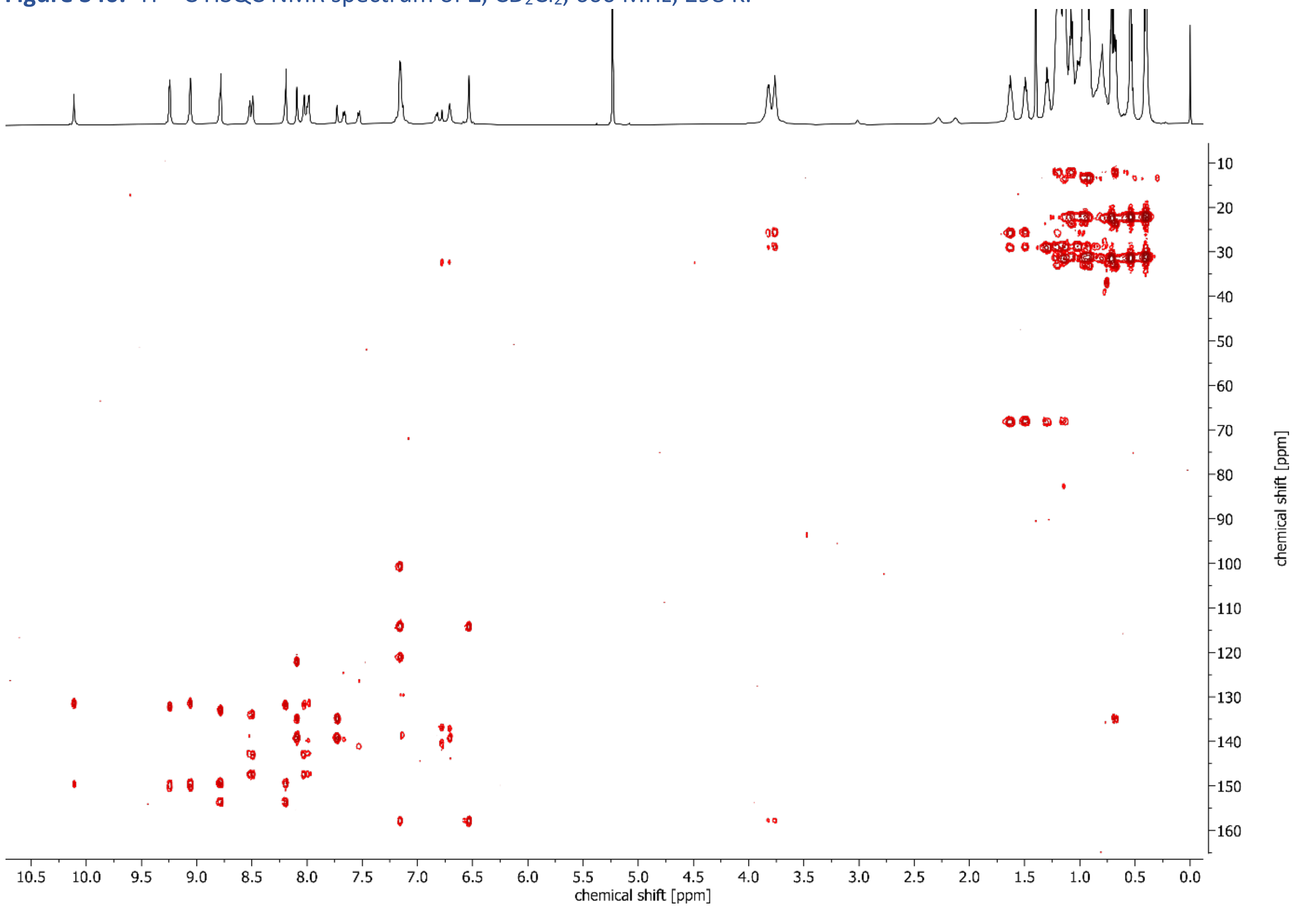

Figure S41. ^{1}H-^{13}C HMBC NMR spectrum of **2**, CD_2Cl_2, 600 MHz, 298 K.

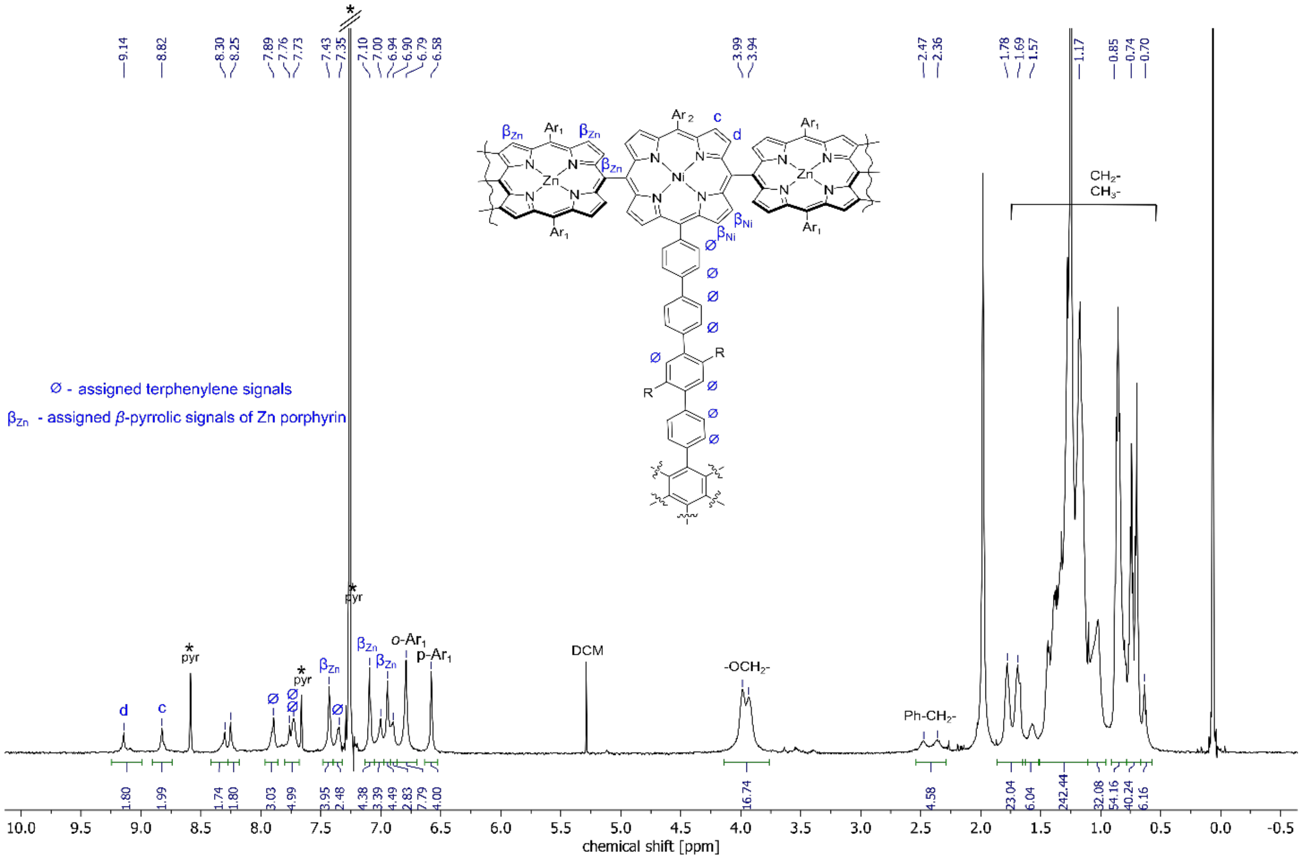

Figure S42. ^1H NMR spectrum of **1** ($CDCl_3$ + 1% pyridine-d_5, 600 MHz, 298 K) with partial signals assignment.

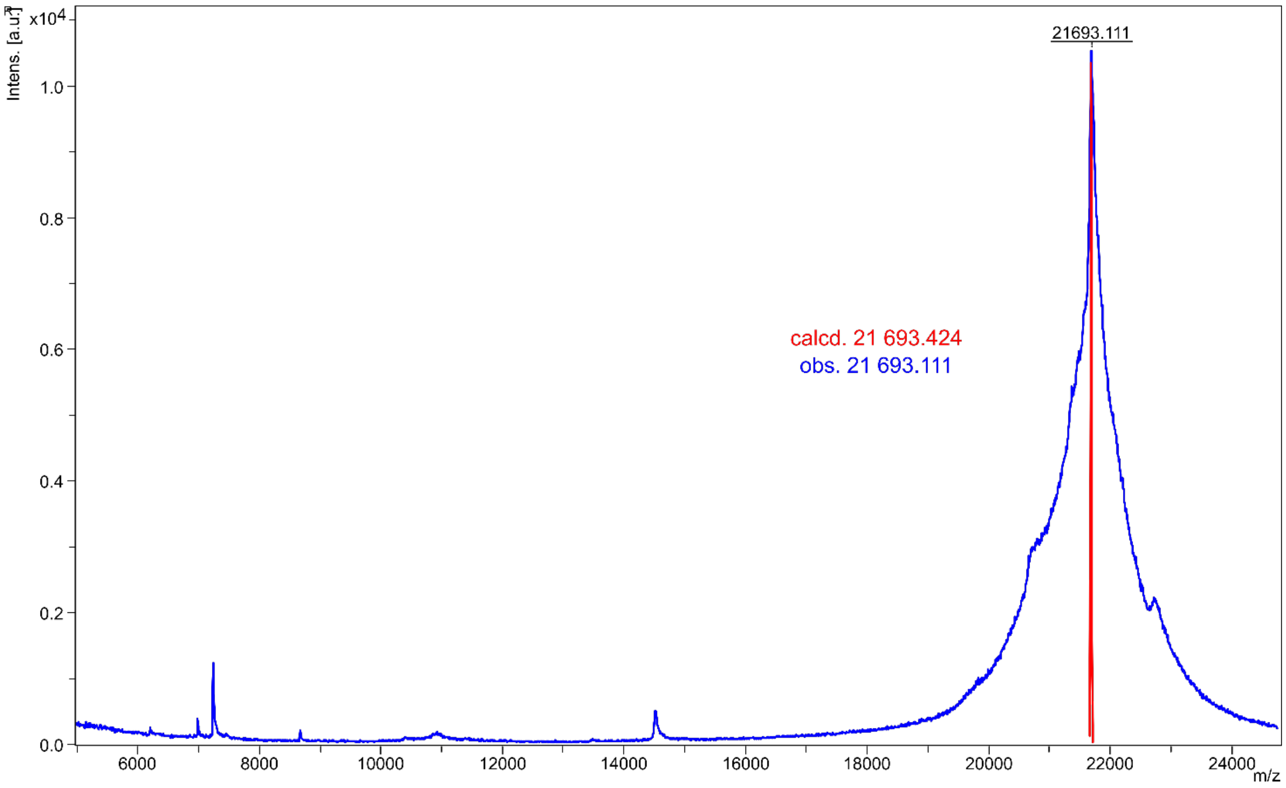

Figure S43. Calculated (red) and recorded (blue) MALDI mass spectra of **1**, DCTB matrix.

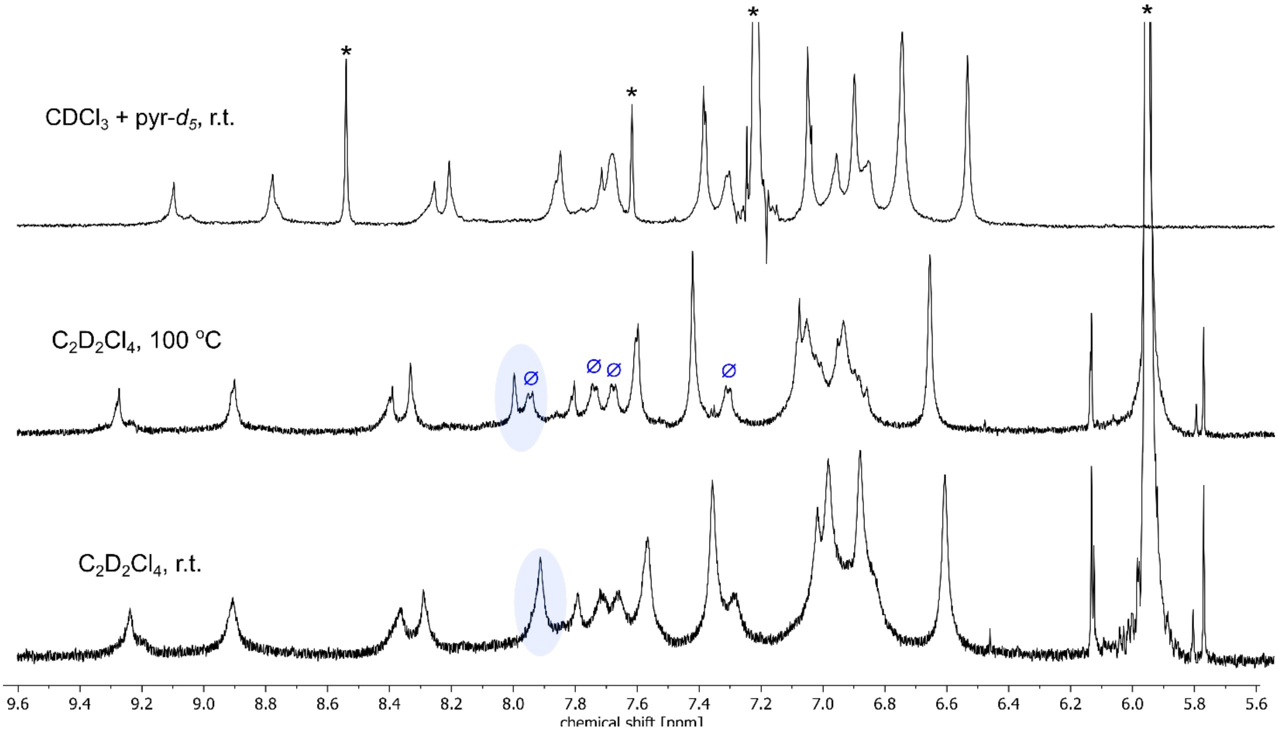

Figure S44. Comparison between NMR spectra of **1** recorded in chloroform at 25 °C with addition of pyridine-d_5 and in $C_2D_2Cl_4$ at 25 °C and 100 °C. Covalent core phenyl doublets become more resolved at higher temperature and it is possible to determine their coupling constants (ca. 8 Hz).

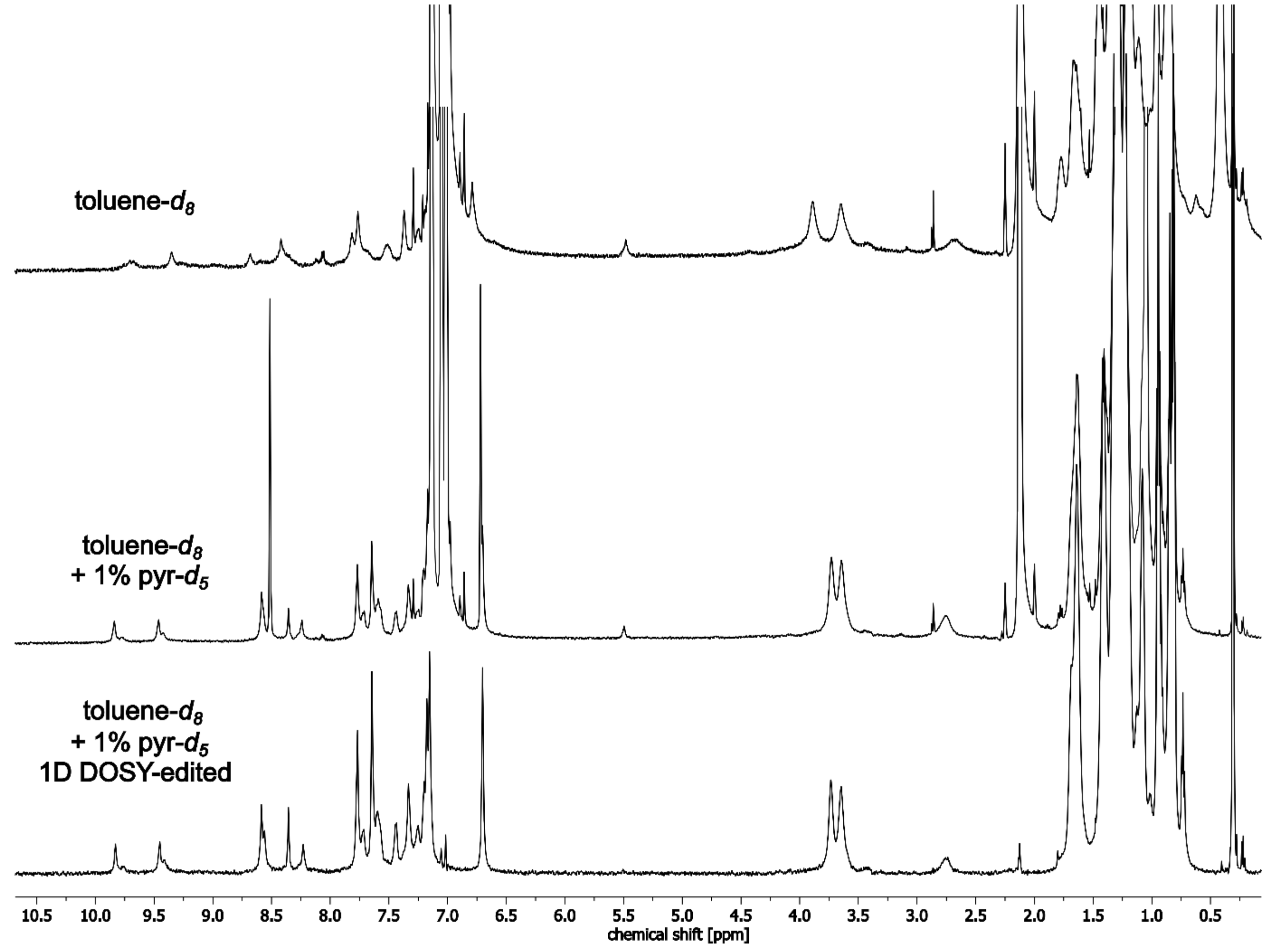

Figure S45. Comparison between ^{1}H NMR spectra of **1** recorded in toluene-d_8 before and after addition of pyridine and DOSY-edited spectrum (to remove the residual solvent peaks). Without pyridine, the sample aggregates strongly, whereas in the presence of pyridine, the peaks become sharper (600 MHz, 298 K).

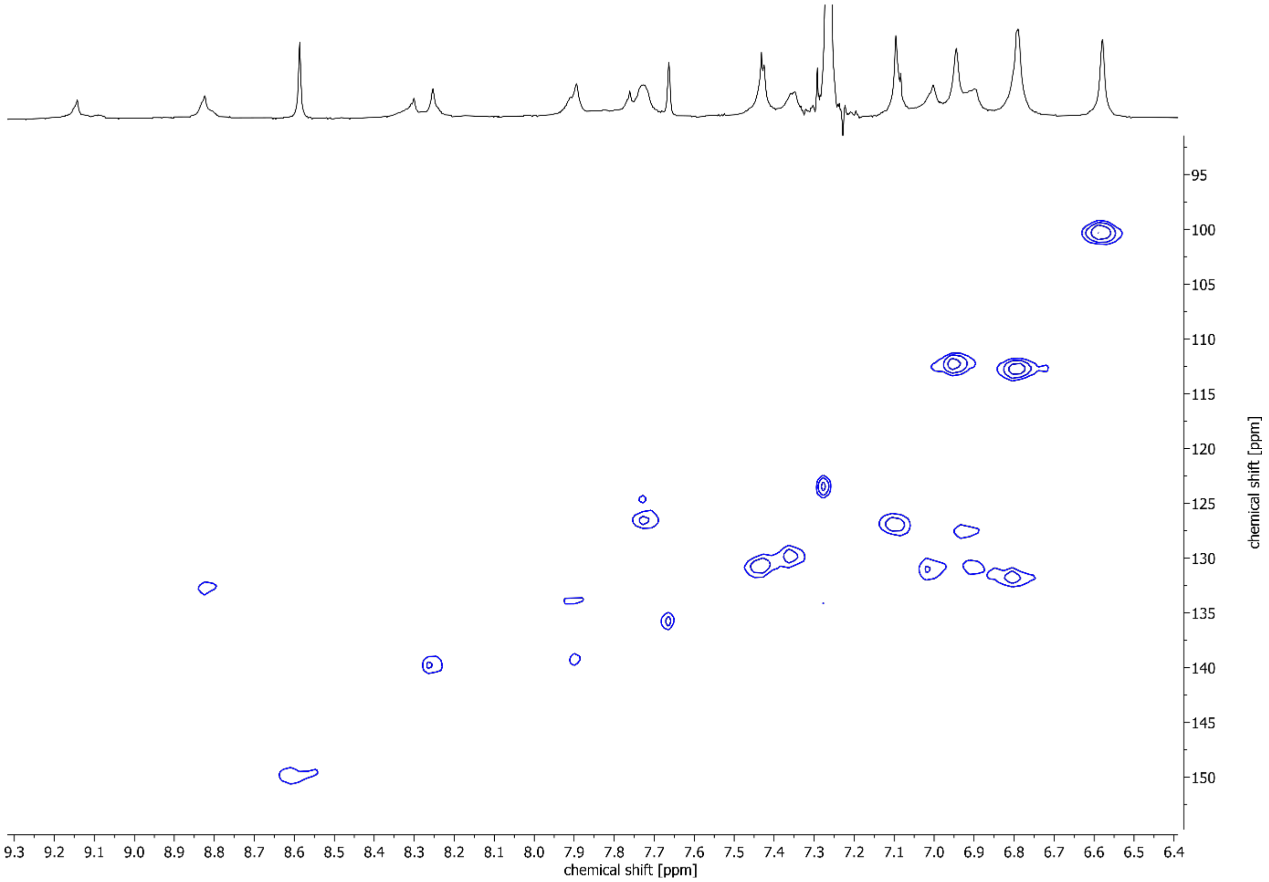

Figure S46. Selected aromatic region of a ^{1}H-^{13}C HSQC NMR spectrum of **1**, $CDCl_3$ + 1% pyr-d_5, 600 MHz, 298 K.

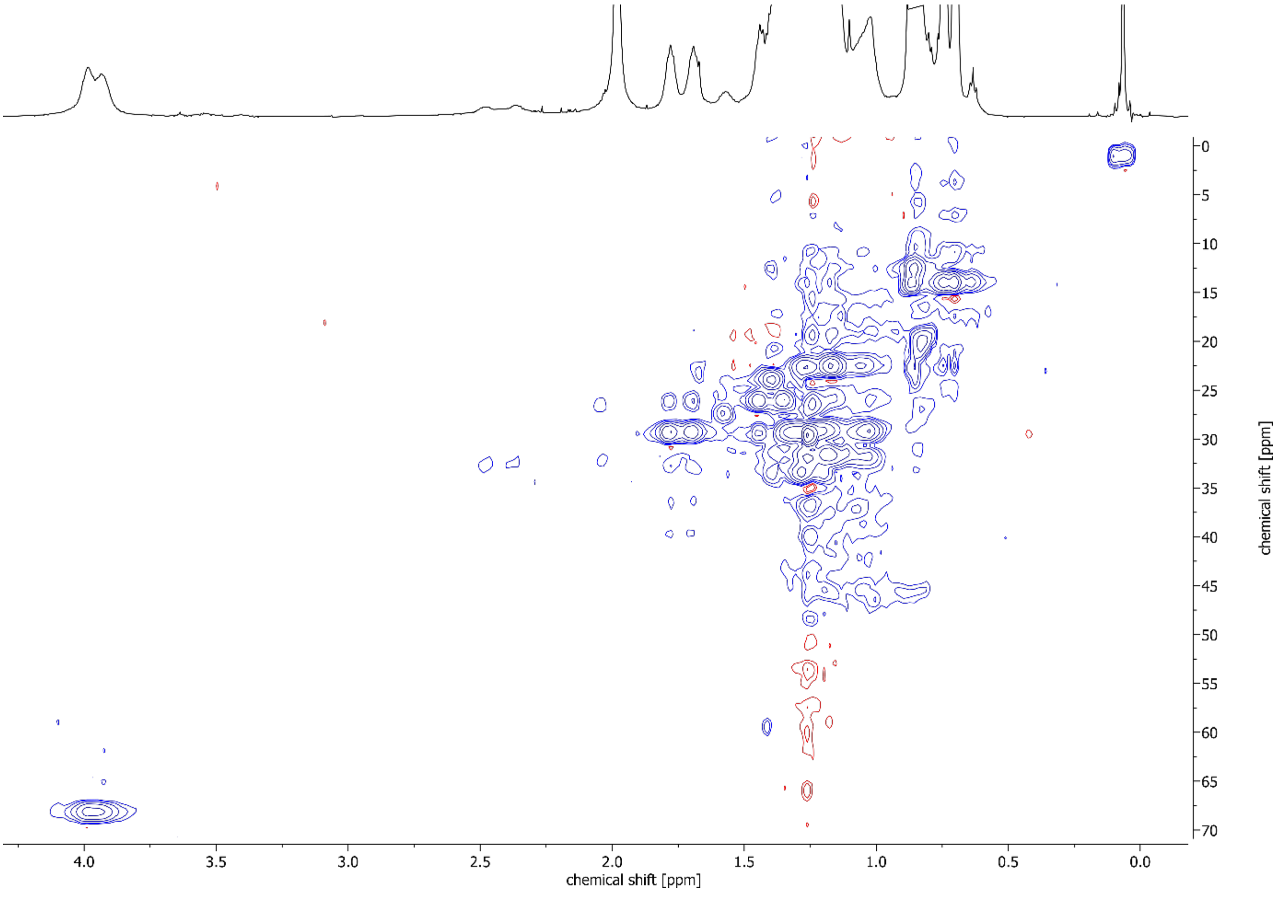

Figure S47. Selected alkyl region of a ^{1}H-^{13}C HSQC NMR spectrum of **1**, $CDCl_3$ + 1% pyr-d_5, 600 MHz, 298 K.

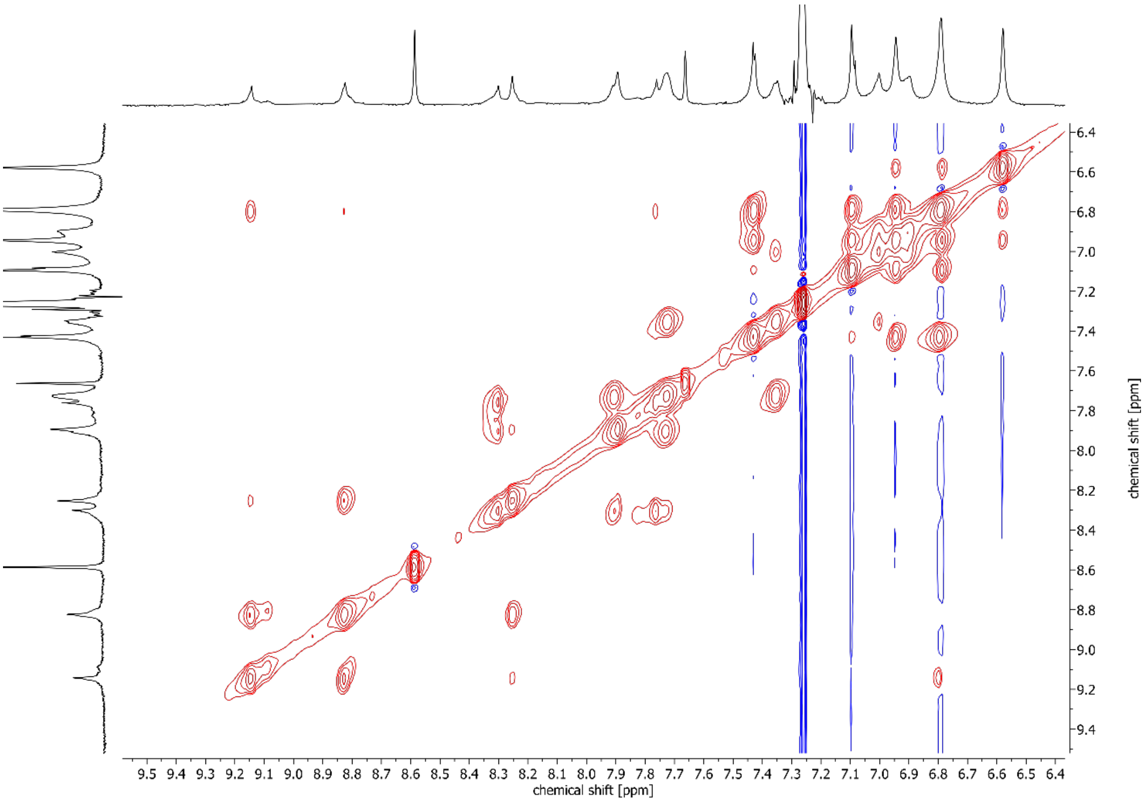

Figure S48. Selected aromatic region of a ^{1}H-^{1}H NOESY NMR spectrum of **1**, $CDCl_3$ + 1% pyr-d_5, 600 MHz, 298 K. Correlations crucial for signal assignment are marked in colors.

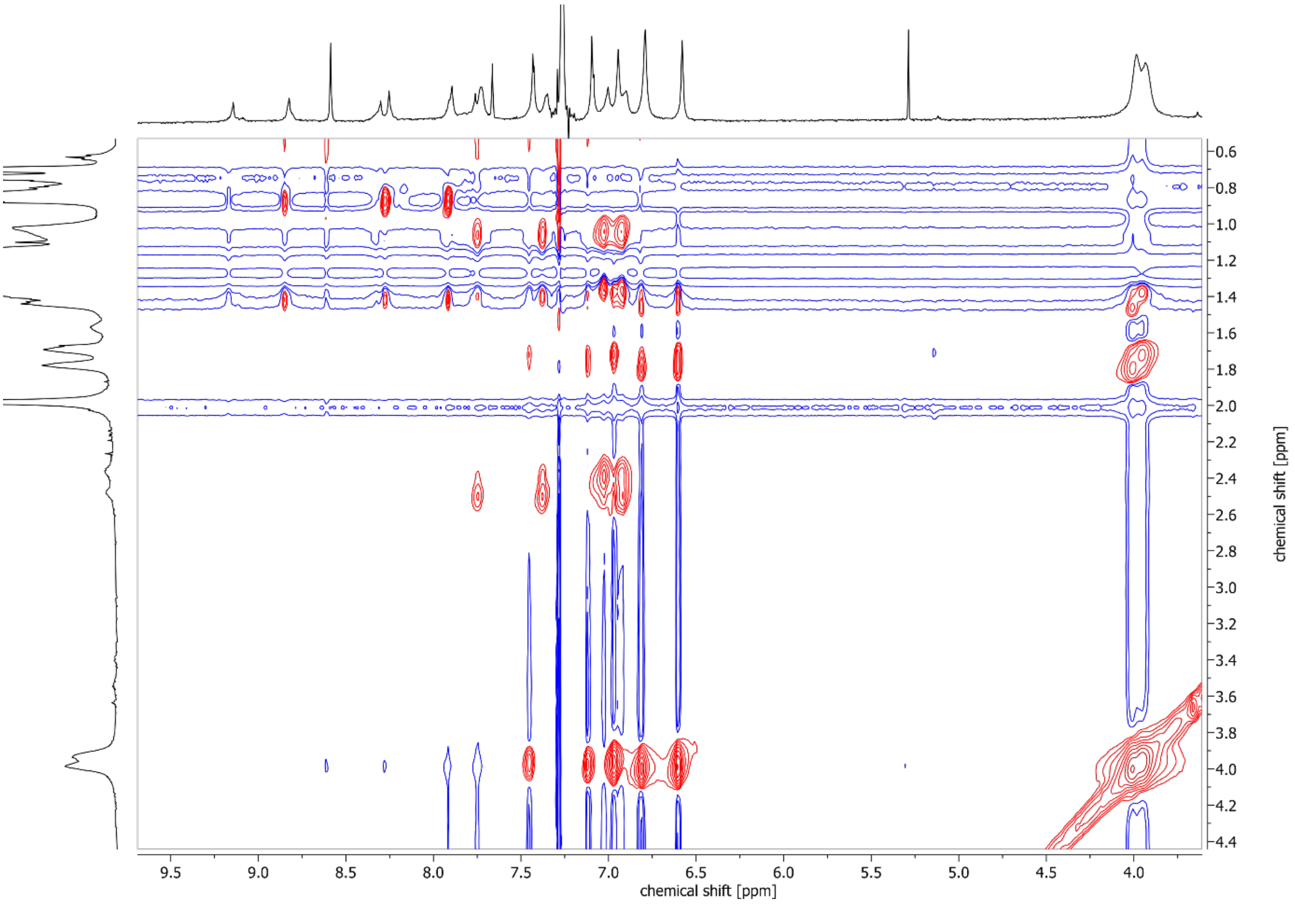

Figure S49. Selected region of a ^{1}H-^{1}H NOESY NMR spectrum of **1**, $CDCl_3$ + 1% pyr-d_5, 600 MHz, 298 K.

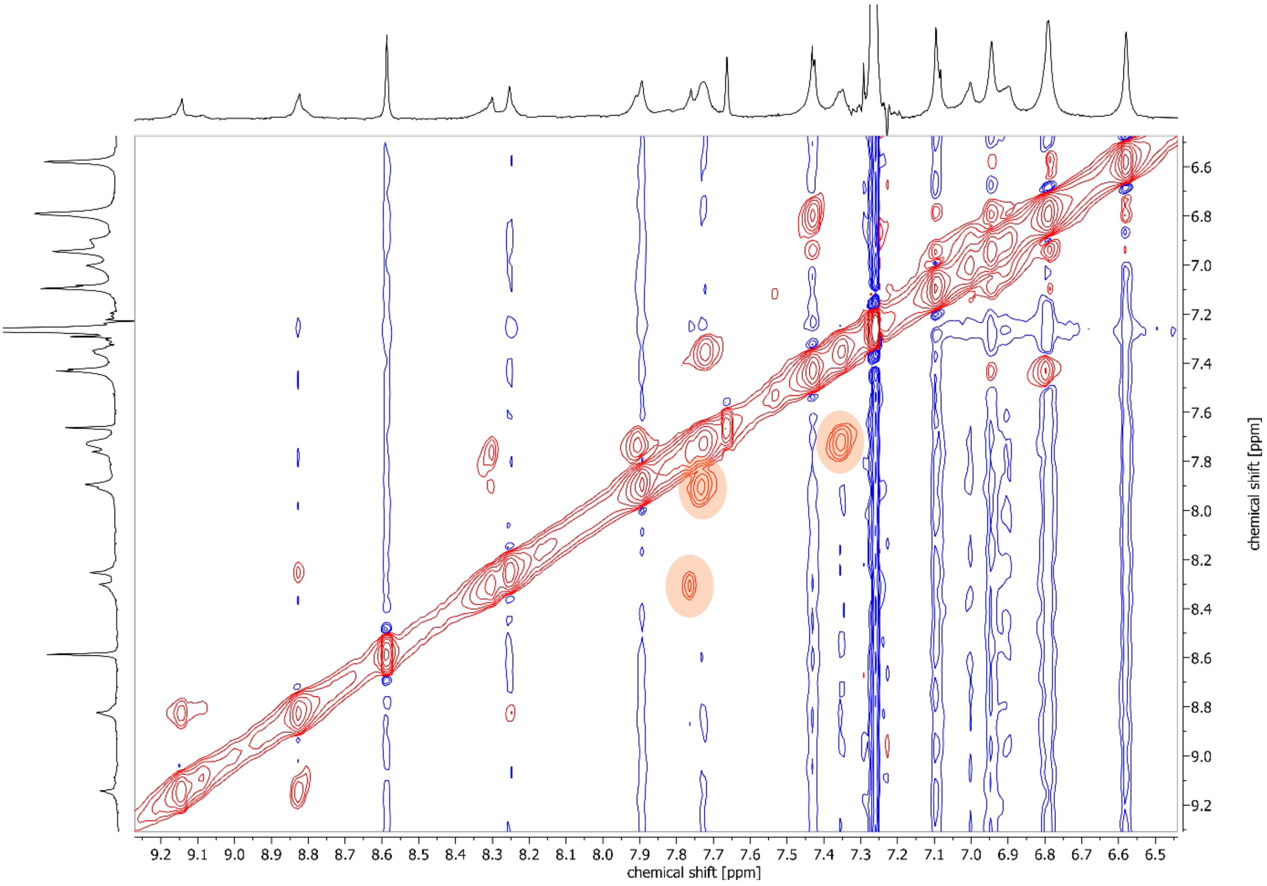

Figure S50. Selected aromatic region of a ^{1}H-^{1}H COSY NMR spectrum of **1**, $CDCl_3$ + 1% pyr-d_5, 600 MHz, 298 K. Correlations between the covalent core phenyls are marked in orange.

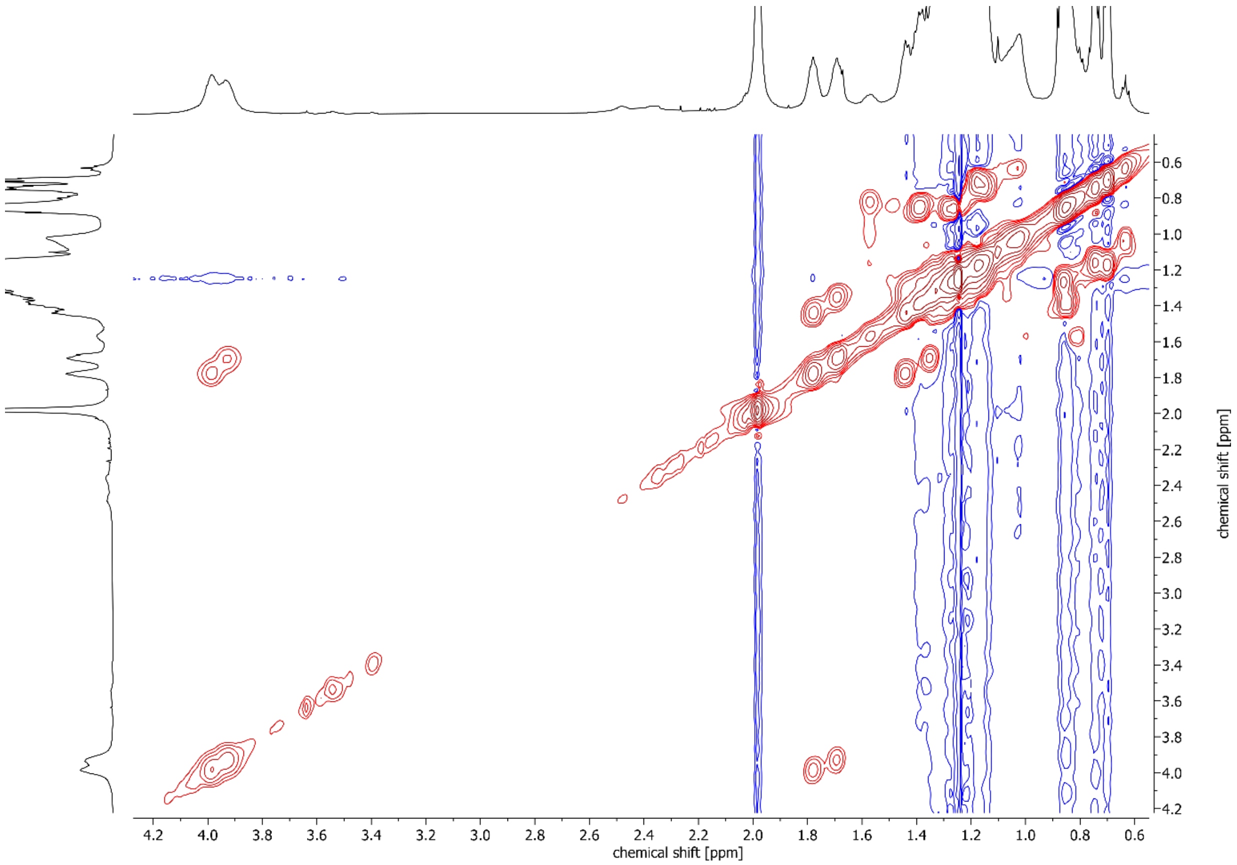

Figure S51. Selected aliphatic region of a ^{1}H-^{1}H COSY NMR spectrum of **1**, $CDCl_3$ + 1% pyr-d_5, 600 MHz, 298 K.

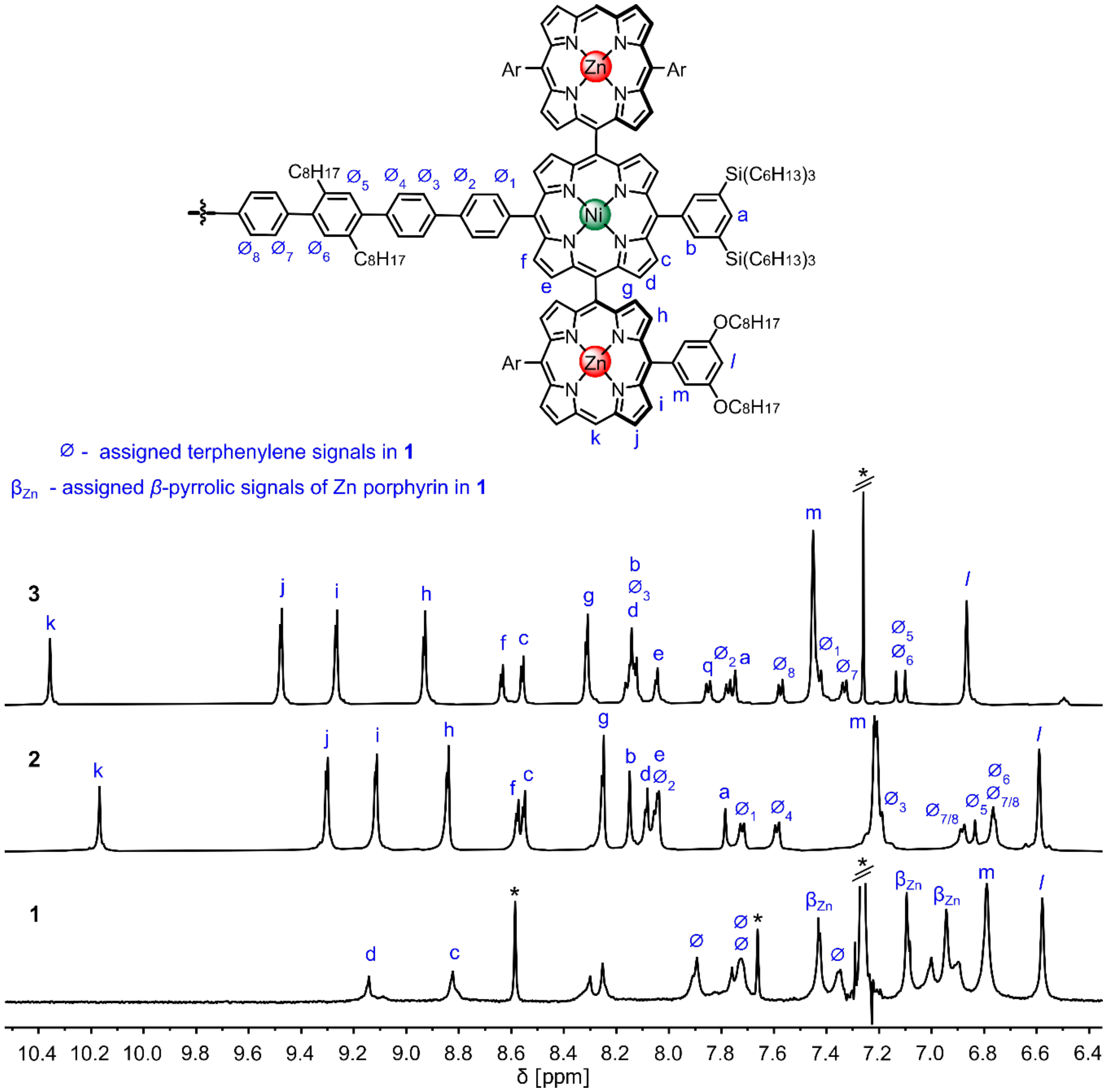

Figure S52. Comparison between NMR spectra of **3**,**2**,**1** and signals assignment. The signals for **1** can only be partially assigned due to substantial overlap and broadening. The positions of β_{Ni} protons after *meso*-*meso* coupling and edge-fusion of Zn porphyrins are consistent with a literature example of a linear tetramer[4] in which the β_{Ni} protons resonate in a region > 8.5 ppm (molecule shown on Fig. S51). On the other hand, during edge-fusion the β_{Zn} protons tend to move to lower chemical shifts, also consistently with the observed loss of one type of β_{Zn} protons and high-field shift of the three remaining ones.[4] Integration of aromatic peaks matches the expected values.

5. STM Studies

5.1. Experimental Methods

All scanning tunnelling microscopy (STM) images were acquired with an Omicron STM-1 system operating under ultra-high vacuum (UHV) conditions with a base pressure of 2×10^{-9} mbar. Images were acquired at room temperature in constant current mode using electrochemically etched tungsten tips, coated in gold during tip optimisation. Image acquisition parameters (sample bias and current set-point) are stated within figure captions. Au(111) on mica surfaces (Georg Albert PVD GmbH) were prepared by cycles of argon ion sputtering (0.76 keV for 20 minutes at a pressure of 9×10^{-6} mbar) and annealing (~500 °C, 20 minutes). Samples were transported between the STM and x-ray photoelectron spectroscopy (XPS) UHV systems using a vacuum suitcase with a base pressure of < 1×10^{-10} mbar. Porphyrin 18-mers **1** and **2** were deposited upon a clean Au(111) substrate via electrospray ionization with a Molecularspray UHV4i deposition source. 50 µg/mL solutions of **1** and **2** in toluene/methanol (3:1 ratio) were prepared. **1** was deposited with a solution flow rate of between 0.1 and 0.03 mL/hour for 20 minutes using a potential of 2 kV to initiate the electrospray event (pressure during deposition was 2×10^{-7} mbar). **2** was deposited with a solution flow rate of between 0.1 and 0.03 mL/hour for 40 minutes using a potential of 2 kV to initiate the electrospray event (pressure during deposition was 2×10^{-7} mbar). During the deposition of **2**, the electrospray spot was raster-scanned across the sample.

5.2. Details of Nanorings Characterization via STM

Nanoring dimensions were measured from line profiles acquired along the long- and short-axis of the rings; as shown in Fig S53. Peak-to-peak measurements were obtained, to determine the separation between the features corresponding to the position of the nanoring circumference. Measurements were performed on data acquired with both 'forward' and 'backward' scan directions. An average of the two measurements was taken, to minimize the effect of drift. The circumference of the rings was estimated using the Ramanujan approximation for the circumference of an ellipse:

$$C \approx \pi[3(a+b) - \sqrt{(3a+b)(a+3b)}],$$

where *C is* the circumference of the ellipse, with *a* and *b* being the radii of the long- and short-axis of the ellipse, respectively.

Using the method previously described, the dimensions of **1** and **2** were obtained following deposition onto Au(111); as demonstrated in Figure S54 (Fig. S54a shows **1**, and Fig. S54b **2**). The average dimensions of the two were measured as follows: **2** circumference = 15 ± 1 nm, long axis = 5.5 ± 0.3 nm, short axis = 4.3 ± 0.2 nm. **1** circumference = 12 ± 1 nm, long axis = 4.1 ± 0.3 nm, short axis = 3.7 ± 0.3 nm. Due to difficulties in obtaining stable imaging conditions, the number of **2** rings suitable for measuring was limited, with a total sample size of 8. The sample for **1** consisted of 60 individual rings.

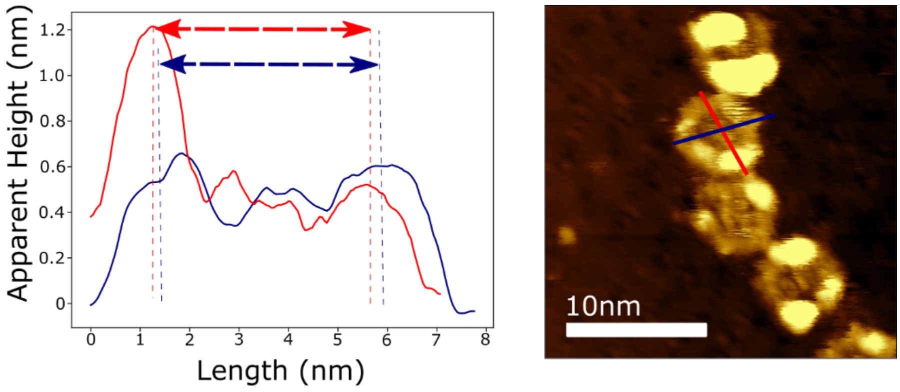

Figure S53. Line profiles acquired for the long- and short-axis of **1** deposited on an Au(111) surface (line-profile locations are indicated within the STM image). The peak-to-peak separation on the red and blue line profiles are 4.3 nm and

4.4 nm, respectively. The STM topograph shows **1** on Au(111) following electrospray deposition (sample bias = –2 V, set-point current = 15 pA).

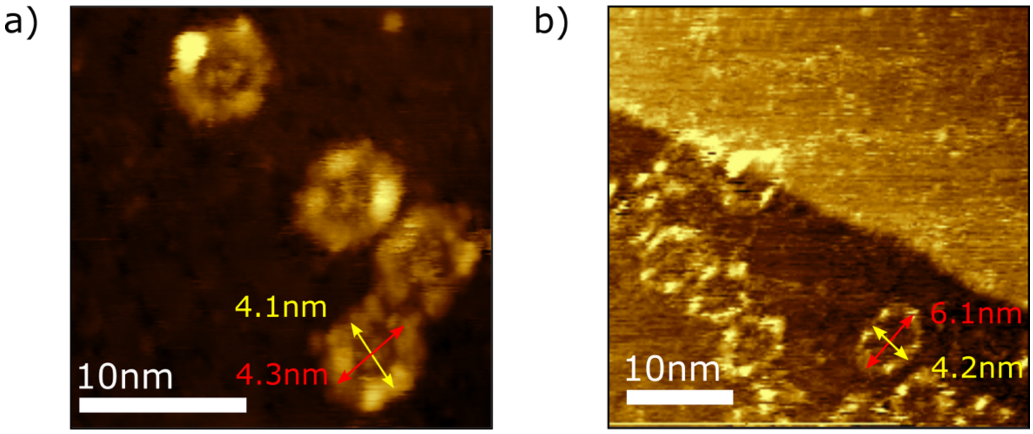

Figure S54. STM topographs of (a) **1** and (b) **2** following electrospray deposition onto Au(111) substrates. Measurements of the ring dimensions were acquired from this type of image (measurement positions indicated by red and yellow arrows). Image parameters: (a) sample bias = –2 V, set-point current = 15 pA. (b) sample bias = –1 V, set-point current = 20 pA.

5.3. Characterization of nanoring internal scaffold

The internal scaffold of **1** is observed in STM imaging. Figure S55 shows an STM topograph of several **1** adsorbed at the Au(111) surface; the associated line-profile reveals the presence of a topographic feature at the center of the ring which is assigned to the presence of the covalent template. Such features were not observed previously during measurements of a template-free 24-porphyrin nanoring.[5]

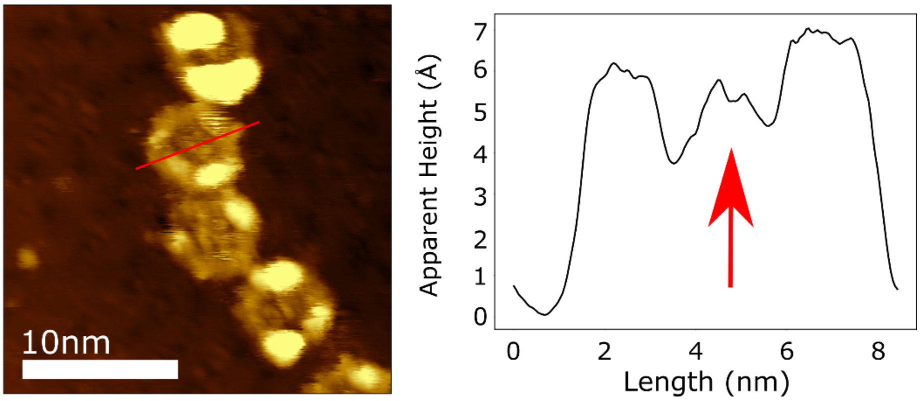

Figure S55. STM image of **1** deposited onto Au(111), with a line profile taken across the center of the ring (sample bias = –2 V, set-point current = 15 pA). The location of the line profile is indicated by the red line. The red arrow points to the raised feature in the center of the ring; assigned to the presence of the template.

5.4. Characterization of 1 and 2

The dimensions of the long- and short-axis of **1** and **2** were acquired for both species following electrospray deposition onto two separate Au(111) substrates. Figure S56 displays the measured dimensions as well as the average circumference (as estimated by the Ramanujan approximation) for each species and black solid/dashed lines representing flattening factor, *f*:

$$f = \frac{a-b}{a},$$

where *a* and *b* are the long and short, respectively, radii of the ellipse defined for each nanoring species. The majority of **1** species have a flattening factor of $f < 0.2$ (indicating a limited deviation from circularity) with **2** species showing increased flattening ($f < 0.4$). This is attributed to the increased rigidity of **1** due to the fusing of the Zn porphyrin species.

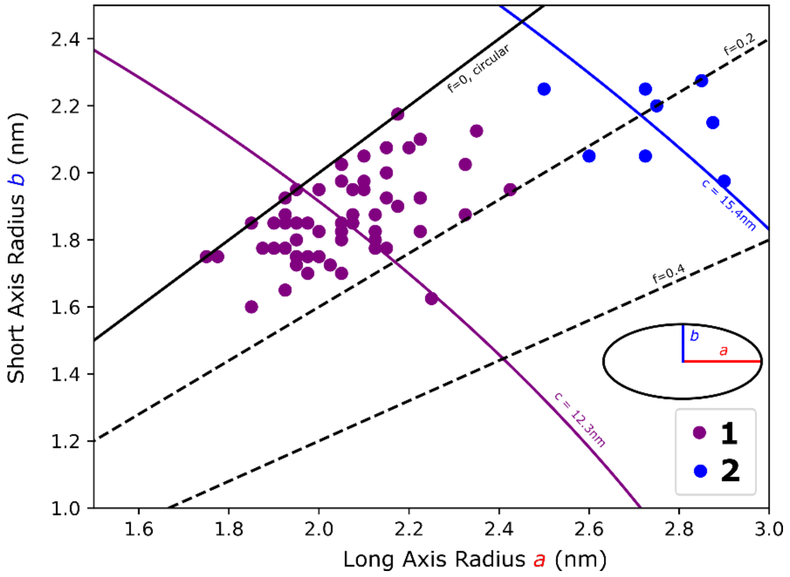

Figure S56. Graph showing experimentally measured long- and short-axis for **1** and **2** nanorings deposited onto Au(111). The blue and purple arcs correspond to an ellipse of fixed circumference; equivalent to the average circumference obtained for each nanoring deposition (circumference = 12.3 nm for **1**, 15.4 nm for **2**). The solid black line represents a flattening ratio of 0, indicating a circular ring shape. The dotted lines represent flattening ratios of 0.2 and 0.4, the shape becoming more elliptical with increasing *f* value. The dataset for **1** and **2** is based upon 60 and 8 rings, respectively.

5.5. Overview of surface following electrospray deposition

Sub-monolayer coverages of **1** and **2** were obtained following electrospray deposition. Large-area STM topographic images showing the presence of individual molecules and small clusters of **1** and **2** are shown in Figure S57.

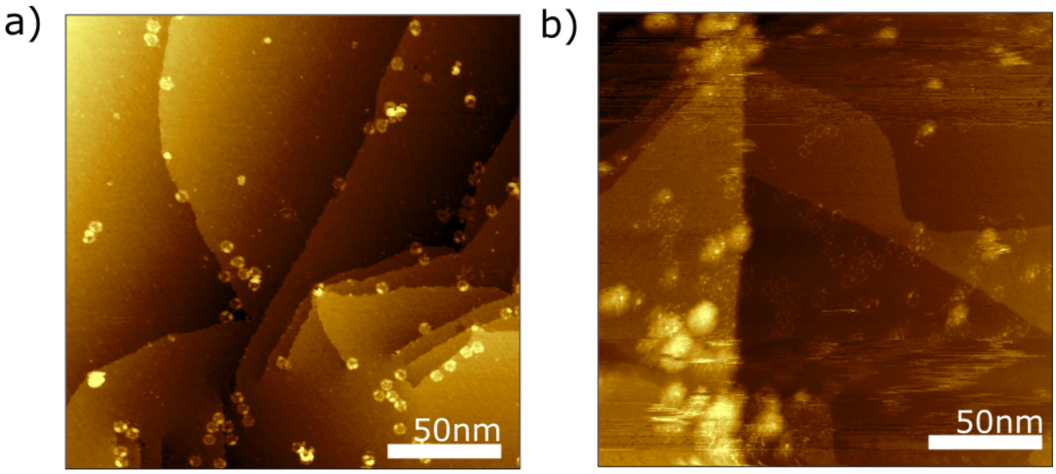

Figure S57. STM topographic images showing the distribution of (a) **1** (sample bias = –2.0 V, set-point current = 15 pA) and (b) **2** (sample bias = –1.0 V, set-point current = 20 pA) on Au(111) following electrospray deposition.

5.6. X-ray photoelectron spectroscopy (XPS) characterization

X-ray photoelectron spectroscopy (XPS) was acquired using a SPECS DeviSim near ambient pressure XPS (NAP-XPS) instrument operating in ultra-high vacuum (UHV) mode at a pressure < $1x10^{-9}$ mbar. Spectra were measured using a Phoibos 150 NAP hemispherical analyzer with 20 eV pass energy and monochromatic Al $K\alpha$ X-rays (1486.7 eV). All spectra are calibrated with respect to the Au 4f7/2 peak at 83.95 eV.

Analysis of the Ni 2*p* and Zn $2p_{3/2}$ regions reveals features consistent with the presence of Ni- and Zn-porphyrin species. For **1** the Ni $2p_{3/2}$ peak is observed at a binding energy (BE) of 855.3 eV, consistent with a Ni(II) oxidation state[6] and clearly distinct from the expected BE of metallic Ni (852.6 eV);[7] for **2** the Ni $2p_{3/2}$ peak is observed at a similar position 855.1 eV. The Zn $2p_{3/2}$ region reveals peaks at 1022.1 eV and 1021.4 eV,

for **1** and **2** respectively; definitive identification of the Zn(II) state, compared to Zn(0), is non-trivial.[8] The shift to higher BE, of **1** relative to **2**, is assigned to the conformational differences between the two species. The 'non-fused' structure of **2** provides greater flexibility and facilitates an increased interaction with, and proximity to, the Au surface, as compared to **1**; we therefore assign this shift to higher BE to a reduced screening effect form the Au surface.

The ratio of Zn to Ni species was obtained by comparing the ratio of the fitted peak areas of Ni 2$p_{3/2}$ and Zn 2$p_{3/2}$ [normalized to the relevant photoionization cross-sections]. The calculated Zn:Ni ratio is 2:1, in agreement with the expected ratio of Zn- to Ni-porphyrins in both **1** and **2**.

For both **1** and **2** the N 1*s* region shows a feature which can be fitted to a single peak (BE 398.6 eV and 398.3 eV for **1** and **2**, respectively). A single peak N 1*s* is indicative of metalated porphyrin species,[9] and the calculated N:Zn ratio of 6:1 (obtained from the fitted peak areas of N 1*s* and Zn 2$p_{3/2}$ - normalized to the photoionization cross-sections), is in excellent agreement of the expected ration of N to Zn in **1** and **2**.

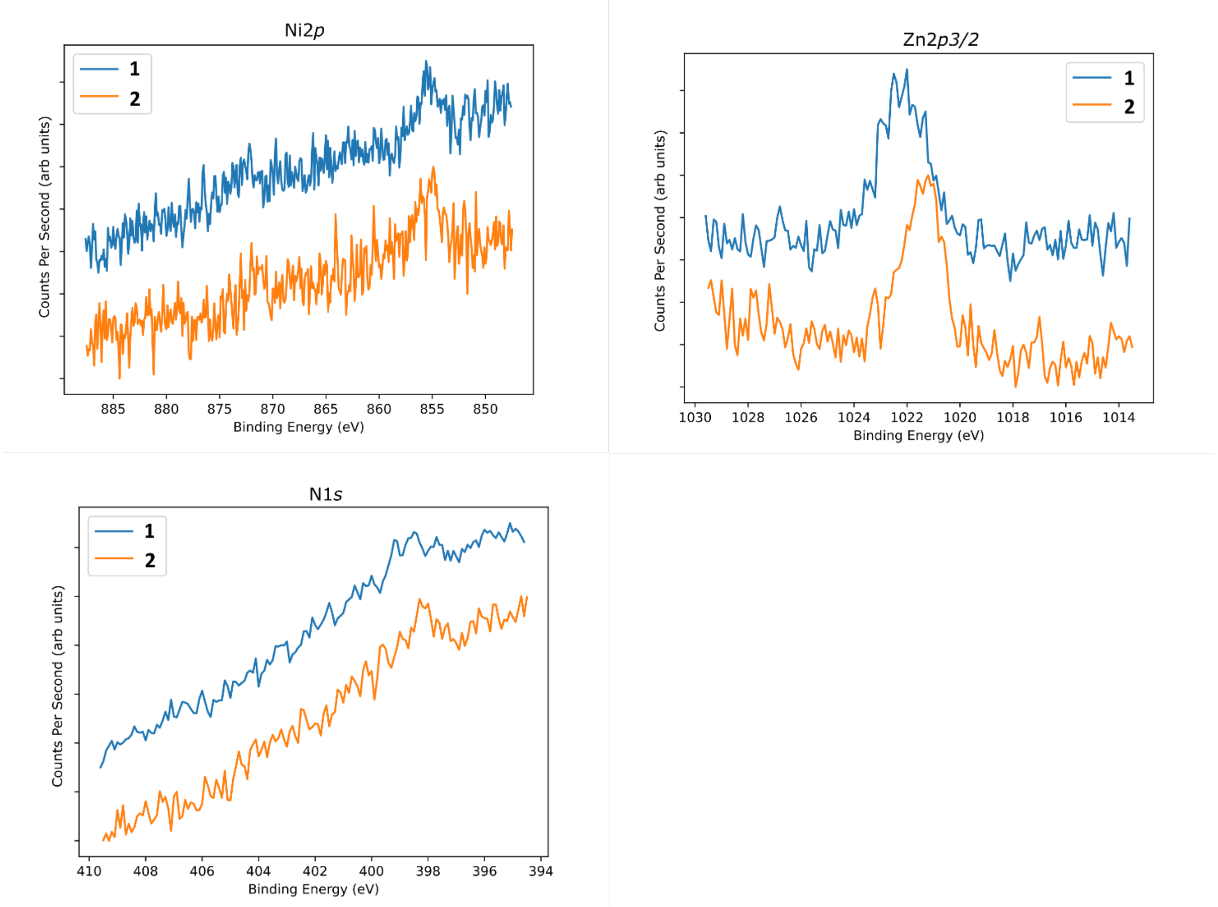

Figure S58. XPS analysis of the Ni 2*p*, N 1*s* and Zn 2$p_{3/2}$ regions. The N 1*s* spectra indicate a single nitrogen environment, and the presence of Ni and Zn suggests deposition of metallated porphyrins. Note: **2** spectra are for 'as-deposited' samples. **1** spectra were acquired following anneal (to remove excess solvent)

6. Calculated Molecular Geometries

Geometries were optimized using semiempirical tight binding *xTB* software (version 6.4.1) using *GFN1* parameters.[10] We calculated all the vibrational frequencies and none of them were imaginary. Alkyl and aryl solubilizing groups were replaced by hydrogen atoms to simplify the calculations.

5.1. Optimized geometries

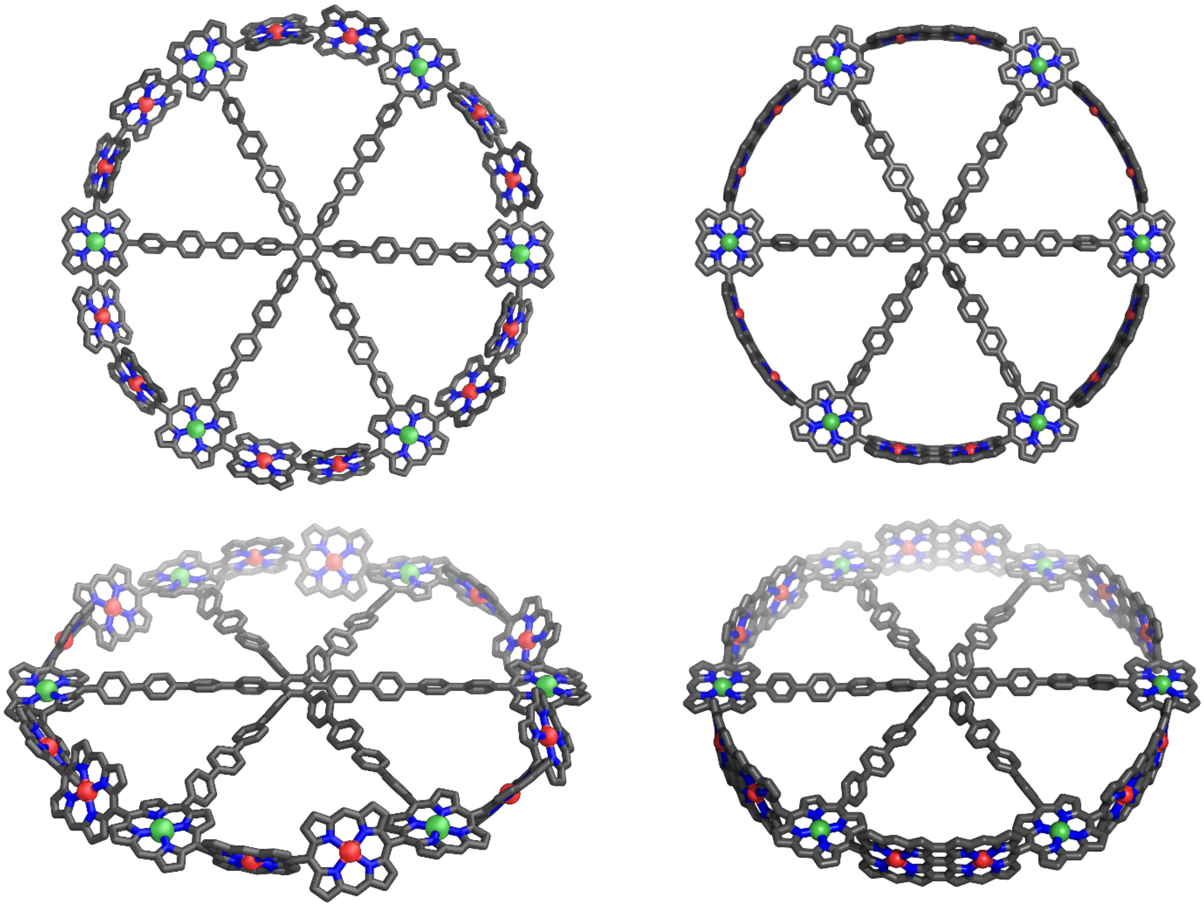

intermediate meso-meso linked ring

1

Figure S59. Optimized geometries of an intermediate *meso-meso* linked nanoring (not isolated, left) and **1** (xTB gfn1, right).

6.2. Cartesian coordinates

<u>*Meso-meso* linked intermediate nanoring</u>

total E = -1488.109640273214 Ha

C	-21.06182012125783	-3.32011068025697	0.57996485725008
C	-20.84960738355983	-3.88938678646352	-0.72514975478910
C	-20.25615352337072	-3.22345525259072	-1.80732798798097
C	-19.93204023414346	-3.80553305766126	-3.03751646148943
C	-19.27183012012685	-3.14830775460759	-4.13479693479310
C	-19.13800834415476	-4.06385049701707	-5.12684185822233
C	-19.74811778229369	-5.28062468994155	-4.66426118903151
N	-20.20838786857076	-5.09416364100144	-3.39253539658350
C	-21.66524866724693	-4.26984687635990	1.33746213885290
C	-21.84734773925024	-5.42282027811207	0.49956879980284
N	-21.31815337289633	-5.16849636063169	-0.73396832679961
C	-22.53849684111650	-6.57345024232706	0.85768646254165
C	-22.84600253341167	-7.65235751689262	0.03643869592351
N	-22.42763993844694	-7.81838684886352	-1.25269094507948
C	-23.00877111295170	-8.95559232153662	-1.73306789334684
C	-23.83922857013660	-9.52547675802147	-0.70356538037952
C	-23.71881488967487	-8.73590201999255	0.39252183419362
N	-21.30621143844158	-7.75662508641962	-3.90562232772708
C	-20.62806720527067	-7.57322817889020	-5.07669274259963
C	-19.90176393463867	-6.43790617822442	-5.41994149507493
C	-20.86124733305557	-8.70125113426414	-5.93641916661944
C	-21.70906413423699	-9.53331292565153	-5.28056957709876
C	-21.99119875072338	-8.93184344343664	-4.00399248860442
C	-22.83258882633272	-9.47356416288971	-3.02418057483861
H	-20.77102081410258	-2.32404992085486	0.87358603027479
H	-18.94861725643294	-2.11963058400247	-4.13491881517828
H	-18.68812399911396	-3.93163715523332	-6.09849840201446
H	-21.97599723072025	-4.20248187748263	2.36834226243266
H	-22.93444549319886	-6.60621279228414	1.86806133275707
H	-24.19344077147774	-8.85437132622622	1.35406647601473
H	-19.45472005820147	-6.43170085912775	-6.40937713102586
H	-20.43436732974602	-8.82448462993215	-6.91953966991505
N	-39.19488854948825	-21.01032439351591	-3.63949465428548
C	-37.26665082189309	-19.87946252956641	-2.63074473230500

C	-37.81467159497414	-19.63738235748971	-1.36246687456135
C	-37.10265209834388	-19.06460085795121	-0.25001099988068
C	-37.97580021495969	-18.98310553842314	0.78481611375838
C	-39.23591694034761	-19.48629446698597	0.31218586949321
N	-39.09632784338713	-19.89938640717336	-0.98256645184983
C	-37.91060269018799	-20.54954685367335	-3.67655431715630
C	-37.34088830384877	-20.85278047104498	-4.96318824029809
C	-38.28688008454072	-21.51783384581616	-5.67245976096502
C	-39.45926302984938	-21.59581656374740	-4.84416564703561
C	-40.68407018169400	-22.12473305877861	-5.23664841182794
C	-40.42724587344414	-19.46357305280803	1.02636732963594
C	-41.68715221901225	-19.82205283593033	0.56030142017539
N	-41.96056547717448	-20.36511732656718	-0.66225707633381
C	-43.31499403135407	-20.49545966466902	-0.76351707023438
C	-43.92261834942264	-20.00146238501087	0.44522068808591
C	-42.91846755936368	-19.61014837871040	1.26858092628602
N	-42.06116877737809	-21.49541868170181	-3.30997475224787
C	-41.88442420206385	-22.06954226822865	-4.53639634279352
C	-43.39460802994639	-21.52929876528412	-3.02571763524101
C	-44.08740636952122	-22.18210801191221	-4.10500475069739
C	-43.15548489855085	-22.52389622251810	-5.03018765619418
C	-43.99914037179819	-21.01557267988423	-1.87148836130786
H	-36.06357037363820	-18.77651905699278	-0.26441593286595
H	-37.79739141215511	-18.60570110620290	1.77957738042786
H	-36.34153764666291	-20.59285594790450	-5.27356711316678
H	-38.21588891013005	-21.90562335891539	-6.67673395821705
H	-40.72067902806876	-22.57865163467448	-6.22228839139566
H	-40.37987424453201	-19.06516532285465	2.03519896519603
H	-42.99493823545524	-19.19152012016132	2.25993283773053
H	-43.30524733679619	-23.02865942997267	-5.97184463056130
C	-42.22839544966510	28.08594375767434	6.85882351601349
C	-48.38733596634184	24.91262888289614	9.30287441090661
C	-47.48973460693083	24.21195202410522	10.12152030423178
C	-47.85662868334179	23.18342135601560	11.05956759914956
C	-46.71274844691310	22.74986262034551	11.64567130010748
C	-45.63194303569885	23.49295196718460	11.05893977633685
N	-46.13945927860367	24.38807270693466	10.16029247737245
C	-44.28182304932962	23.26742246374593	11.29613891990017
C	-43.20849899112377	23.87420172433502	10.65356562875488
N	-43.29391003404535	24.88489145261550	9.73947083891770
C	-41.83440156451674	23.48105619244352	10.79528294045148
C	-41.11276965677966	24.24264988108907	9.93586584819013
C	-42.03267406038598	25.14003229995067	9.28571593893244
N	-46.79872078273652	26.49169092854017	8.30411203154334
C	-46.88458726719809	27.53622262898556	7.42917898983490
C	-48.25939634299480	27.72248723757185	7.05246408918399
C	-48.97935755653125	26.76041252988391	7.68202236671060
C	-48.05828095337543	25.99709231222075	8.48254026093304
C	-45.81042966129029	28.26274905278731	6.92689256376688
C	-44.45823118714940	28.00920169683347	7.13072952778113
N	-43.94739820358762	26.99938817121548	7.89506096701691
C	-42.59282603280699	27.00553060679296	7.73680706797160
C	-41.69487135706805	26.11035750355436	8.33157309188419
C	-43.37673099839906	28.71039291680005	6.49461352904794
H	-48.86510062091404	22.85120302780128	11.24807360657323
H	-46.59753643139354	21.98646925611593	12.39918088593447
H	-44.03922412091721	22.49019724395304	12.01431511789863
H	-41.48086482746605	22.70286426979360	11.45354411098640
H	-48.61394446996358	28.48191853981318	6.37309080052471
H	-50.04085270690055	26.58113265306725	7.62106068220101
H	-46.05233106662622	29.08078112438318	6.25520133781939
H	-43.49410136796202	29.56333586864368	5.84449954445
C	-20.97129968558308	16.79663334900349	-0.01422254372837
C	-20.78008481666785	15.50662789114302	0.36069944365022
C	-21.67709007675148	14.06921126663082	2.22774734536330
N	-23.08500140860124	14.83479038486604	4.09126457960567
C	-25.80342569806504	19.16013578141022	4.28472829177073
C	-25.67372468182706	18.05914111112414	5.14357510790370
C	-26.42388803665045	17.86421162029705	6.35669441902600
C	-26.01858040823338	16.68596828985478	6.89279952919411
C	-25.03159258579146	16.13723714413096	6.00432482111020
N	-24.82701511440043	17.00613491806543	4.97017463111698
N	-24.00008457309583	18.60365232864888	2.71850306458781
C	-23.45238915225097	19.19346339273599	1.61573835397400
C	-24.11571453358448	20.44571351685075	1.37367280717684
C	-25.08079666129614	20.57502957993866	2.31822603233797
C	-24.99444043242632	19.42049943492528	3.17345290660276
C	-22.45892284086343	18.64422190333176	0.81243706939775
C	-21.91048161506906	17.36991732676884	0.91055131347763
N	-22.24339096789268	16.43338893051668	1.84704421914622
C	-24.45215486990552	14.88058901039725	6.12702150959218

C	-23.59496822759460	14.26445948289130	5.22220034044155
C	-22.36059131727923	13.88111683316691	3.43748230901970
C	-22.42573787550117	12.65189057248766	4.18514574181377
C	-23.16538325261357	12.89591256844323	5.29543175740817
C	-21.58730585065049	15.28044222910133	1.53040220301038
H	-20.53002994735175	17.32072974671335	-0.84761394525935
H	-27.15607770456957	18.55304269598409	6.74699647637239
H	-26.35989511535976	16.21417275547966	7.80094801619272
H	-23.87977449630184	21.12116549932679	0.56615341005091
H	-25.78761838839492	21.38060290802252	2.43698324589301
H	-22.11586122620812	19.25487153846217	-0.01718855770932
H	-24.74917635531514	14.29218376312839	6.98973264804156
H	-23.42324938356889	12.21068811112064	6.08780238356639
C	-29.43316065087958	-19.43916986523987	-4.83371219114394
C	-28.99433102287299	-18.16407552452515	-4.71004405013052
C	-30.04688058589323	-17.42348836992648	-4.07815914318718
C	-29.93374076766520	-16.10899372105638	-3.65325680608867
N	-31.14124720899079	-18.22704401504441	-3.86796212528448
C	-30.75863333445795	-19.47520847329570	-4.29093576054777
C	-31.49682267392965	-20.63056177697140	-4.12648581923194
C	-32.78585212552043	-20.65530582225497	-3.63167856550042
N	-33.55540446606143	-19.54808853151156	-3.37753859914511
C	-34.79824363775991	-20.03819552933426	-3.05628794042393
C	-34.78218056138092	-21.47173555890640	-3.03513355094152
C	-33.53670538459703	-21.85414678649750	-3.40434695499960
C	-35.93499881165811	-19.26467714296945	-2.88221112884128
C	-35.89882868598988	-17.87210427748074	-2.91306031055997
N	-34.76508872473523	-17.10028454679385	-2.90131507773850
C	-35.20842850056192	-15.80918523752839	-2.73921109180554
C	-36.63851667816551	-15.76499611311652	-2.76316136988801
C	-37.06717063822583	-17.04570487445544	-2.86525899216669
C	-34.39710057240166	-14.71307509092349	-2.47563690564605
C	-33.01100564885595	-14.78837253901228	-2.52559914335547
N	-32.29317760062229	-15.85897297727118	-3.00290024403499
C	-30.99329045066771	-15.42452582386080	-3.06165003780767
C	-30.88173919717785	-14.10568615384824	-2.51504977178156
C	-32.13362376216282	-13.71211067632633	-2.18012846918069
C	-58.81152913456323	-16.35263575769635	1.86170199424214
C	-55.99470408473054	-20.73352497314970	-0.41437390337118
C	-54.69876606140284	-20.43179795610503	-0.66556594881501
C	-54.48067737469445	-19.10244936459771	-0.17481065247589
N	-55.64781146903395	-18.57575477627516	0.32312132631021
C	-56.57239357284765	-19.58423455985557	0.21667763740477
C	-57.85264364641888	-19.54664141091520	0.73261079135187
C	-58.41555473101955	-18.42677110981213	1.31219048696387
N	-57.84991638813547	-17.17677538713161	1.32863184994410
C	-59.96350398840877	-17.11194177450881	2.25163499524724
C	-59.72212241148254	-18.39695611759003	1.89929920825947
C	-58.72809978687697	-14.97059212765754	1.92083450556352
C	-57.58545604688497	-14.27663005198289	1.52851527515211
C	-57.50882561113983	-12.84850550055961	1.45870834880523
C	-56.21852809825294	-12.53715608025447	1.18961027667574
C	-55.50489771118407	-13.77447899797878	1.10350253471861
N	-56.36582049083641	-14.83668139667508	1.24535210283936
N	-54.09730741097542	-16.29641140205032	0.62115844546370
C	-53.47585837949171	-15.09302079046861	0.85573612217131
C	-52.05388556971582	-15.25070480896132	0.82913796075185
C	-51.81013485072356	-16.54365173804767	0.50828446971587
C	-53.08342889309780	-17.17776093901999	0.34339429474874
C	-53.24341841459191	-18.47979552750174	-0.12546620763859
C	-54.12326797784806	-13.87297284292528	1.00185219920128
C	-67.45545578230488	1.08442767398723	6.05318614475257
C	-68.83464782237246	2.64447936327226	6.70408277760112
N	-67.50220832302195	2.37924910908801	6.50962913221361
C	-69.07343427954201	6.01456857313622	8.36024624581624
C	-68.56645040741607	4.86325769457560	7.67461880000205
N	-67.23644790070732	5.02377985131676	7.37849413940111
C	-66.91920036901217	6.28667682635084	7.81753810212163
C	-68.04755964613041	6.89250333877519	8.46210741748881
C	-65.71565967454495	6.93059997161924	7.57911659462437
C	-64.65208748077410	6.30504603000749	6.93221585608816
C	-63.44970678605869	6.98106319939646	6.54826176375486
C	-62.60170612825945	6.03646166664325	6.07635381581302
C	-63.28124128572933	4.78162590798405	6.18343979837190
N	-64.56242379868105	4.96668807068062	6.64632672256529
N	-64.75596709208017	2.24989903505851	6.14225698934101
C	-68.77999004397249	0.55920340509611	5.89462511199967
C	-69.63538293258351	1.52563814635831	6.30420943667615
C	-69.34018052022506	3.78228403637143	7.30084936804061
C	-66.29559747975895	0.35254155478715	5.85303733359756
C	-65.02794061324035	0.91432535239526	5.98469587739337

C	-63.81216643487125	0.16606021995587	5.87179841578574
C	-62.79630081139381	1.06110525240860	5.89875637479701
C	-63.39054663949178	2.35643724970276	6.02728551542203
C	-55.91756558471166	21.40125726956374	9.09493571142846
C	-53.54409476080536	18.77416144606155	8.62922690756569
C	-52.74180149112891	19.95546214256931	8.53718704380004
N	-53.49555144866825	21.07124714111096	8.81317890394607
C	-54.77782521914384	20.60994406858390	8.97128503462628
C	-54.80819509274262	19.18027132859548	8.89587007495262
C	-55.85065390179189	22.77442277732946	9.27039931814321
C	-56.99391337037670	23.63198600252296	9.38484738123613
C	-56.52793997094501	24.86557674428494	9.69251198156339
C	-55.09992467863551	24.76555896290761	9.75179767273778
N	-54.69204888438773	23.48977671291883	9.45314429762208
N	-52.15931708757348	24.65201121674784	9.68233292556444
C	-52.89111484900509	25.70246189298657	10.17617153919172
C	-54.26877438000849	25.79284757766090	10.15229066591744
C	-52.02611277389260	26.74642763127400	10.63940884662154
C	-50.75740641355269	26.33826417590090	10.39993197263327
C	-50.84796480239841	25.05196027114848	9.77315194152445
C	-49.76878393996940	24.36178725848210	9.24443816400521
C	-49.90565248340621	23.11138152852683	8.64572626631154
N	-51.03109791356862	22.32774202078263	8.67481410645277
C	-50.64742699492288	21.12458295640739	8.13182353127557
C	-49.29806179954848	21.19569389252862	7.66080879350573
C	-48.83594369990155	22.42692398608762	7.98419556204088
C	-21.90230286939922	4.94828538988642	0.37920552275563
C	-22.91359181726888	4.04756356824347	0.38573583098921
C	-22.41196774918494	2.85939913357021	-0.23412272909977
N	-21.08349145953525	3.00810256705688	-0.55393514240280
C	-20.78073166958274	4.30876089043279	-0.24010546433580
C	-19.59317793567352	4.96601783420292	-0.55379429363011
C	-18.48650202819657	4.29786087596834	-1.05328184267422
C	-17.24838145125057	4.93090590179633	-1.40234212613640
C	-16.37450606558160	3.94116312481805	-1.70288778972487
C	-17.08233279058822	2.70452139096137	-1.55306480630054
N	-18.38623280834182	2.93468625133958	-1.19265923475735
C	-29.90107443375710	26.06617458044325	5.68348498493455
C	-31.22698520381470	25.80358104909979	5.76398941009623
C	-31.36907401345282	24.37731766297591	5.73103527396476
C	-32.56661504980219	23.70650134803649	5.92198824796291
C	-32.65981744146124	22.31783068177973	5.86536782918318
C	-33.85690783460815	21.59013742350837	6.16202798668542
C	-33.61414291917052	20.29344423130500	5.85618256979243
C	-32.27048278053012	20.22907910491465	5.36828949231642
C	-53.37780258030711	-11.80043677332694	2.17939462330586
C	-53.31223650967259	-12.63653245527404	1.06336417002661
C	-52.46475263788128	-12.28532740231593	0.01120061089062
C	-51.70545229972082	-11.12960190639588	0.07338030348347
C	-51.76437173198838	-10.29332649308142	1.19133269735554
C	-52.61295408957474	-10.64882327540881	2.24319488205423
C	-62.68213549645880	3.54911155616781	5.95890986295874
C	-31.69613060936370	19.09223030617265	4.81472754406740
C	-32.97078755679603	15.55327218574589	5.09389622000199
C	-34.12115185425056	15.58903939227234	4.30127314968782
C	-37.12571391532138	13.35319831307740	3.87477042032716
C	-23.19373336658014	1.75650610640258	-0.55272727095949
C	-25.28517043704086	0.91265350731744	0.52638648492760
C	-26.65024713314297	0.98168563053275	0.74489250756945
C	-27.42716261521011	1.96042639228437	0.11942506630588
C	-26.79200428674415	2.87086618101822	-0.72941229482400
C	-25.42608089883181	2.80741536998869	-0.94322867785065
C	-24.65371484059486	1.82581937708489	-0.31976165414967
C	-51.41381986133882	19.96642091474605	8.13063389992427
C	-49.66696276383877	18.19187216940739	8.33902792323818
C	-50.79257075082263	18.69834368884069	7.68696841508719
C	-51.32183892879108	17.98440656396912	6.61048654195691
C	-50.73971851774172	16.79881672718995	6.19648835463462
C	-49.61575161756232	16.28629620165949	6.84980680678833
C	-49.09029907202294	17.00248259605581	7.92863816229291
C	-60.30479240919215	4.01744794683740	6.56408898250360
C	-61.23357351969535	3.50216992567728	5.65818984483835
C	-60.76868620468845	2.93733231203296	4.46938303450598
C	-59.41287867303434	2.89138494782495	4.19380979397073
C	-58.48000026551507	3.40124833191614	5.10072228315417
C	-58.94921423573727	3.96425452457097	6.29059597197579
C	-30.90165568652918	3.32942086834102	0.62902575730190
C	-31.66229193992568	2.16627470771908	0.77538722642040
C	-47.03840455163322	13.65391505595001	6.05262961624727
C	-47.82602753883442	12.59355271281384	5.59626948608629
C	-49.21167468346309	12.76995394310979	5.55382485736513
C	-49.78841946275875	13.96218737206696	5.95388292851498
C	-49.00103096687300	15.02046934649114	6.41505670428083
C	-47.61538164411826	14.84470158420058	6.45651324347174
C	-47.21181612603851	11.32271421704847	5.17669004958282
C	-36.76466261208398	-7.27947316793549	-0.91640236435812
C	-51.44407800444696	-7.91256532475257	1.87139256196363
C	-50.67293230116444	-6.76678012223271	1.95143901111749
C	-50.94806197167432	-9.06966967765221	1.26503789160846
C	-49.65442472828211	-9.03785169307612	0.73779131897014
C	-48.88162287596835	-7.89334312026336	0.82185334058561
C	-49.37589689615985	-6.73740923507934	1.43206932607536
C	-47.25985558539582	8.90732460718247	5.05373000817579
C	-46.03038479669273	8.88938005396248	4.39024467622577
C	-45.40357159053166	10.10920400745516	4.12340890934106
C	-45.98280682801969	11.30419072805511	4.51168072401147
C	-47.84106412809277	10.10253136280610	5.43802497388634
C	-45.50147822467386	6.48771926123364	4.80551169340852
C	-44.91353253619711	5.29205805327592	4.43165025196303
C	-44.21321252722499	5.19089551502241	3.22947786914254
C	-44.11859561476163	6.31546957049925	2.40975804152640
C	-44.70865732746264	7.51054299285243	2.78311843525605
C	-45.40837242757205	7.61663019525940	3.98766755724370
C	-45.10102786806943	-1.93744553465142	2.82430252821146
C	-46.14643313383213	-1.98873799197278	1.89884415797475
C	-46.39407943590795	-0.86239132452664	1.11039575972850
C	-45.62303357419497	0.27926931230044	1.24457677629387
C	-44.32870908650799	-0.79698343899575	2.95751666195617
C	-44.58057139736304	0.32661014953428	2.17009275138568
C	-38.62362867997450	10.77698066375653	4.23323146231650
C	-39.40265918406814	9.64498258019103	4.07259710124348
C	-38.96350957413124	8.57936647360402	3.28283320685420
C	-37.71729184685260	8.68560628768260	2.66027461618896
C	-36.93644262792552	9.81589022218892	2.82436960088334
C	-37.37551090821302	10.88250721291597	3.61344293105033
C	-41.32517002569914	5.73712553020071	4.01625472996928
C	-41.36698758787671	5.07092714465400	2.79141975204325
C	-40.61345022192693	5.56932156188669	1.72870923680089
C	-39.83829899998985	6.70494481042476	1.88694353384107
C	-39.79195698677838	7.37375529581028	3.11267999029636
C	-40.54807306254003	6.87127947402204	4.17462475702587
C	-56.18555318835189	4.37946431960427	5.20698076887229
C	-57.03622970042691	3.34216876524066	4.81577317566767
C	-56.47969186832252	2.24405264309091	4.15475035281084
C	-55.12090369699149	2.18168881230565	3.90167790990450
C	-54.26941498092950	3.21705419335484	4.29652721443246
C	-54.82751273964156	4.31890481779033	4.95031801626037
C	-52.82126895825572	3.14668552419089	4.03925361892019
C	-52.09265536441950	4.29430957054501	3.71505140834016
C	-52.13790765848091	1.93039548853787	4.12143729626270
C	-50.77467491215599	1.86522116837382	3.89479761490024
C	-50.04629898720172	3.01340949107268	3.57348313588221
C	-50.73010333221684	4.22852888259629	3.48412711402716
C	-48.59369675290560	2.94557557849372	3.34079813906890
C	-47.78887080483820	2.09323071311040	4.10090525625701
C	-47.98799407562562	3.73411062638157	2.35941503227434
C	-46.62172140728409	3.67419524607505	2.14659350917780
C	-46.42300032101369	2.03129716951862	3.88697313558311
C	-45.82166019849262	2.82193810483256	2.90767434582759
C	-44.35762888904460	2.75953954054182	2.68185274584538
C	-43.75556999905810	1.54933856530370	2.32019439271129
C	-42.37352428692701	1.49110494151059	2.10909528070446
C	-41.59356787015372	2.64300829820275	2.26006516708519
C	-42.19529525539616	3.85264291459614	2.62408395542220
C	-43.57733011983080	3.91091904333784	2.83500082429997
C	-40.12879193896850	2.57923583140515	2.03951302825265
C	-39.61330441129289	2.18243888640643	0.80562558850127
C	-38.24593891374206	2.11880228120052	0.60151165354038
C	-37.35427007256148	2.44780289706784	1.62550743801249
C	-37.87449089822145	2.84534435029618	2.85966143318203
C	-39.24176958641557	2.91112061011767	3.06364070862364
C	-35.89932998351740	2.37577213362282	1.40927824575509
C	-35.34084770638345	1.35910497753341	0.63045167256785
C	-33.97388490672868	1.28988080536799	0.42874470695854
C	-35.04256051880025	3.32158444525832	1.97797528975955
C	-33.67600099748891	3.25488956144439	1.77245132626899
C	-33.11662994692575	2.23738091028965	0.99476723334717
C	-46.97028212754384	-3.20198697164661	1.76429903881591
C	-48.34448943068770	-3.11276166273074	1.52860413170472
C	-49.12061952009554	-4.25287909711561	1.41853155299866
C	-48.54954899804391	-5.52299721952188	1.53522991502832
C	-47.17346516454067	-5.61161263831032	1.76232779034962

C	-46.39812484118460	-4.47163188470991	1.87713806085319
C	-41.73519650171080	0.20749167513568	1.73079585303017
C	-42.11733188175459	-0.46075842282609	0.56756729905767
C	-40.74297843295458	-0.35467300120491	2.53416853320773
C	-40.14935545411411	-1.55471322821328	2.18384205616000
C	-41.52237220460862	-1.66019620815861	0.21688823241432
C	-40.52939498310690	-2.22700987442957	1.01964127565061
C	-39.89883898346263	-3.50540366461218	0.64945758351405
C	-40.64823102900946	-4.53188016957171	0.06919786131499
C	-40.05741478794491	-5.73559379016172	-0.27171021758697
C	-38.69510049602559	-5.95389121386920	-0.04952666035958
C	-37.94511290627264	-4.92506384296890	0.52661480720221
C	-38.53692147233410	-3.72324951316991	0.87242227047300
C	-38.06652777642809	-7.23572828835143	-0.41041780532210
C	-38.75602472007277	-8.44104831827099	-0.25349015347763
C	-38.16399804735393	-9.64624098190865	-0.58668821093372
C	-36.86264013282754	-9.68966547942566	-1.09373844412566
C	-36.17437944792928	-8.48428107433834	-1.25436313273083
C	-31.00637955534021	0.93426042186734	0.70580471160695
C	-29.64019673248839	0.86780898690761	0.49785801513626
C	-28.88049500871379	2.03089647790835	0.34747542654719
C	-29.53618400887812	3.26291816422199	0.41682796265143
C	-36.23268592091376	-10.97298433175141	-1.44798611284419
C	-35.38622732345370	-11.07397092216960	-2.55522614127500
C	-34.79137904849647	-12.27926792178182	-2.88578544752916
C	-35.03037907736259	-13.42315325719290	-2.12208162500121
C	-35.87770378938516	-13.32644701003133	-1.01679502251531
C	-36.46682547223836	-12.11917868823539	-0.68367879036293
C	-36.54432618601477	12.08534627860377	3.78822667265806
C	-35.15271562174883	11.98918224564157	3.87286369553420
C	-34.37159711417614	13.11872350279975	4.04004951510582
C	-34.95303731973303	14.38654792637383	4.12486839255776
C	-36.34435300853857	14.48318549865266	4.03808904794116
C	-34.46284536676971	16.79337590725035	3.68025276962215
C	-33.68321383687311	17.92458721502303	3.84851201448517
C	-32.53246459878864	17.88385018288682	4.63762128272985
C	-32.18611702048806	16.68173205654129	5.25692463602180
C	-30.36393663140463	19.04875913140524	4.42501649641217
C	-29.74861873438782	17.92249855173840	3.79204221743788
C	-28.42226542926979	18.18786878113614	3.72646565137954
C	-28.22793831427834	19.48008739484385	4.31196094396549
C	-26.97437200301876	20.04227669588218	4.54213221833927
C	-26.81743661868488	21.34047439962719	5.00083261972502
C	-25.55005449663224	21.96121385710966	5.25386056116759
C	-25.80119030128110	23.26167411211290	5.53531470812225
C	-27.22238725626793	23.43176847516782	5.47217636547826
C	-27.86183826565409	24.64892912317891	5.59951250337388
C	-29.23402621841733	24.80187912310730	5.58663277462740
N	-30.14395311226324	23.77471381538539	5.57770048895709
N	-27.83928628168223	22.24109758250017	5.18131519487885
N	-31.67334089882593	21.46506398037101	5.43914079677412
N	-29.43132312753567	20.02588820677898	4.68011900654589
C	-22.65278572722893	0.59242309893069	-1.08272179378619
N	-21.30945388290598	0.32387692244765	-1.19695342925516
C	-21.23572386408386	-0.99570739135264	-1.56405175912836
C	-22.54678970099619	-1.52897464745564	-1.78117137512990
C	-23.42596582896664	-0.54168459523781	-1.48730611147106
N	-18.55423697160776	0.15681270311242	-1.46509340036341
C	-17.18983813180396	0.27028642048241	-1.55705674066980
C	-16.49607329486831	1.45932380731947	-1.66451487691051
C	-16.57930384395388	-1.02411157278834	-1.62451233741300
C	-18.80958675357007	-1.19129638003243	-1.53736328221188
C	-17.58365977751465	-1.93200209743025	-1.60132282448627
C	-20.07100565213667	-1.75655744234531	-1.63639341370853
H	-28.92014713296127	-20.29236016049107	-5.24854711811147
H	-28.04470031168755	-17.74526551675497	-5.00235732480021
H	-31.03589530706216	-21.57342169502242	-4.39704476049719
H	-35.63022380334018	-22.08744082899495	-2.78146019723936
H	-33.14303879085239	-22.85172899114902	-3.51865873341617
H	-37.22933817029866	-14.86496007670498	-2.71261033070383
H	-38.08146986172113	-17.40815608872674	-2.91181331107862
H	-29.95845301288611	-13.56080336796731	-2.40130163409288
H	-32.44411342088911	-12.78182127305450	-1.73307142052176
H	-56.52864642377464	-21.64632181981570	-0.62629556391945
H	-53.94048960193121	-21.04265658415470	-1.12872845184689
H	-58.44900880367602	-20.45031392136432	0.68392647020819
H	-60.83957227380297	-16.69334568115045	2.72058389710657
H	-60.35800188760403	-19.26006574736112	2.01723596160769
H	-58.34287610169247	-12.17867442925811	1.59299879911846
H	-55.78077156620097	-11.56145553070335	1.05517447514402
H	-51.34206562623888	-14.46852255671012	1.03695364862419
H	-50.85698173026708	-17.03482344544146	0.39629052100952
H	-70.08768201716630	6.11770878459606	8.71220602745779
H	-68.03858257509825	7.87126913184828	8.91438037196712
H	-63.28827927966562	8.04421634125228	6.62592167020499
H	-61.60567258639423	6.16773138519475	5.68600765079118
H	-69.00357545003897	-0.42863281223507	5.52467330345835
H	-70.71285401869608	1.50084393868412	6.34466017445291
H	-70.40503147126127	3.82455600162147	7.49821047298705
H	-63.75341349262136	-0.90709743576547	5.78736041269970
H	-61.73679102173635	0.87075093518024	5.84501755408719
H	-53.17689339837922	17.76785116312805	8.51009895881968
H	-55.68821691163146	18.57346781553004	9.03601481270626
H	-58.01416299671278	23.30841306510818	9.25433187837925
H	-57.08404978258702	25.77248587542706	9.86987357724804
H	-54.72454865859167	26.72354399852213	10.46964794801653
H	-52.36601803020215	27.66697840974182	11.08690784434278
H	-49.83212229492377	26.85112487129840	10.60838867035425
H	-48.78145084666224	20.40223582035550	7.14580445313134
H	-47.86168634799786	22.84606865923654	7.79060909442451
H	-21.90404207236904	5.95611006657144	0.76208034866437
H	-23.91061434526351	4.16538492017224	0.77785072173677
H	-17.08660587372245	5.99695551154737	-1.39907615160200
H	-15.34042995303938	4.02043369085833	-1.99887156375224
H	-29.40033952268066	27.02138144141809	5.69018861102334
H	-32.04889135497361	26.49619100142841	5.85008463412941
H	-34.76059724858934	22.02411667806881	6.55864989575920
H	-34.27622617217040	19.44862817328643	5.95427313346428
H	-54.01779584172346	-12.07015752530352	3.01227284239992
H	-52.41795666826735	-12.91513864309882	-0.87037738128838
H	-51.07357149883761	-10.86413199194489	-0.76703765105134
H	-52.65947683105765	-10.02984835097909	3.13241628399527
H	-32.69643743129003	14.63805124987874	5.60684928258486
H	-38.20216915946290	13.46001359674812	3.79627706965560
H	-24.69509057520272	0.15658450663016	1.03254116119055
H	-27.11438868678835	0.27813768680359	1.42726537059699
H	-27.37521771121142	3.62518463270660	-1.24601539183018
H	-24.95191291382920	3.51212803681561	-1.61744897482937
H	-49.25600142156130	18.72606824408332	9.18860010061180
H	-52.18477356298061	18.37523140218887	6.08263130224465
H	-51.15278678518055	16.27552784476756	5.34116719302983
H	-48.23325209193539	16.61480323902776	8.46826205102031
H	-60.65026287028534	4.44207090940513	7.50023279167020
H	-61.47964186700091	2.54949529107458	3.74816578220513
H	-59.07694716247602	2.47100356810645	3.25228006901640
H	-58.24534395346032	4.34517741454294	7.02234771269399
H	-31.38614762096195	4.29916637093341	0.66345102108845
H	-45.96132253957845	13.54042927117359	6.11046127797916
H	-49.84674559837249	11.97039966935708	5.18813795340061
H	-50.86771711885932	14.06527250201196	5.92462046170424
H	-46.97832500599691	15.65503822564557	6.79396371826050
H	-36.21166132799451	-6.35817279196252	-1.06384472416510
H	-52.45155562823747	-7.90303527160993	2.27295511533374
H	-51.07772942588124	-5.88854638416172	2.44253068958561
H	-49.23900517675880	-9.92553087639779	0.27321853581761
H	-47.88506767066547	-7.89389878420981	0.39382208557524
H	-47.77614061811223	7.97558696327200	5.25823663317958
H	-44.44168885813909	10.12312163447787	3.62229374864298
H	-45.48000229940757	12.23694029358206	4.28038212005657
H	-48.78853657142387	10.08582042513767	5.96560263611123
H	-46.02078481297653	6.55006945004960	5.75547452121128
H	-44.98728942751085	4.42843112076608	5.08313212658309
H	-43.58535738719218	6.24880339025385	1.46797427445600
H	-44.64082610164813	8.36633235492831	2.12050935826945
H	-44.90267741378081	-2.79097323257193	3.46315821540625
H	-47.18593110722784	-0.88482127812056	0.36978586834120
H	-45.82418187686841	1.14204771258872	0.61936140783483
H	-43.52753573410247	-0.77093729159900	3.68768901977897
H	-38.98307335996546	11.58277642038532	4.86395912756893
H	-40.37323968519581	9.59531833668540	4.55428483264056
H	-37.34551004272186	7.86660808404926	2.05398590262778
H	-35.97922333115900	9.87706459500178	2.31809982663117
H	-41.89862837132920	5.35553691762692	4.85366664057681
H	-40.64365880988595	5.06684552973996	0.76831920997781
H	-39.27773232506289	7.08754523733926	1.04113525663545
H	-40.51229720141663	7.35979731429057	5.14213282917130
H	-56.59258161226127	5.25244191927531	5.70556401409330
H	-57.11413251458274	1.41830865276095	3.85142155447920
H	-54.71847516321412	1.32239218390155	3.37632487034911
H	-54.18901717625067	5.13106488559916	5.28052529121054
H	-52.59986736863219	5.24892943317228	3.62693963055833
H	-52.67534747707001	1.02599717183056	4.38515244272831

H	-50.27161084122701	0.90605794470117	3.95348599415548
H	-50.18650391385947	5.13742454581085	3.24976127441576
H	-48.23193273009142	1.48706998566256	4.88337940909060
H	-48.59377723899020	4.38768498754505	1.74144774683872
H	-46.16891807760429	4.28770411583097	1.37566048416007
H	-45.81209789324880	1.37109926621950	4.49248333791454
H	-40.29118903424067	1.93071100499156	-0.00236276828870
H	-37.86707623795697	1.82871572606776	-0.37230645035115
H	-37.20329098729686	3.08546008101735	3.67687999800720
H	-39.62842718766127	3.21245740657570	4.03080691368740
H	-35.98183475538486	0.60272914703266	0.19051142722488
H	-33.56892099202316	0.49821491172449	-0.19221717561017
H	-35.45133427195987	4.13002450934998	2.57450531720999
H	-33.03314901875316	3.99444655339011	2.23734432639538
H	-48.81610894002853	-2.13915400358551	1.44987308181625
H	-50.18311397379882	-4.15446313915921	1.22421717416256
H	-46.70579204322670	-6.58415897062541	1.87074130572752
H	-45.33056043560838	-4.56949168145493	2.04216217218261
H	-42.88072545102268	-0.03077245600767	-0.07124298389778
H	-40.44281457760382	0.14888291362343	3.44633010678876
H	-39.39617154554697	-1.98496537020909	2.83469729907766
H	-41.71053538801893	-4.39404673524854	-0.10120871734845
H	-40.66012996521607	-6.51063564770695	-0.73255810919172
H	-36.88890444549195	-5.07250116607736	0.72416222404469
H	-37.92896245204264	-2.93691840879524	1.30670114585276
H	-39.76220861993708	-8.43850542851289	0.15138317101132
H	-38.72740157972626	-10.56524324592832	-0.46677666124206
H	-35.15754552166760	-8.48960527684154	-1.63172202598937
H	-31.56860648151589	0.01546203658556	0.83246619338770
H	-29.16000242330112	-0.10258466766363	0.43368315980912
H	-28.96907529802003	4.18237225797611	0.32013774318747
H	-35.20665485657074	-10.20504825391424	-3.17874506208552
H	-34.15014337376355	-12.34339967808064	-3.75799047882170
H	-36.05698846282663	-14.20205775226807	-0.40263226065535
H	-37.10011683862330	-12.06243367441904	0.19481793459434
H	-34.67484211790247	11.01673374312839	3.82487461688564
H	-33.29303204644874	13.01517101281298	4.08831905391598
H	-36.82404386670748	15.45277639060847	4.11651768171854
H	-35.33715138889349	16.84227147522254	3.04055120620459
H	-33.95472092826926	18.84687362051603	3.34681608733251
H	-31.30533125604497	16.64014963960131	5.88820159864961
H	-30.27313054256312	17.04838418710122	3.44205363691029
H	-27.63832375985471	17.57312145164513	3.31433247058439
H	-24.60097812260281	21.45190157698845	5.20395338279035
H	-25.10196230842697	24.04970361164076	5.76616982669900
H	-27.25084720052731	25.53601297337002	5.71958811021677
H	-22.75698740617322	-2.53007213734734	-2.12169086761262
H	-24.50212358978969	-0.56770776070785	-1.54108577480317
H	-15.42849800193366	1.41248094649933	-1.84544206739194
H	-15.51602007379923	-1.19498550584595	-1.68312700411882
H	-17.52258241456954	-3.00795144656501	-1.63655811603255
H	-41.81947022929983	-2.15189828040762	-0.70288936752646
C	-20.43864430637434	7.17529766488114	-1.17885504898569
C	-22.78521787712712	13.03611352348528	-0.26018692285708
C	-21.71627755666583	12.37789009356347	0.44572159444678
N	-21.42050300512135	11.20819962948573	-0.19150208861312
C	-22.24779526590691	11.10513453613808	-1.27284343566544
C	-23.09452522057215	12.26376665332735	-1.33126294988654
C	-21.08720049100775	12.85077545627258	1.60616174817322
C	-19.28907276274009	12.77029351652875	3.37167275032186
C	-20.02052442160699	12.22171906724753	2.26036441447172
N	-19.50501679552824	10.99782662036566	1.95005335260434
C	-18.34578473563813	11.85880631758529	3.71903192532413
C	-18.50007439234171	10.73483629357881	2.83651585317964
N	-20.76805961920698	8.48832591401452	-1.02670434702456
N	-18.86303555996036	8.26633111168651	1.12416170027900
C	-17.99883966670333	8.38748452880289	2.17411186672004
C	-17.37692168239598	7.11502506307285	2.42069735248334
C	-17.90486679234855	6.23449557447015	1.53400286536418
C	-18.82857525683782	6.96686830246923	0.70802590129066
C	-19.58481449863457	6.43949568728555	-0.34422529711126
C	-21.64614624284409	8.81178574661145	-2.02208450570251
C	-22.31627239306432	10.02269696115835	-2.14277110650782
C	-17.79931369108790	9.53718269871457	2.93064410457241
C	-21.84512956254717	7.66586122319646	-2.86577170338235
C	-21.11134734623374	6.65352463712247	-2.33974874076299
H	-23.84420776799067	12.44166824836037	-2.08638350839829
H	-17.61315035558095	11.92575642077015	4.50814511188421
H	-16.64738440655438	6.92582649354694	3.19268762990854
H	-17.68814262785862	5.18278390360618	1.43616541304232
H	-23.01516716248220	10.10948213231312	-2.96904317819769
H	-17.05622358390554	9.47339187999995	3.71970121372858
H	-22.47874779705313	7.64637422378975	-3.73867860432738
H	-21.01768419197843	5.64336412238151	-2.70541892480996
C	-34.97209305844939	26.09339585506197	9.11549330960222
C	-40.28237744692967	26.10748956419764	7.85852448274516
C	-39.49896036612369	27.05317350252023	10.06048457290892
C	-39.27729481742860	26.46452449460995	8.76615146882468
N	-37.93595007094898	26.31579308030332	8.56966749388199
C	-37.30109295751941	26.75579214076818	9.69583799748662
C	-38.28100578451566	27.24246778990379	10.62796450094459
C	-40.06624445855991	25.65490373515104	6.54914042807050
C	-41.09104128226709	25.32593032538766	5.59217987614457
C	-40.46665687646266	24.94905364492843	4.44872114696479
C	-39.05417767823595	25.00388192458844	4.70308953446769
N	-38.84491768488087	25.44225840806430	5.97917384766585
N	-35.18935312612135	25.60553865837444	7.85902389003278
C	-35.93506026573831	26.66429299942176	9.94055768781391
C	-33.60164152618693	25.85854424321045	9.48081148072382
C	-33.02108382595618	25.19791674861067	8.44794294085674
C	-34.01980863900818	25.05458117688742	7.42139196283713
C	-38.06696042212135	24.60319718548417	3.80977843328675
C	-36.70908379869498	24.47048996358501	4.07159708048483
N	-36.10164765860567	24.74768148361683	5.26345001147270
C	-34.80044595676893	24.35712559146507	5.16304256643653
C	-34.56560315518038	23.84035009379861	3.84010304867952
C	-35.73890200408702	23.92246859801464	3.16437204151263
C	-33.83667549265920	24.43901613657397	6.17849230178807
H	-38.05477978374824	27.66236496268348	11.59566568754433
H	-40.91346033187848	24.63653306704963	3.51782020268265
H	-35.59236380689343	27.02412289418750	10.90592935636923
H	-33.15727798515358	26.14630898176011	10.42087683607471
H	-32.00560728153644	24.84252402585462	8.37616251213991
H	-38.40177126089778	24.30151545835804	2.82205326896395
H	-33.61705143022074	23.47550315457221	3.47946022948573
H	-35.94428385465270	23.62865084299155	2.14686993558337
C	-67.12977375190039	-3.95809345297829	7.39829721864759
C	-67.14574726429106	-5.32192561052666	7.66964242187561
C	-65.82865547775624	-8.36208586219078	6.29508501264666
C	-66.53246706268597	-7.72390448988244	7.26392182032688
C	-66.60508465002826	-6.33819646160087	6.88915901426509
N	-65.97915698731157	-6.16723171606168	5.68753196984355
C	-65.47769536060608	-7.37687528905773	5.30657370458800
C	-64.74191601970271	-7.62827565161073	4.14164839374511
C	-64.51005186519173	-6.70115720576018	3.11566813401068
C	-63.79342482701487	-6.95957067840822	1.89345083399200
C	-63.80936620432640	-5.81339167715587	1.16857766542241
C	-64.49974907168198	-4.82867526839585	1.95380047197098
N	-64.91830524760924	-5.39920113010942	3.12155957628206
N	-66.66100827288481	-3.38046623856406	6.25349708969338
C	-67.53955373761050	-2.92656262308096	8.31194254457854
C	-67.27678460144985	-1.73725111564046	7.71464148342158
C	-66.73827060422323	-2.02701115745914	6.41162643735249
C	-66.34134697433507	-1.08212931370594	5.45921106717398
C	-65.87467722266038	-1.37565246893302	4.16982565820990
N	-65.61243957377633	-2.61926469339355	3.67942800195472
C	-64.64929096059164	-3.49136607978717	1.60382061939130
C	-65.14747874184029	-2.47040104701041	2.40352171998032
C	-65.15898846315019	-1.07583108873269	2.05746912015404
C	-65.59458481352785	-0.39942703419104	3.14961153209574
H	-67.57368385461261	-5.62015177359982	8.62189719136743
H	-66.95900766619876	-8.14168290233898	8.16251044673016
H	-63.37416096118718	-7.92630896838551	1.65005133093040
H	-67.95287625165581	-3.10189555856939	9.29296858153758
H	-67.43752645496640	-0.74736008784604	8.11077917150669
H	-64.27850425241202	-3.20308120051960	0.62498365883465
H	-64.85664984379328	-0.67585833563027	1.10225240079663
H	-65.72968844729698	0.66474297822784	3.26018727098692
N	-63.82530242442769	-10.93820554998688	2.58035829073686
C	-59.85405451730927	-14.14152936994912	2.43063034020039
C	-61.00492383889435	-14.00854382797506	1.64641261948843
C	-61.29150024927648	-14.73472301853050	0.43711692769090
C	-62.50638635202928	-14.31884822409481	-0.00035873598566
C	-62.96081233640662	-13.30623308679577	0.91334066764233
N	-62.03472529166015	-13.15061862597589	1.90432462650990
N	-60.39009431580974	-12.48968025360951	4.17656099928130
C	-59.61114622059195	-13.46824162241893	3.63681547713050
C	-58.48663150557621	-13.70546331286051	4.50382919304801
C	-58.60429806652641	-12.85468267762506	5.55370687844146
C	-59.78993728921041	-12.07409895508155	5.33136738315579
C	-60.20370934594369	-11.00348274981086	6.11395064488651
C	-61.26871096606865	-10.14642821175173	5.86040699226803

N	-62.16800547776637	-10.27172823377601	4.84063831685992
C	-64.12568878450202	-12.56052436960777	0.76860832856880
C	-64.51876105702216	-11.46343719211296	1.52769114447718
C	-64.46746908243639	-9.80066715309679	2.97239183425590
C	-65.63530712678725	-9.61534610660307	2.15216558613781
C	-65.67214336342991	-10.64544630894553	1.26967547704545
C	-64.06350215130787	-8.94756544979348	4.00695895642153
C	-63.00094514268657	-9.19211238536563	4.88859802066027
C	-61.55076570236088	-8.94386170580117	6.59321680569515
C	-62.60149282523419	-8.34314526741645	5.98127975424962
H	-60.64376935920135	-15.47220518972010	-0.00919925865004
H	-63.04635268518340	-14.64532730406899	-0.87547954672397
H	-57.71705713548319	-14.44051979944133	4.32961658787578
H	-57.94618103798625	-12.74710578422000	6.40171917916196
H	-59.59359550682193	-10.77345429645131	6.98202755968617
H	-64.76963445619896	-12.82713269667383	-0.06393036409916
H	-66.40387515189059	-10.83823067713934	0.50068959238745
H	-60.99374121582303	-8.59977377068980	7.45065714659502
C	-45.48504827368681	-20.91869752534894	-1.83529384675506
C	-46.07042592197097	-19.99463924978305	-2.71253486987062
C	-45.37812471706552	-19.23895327644031	-3.72440138452855
C	-46.30363316006542	-18.48040166681913	-4.36271451873561
C	-47.56562055604012	-18.73115370192001	-3.72454700366880
N	-47.39228082249269	-19.65709330201517	-2.73613977367598
C	-46.17644061355840	-21.67801739908757	-0.88281721172902
C	-45.60004823370729	-22.72032842152314	-0.07512030597227
C	-46.58793432242240	-23.20308640238406	0.72005007632939
C	-47.77556209966604	-22.45070334072932	0.42116816371485
N	-47.49829439530872	-21.55419092029147	-0.57098199119844
C	-48.98578084218460	-22.55707067701416	1.09815865316137
C	-50.11849577680417	-21.77041820913031	0.91902086087997
N	-50.27569964852492	-20.80841421419644	-0.03687663953427
N	-50.17019921312665	-18.92582460751489	-2.21590184826517
C	-49.95101980218051	-18.15546802351634	-3.32266793843197
C	-48.75521128144462	-18.07730234440590	-4.02544272481692
C	-51.11967115408429	-17.36102307415804	-3.58284588886294
C	-52.01372872698941	-17.63674219919401	-2.60069265364241
C	-51.40905784549294	-18.61919797467361	-1.73954695214801
C	-52.00804946984791	-19.16442176299923	-0.59458549872062
C	-51.48404054020944	-20.20967472640952	0.17373407062904
C	-51.28505749334743	-21.79414623635686	1.75881952527284
C	-52.11568836083524	-20.81757286678540	1.31538830222635
H	-46.15182281821244	-17.79079670729170	-5.17836045534831
H	-46.52669443830519	-23.99020226896249	1.45532724578039
H	-49.03097347490402	-23.29520993906684	1.89312683970185
H	-48.72835860588249	-17.38393414208536	-4.86044772019338
H	-51.22572511365967	-16.67314992257843	-4.40710417437880
H	-53.00213367879579	-17.22588600565369	-2.46966545872069
H	-51.42890997614509	-22.46212176254122	2.59374824314413
H	-53.07567326928240	-20.53273630381455	1.71523897256311
C	-23.68773050272264	-10.63693115332249	-3.39022964954152
C	-22.34727363194439	-12.14815256301507	-1.88147705730274
C	-23.47294237272727	-11.85217979555438	-2.72775491718676
C	-24.71103721971193	-10.39103610420031	-4.31672256232260
C	-24.91658061001798	-9.16156935844923	-5.03806835983850
C	-22.51008667894041	-13.41664784775406	-1.42790184267319
C	-23.74559535952500	-13.90529731919709	-1.97615162710913
N	-24.28922315508624	-12.94346032097713	-2.77884398110043
C	-25.99423939342051	-9.34265622454293	-5.84138172930352
C	-26.48601624984049	-10.66898360106111	-5.59276283734403
N	-25.68362139201141	-11.27960592513378	-4.67213907796447
N	-26.41142312003442	-14.94802800184700	-2.95794746563140
N	-27.79216861932227	-13.28895765164374	-4.86663691377962
C	-27.63843818956820	-11.21008067513703	-6.15169635489565
C	-28.24842670892236	-12.40988609486871	-5.80767453284307
C	-29.51310358680718	-12.87148661853715	-6.30975484196753
C	-29.81968862540653	-14.00519346055519	-5.63099706028870
C	-28.73503218854987	-14.26105389587340	-4.71964207519239
C	-28.67131748022510	-15.34033828329849	-3.82606643841825
C	-27.56723884085472	-15.67018698853290	-3.03243676110362
C	-27.46611843075133	-16.80799595633665	-2.15659054962185
C	-26.23533838848888	-16.76628735538331	-1.58763097473259
C	-25.58326699734944	-15.58232790876909	-2.07717050508272
C	-24.33680028896860	-15.12477685960484	-1.66537223983789
H	-21.86387432722461	-13.97738375198252	-0.77069895893355
H	-26.43956462634129	-8.63877482975452	-6.52703419202681
H	-28.15054173733148	-10.60030690727029	-6.88969715845642
H	-30.09029085307300	-12.37547657332080	-7.07437760704215
H	-30.69414284697548	-14.62680234718696	-5.73980405606161
H	-28.23963776712277	-17.54424976223220	-2.00764293934955
H	-25.80532621501706	-17.45786364700847	-0.87996001094675
H	-23.79245361571466	-15.76254593561285	-0.97394098162400
C	-62.15387838540222	15.91455107845115	8.86873534380739
C	-61.49529148498780	16.17959169123208	7.65964900090922
C	-61.62095170437096	15.41329679213261	6.44674432662857
C	-60.83855971638498	16.00887589864668	5.51262044128231
C	-60.19370029166394	17.12182709279647	6.15164135605362
N	-60.61828857025257	17.20299563634692	7.44680079414625
C	-62.05646880895775	16.69278979098527	10.02915496302514
C	-62.83237002632467	16.49908893853666	11.22556199828829
C	-62.44011473867646	17.44610756945839	12.11470608489652
C	-61.40752428006521	18.21865120951918	11.48003895966809
N	-61.21822012704644	17.75316627812323	10.21021708226391
N	-59.11694403588188	19.78623947051103	10.27484621954109
C	-59.57669428725919	19.90446872289499	11.55491845400398
C	-60.65931681592570	19.21765542704486	12.09357614965202
C	-58.71904193197336	20.80035318429206	12.28189810750641
N	-58.53235416163950	19.24426501136744	7.50559455838384
C	-59.23625154766926	17.93693247815064	5.55824565756692
C	-58.46030255416815	18.90434954617124	6.18436993343572
C	-57.52111618334276	20.12053663470220	7.76110084965715
C	-57.99395607890599	20.55433581277589	10.16615659194380
C	-57.22246675305954	20.69093440981960	9.00701810970496
C	-57.73086775443114	21.18203472018243	11.43449211368371
C	-57.38494955292531	19.63174768655452	5.56861505595065
C	-56.79771846608280	20.37169848359617	6.54207429726063
H	-60.68567001826843	15.71450783007623	4.48604579766513
H	-62.80215064425126	17.60896240835039	13.11787159038061
H	-60.90980763255382	19.44358117537276	13.12559880302859
H	-58.85253926005593	21.07644477036099	13.31627720301061
H	-59.02847383823537	17.75390366172612	4.50846235107763
H	-56.89848014248827	21.83597159765825	11.63971413366828
H	-57.11436797660090	19.56594856726571	4.52636853647174
H	-55.95695843773326	21.04070661786213	6.45002539149445
C	-65.47932538832789	8.33276783345744	8.01906123937147
C	-66.12849688945248	9.37238668477455	7.34473382057271
C	-67.15123101729856	9.22054201022913	6.34334900069608
C	-67.51853982527716	10.46739232587541	5.95508479880391
C	-66.69935030282754	11.39606978399037	6.68534164483620
N	-65.87726791221020	10.70176188862165	7.52557047006333
C	-64.52191173737434	8.49411792687482	9.03108807215937
C	-63.95190923157600	7.42423030517465	9.80757577476896
C	-63.06569942467223	7.98315211332266	10.66902909474207
C	-63.06853309085075	9.39756214938406	10.41610249078051
N	-63.98040384645008	9.67613114551601	9.43771436406834
C	-66.70148089281888	12.77578895376054	6.51113654169563
C	-65.81718522689688	13.69286187261594	7.06907788295845
N	-64.79188919529716	13.38988338360172	7.91871991150053
C	-64.08358897802974	14.53477645971349	8.13703904308369
C	-64.70637694395948	15.61530441579298	7.41900738726626
C	-65.77998970613039	15.09824830731077	6.76996836390111
N	-62.88752380335504	12.35622084918721	9.81755627429354
C	-62.09668312038983	11.66933977500323	10.69344478406854
C	-61.07473097286891	12.54573484548519	11.19369688687776
C	-61.24690378095532	13.74505494983796	10.58435482267969
C	-62.40200619979563	13.62845768702106	9.73202867449510
C	-62.21126639152796	10.31904697893418	11.00415327350429
C	-62.93665838062872	14.64881518773635	8.93253360303843
H	-68.26115968124901	10.74507308039170	5.22337238377534
H	-64.21228312498734	6.38251134514626	9.70788855256480
H	-62.44984836444898	7.49041117178378	11.40506389929485
H	-67.43715403324758	13.17418755796633	5.81908628292095
H	-66.48435824005337	15.61093950629979	6.13361061696943
H	-60.31239770999814	12.26208502085982	11.90240186533519
H	-61.51407064130636	9.93111688885099	11.74045015552130
H	-24.43516076245821	-10.41791369436670	-0.80734675171354
H	-22.10760328362159	-10.47783330655673	-5.61595194734284
H	-44.98259957364224	-19.96297805256318	0.63836957873379
H	-45.15099237109144	-22.35921273416571	-4.13405958497053
H	-41.21747012279718	28.33116524245289	6.57362497754232
H	-40.05003961339089	24.20449182173395	9.75833269757578
H	-20.14421267455749	14.76659894210744	-0.09914835522452
H	-21.96104214494555	11.72272009787835	3.89692636518760
H	-23.23737692731338	13.97173271885942	0.02710659265880
H	-19.47685155137360	13.73605343274715	3.81381080115676
H	-40.46819075586608	27.29548942020856	10.46707950707121
H	-42.15244452416106	25.38060111430679	5.77329001682736
H	-65.57194886559073	-9.40815175655659	6.23774921396964
H	-63.34004634272517	-7.90012725884413	1.62481301274892
H	-66.33560077587524	-8.80151921078904	2.25567215865083
H	-63.06905352716322	-7.40859206120138	6.24655307253949
H	-44.31931837108094	-19.28697797436412	-3.92179228923489

H	-44.57105996465949	-23.03956487771312	-0.12707237375705
H	-21.53665052070362	-11.46658628065686	-1.67703958776602
H	-24.30996138605535	-8.27536755898704	-4.94317224290608
H	-62.23342925548045	14.53420203452414	6.32664156522707
H	-63.58593726381105	15.73783404399165	11.35231448892051
H	-64.36163626584663	16.63732394311682	7.42718269061360
H	-60.64740724810562	14.63334603458564	10.70229022031372
H	-67.53807101846913	8.27690328350920	5.99326323859540
Zn	-21.30323187405548	-6.46536147107721	-2.31734937964393
Zn	-40.57589575896981	-20.70550900442902	-2.14456266069382
Zn	-45.04501289027974	25.70089569806969	9.03459738194217
Zn	-23.52808257030201	16.72531130398758	3.41320202611027
Ni	-32.93859278836798	-17.68377200844017	-3.28780176174981
Ni	-55.99039103185481	-16.72161499266493	0.87915232911235
Ni	-66.01465918769749	3.65491643943961	6.66846780535264
Ni	-52.84468429069445	22.88545148110344	9.15576384950551
Ni	-19.83307423109838	1.60585779127307	-1.10216910597012
Ni	-29.77189877426181	21.87699673650388	5.21929450510685
Zn	-20.12864273923090	9.74520744294786	0.45637523924858
Zn	-37.01584626941268	25.54109457792915	6.91388066484877
Zn	-65.80563070070372	-4.39399817217952	4.68064005047488
Zn	-62.11271563204924	-11.72105601980096	3.38269294003773
Zn	-48.83824337428241	-20.24689801993162	-1.39816740084731
Zn	-26.03638468061871	-13.12188215912833	-3.82851146935274
Zn	-59.88119411942086	18.50641944706775	8.85653984273110
Zn	-64.39394761347477	11.53379902345051	8.68431875212558

Partially fused nanoring **1**

total E = -1475.532166450853 Ha

C	-17.56612800300077	16.02643304708771	-1.29744075117534
C	-21.98977726139325	8.59564837442950	-3.39580201626077
C	-21.70174948259258	9.56243645732364	-2.46733531845196
C	-21.95083957728876	9.75298387379072	0.01828867871525
N	-22.88621233995825	8.02790961866159	1.52501171631861
C	-22.79193524946307	-9.06660350131137	2.93802626604395
C	-19.20139381920890	-13.81570776383435	-2.56715587695058
C	18.65421472921789	-15.14456615190769	2.70274805806858
C	19.40311609013728	-14.20258595772821	3.40429834719144
N	22.80196227747390	-7.86392316708084	-1.03435618379274
N	16.33246762237297	17.74518074483467	-1.52117418679806
C	-9.13953814935735	22.53891864736166	2.04151029352053
C	-8.54313014445503	22.53825466299553	0.73309818687244
C	9.44143373850771	25.11212774176499	-1.65742442732065
C	8.16286670451731	24.68889391209470	-1.52467770867579
C	8.22292948424969	23.30474410722865	-1.15775184303314
C	7.10895420776195	22.53642852632883	-0.87023412887065
N	9.52573970009833	22.87834717953783	-1.07844146040244
C	10.28057843594093	23.98763651847184	-1.36581375712850
C	11.65804363922586	24.05354896116711	-1.32121941786982
C	12.46979364176924	22.98021011141064	-1.01650464248841
N	12.04308309210403	21.69534740191807	-0.79371933375405
C	13.19126492541852	20.96996817281171	-0.59032494736782
C	14.34530849529781	21.81783833194459	-0.65126863826191
C	13.89730814782798	23.06541616160040	-0.92389455965908
C	13.25298881881012	19.59885572547464	-0.41775786910504
C	12.12530410300960	18.78689952662462	-0.39873469605699
N	10.82029619142913	19.20943059615148	-0.43549133178122
C	10.06899076917797	18.06167677585934	-0.34228415827136
C	10.91603471744736	16.90779202170882	-0.30759298208690
C	12.19175909812480	17.35846709308830	-0.32849834276334
C	8.68540808349479	18.00453220123605	-0.24463188062297
C	7.88704755191755	19.13997473812374	-0.28563345896832
N	8.32152356632463	20.42133840834801	-0.52603771714085
C	7.18320554706432	21.18860651363996	-0.54085484999798
C	6.03115286447778	20.38728413588040	-0.25667427277711
C	6.46816942058322	19.11670491928932	-0.09618806943538
C	24.82248481947552	-0.92435417267540	0.78544719348093
C	26.63378887360198	4.46015256589963	0.58094375654609
C	25.60978026749846	5.34472831968280	0.57007959021446
C	24.40220176310470	4.57909012941089	0.67269258119856
N	24.68131210230442	3.23578249163952	0.72720269527641
C	26.05056829999601	3.15549732864365	0.68357609992713
C	26.79032021529568	1.99305672357590	0.75453356452949
C	26.23640801804529	0.73110974348451	0.81636959754798
N	24.89438544585130	0.44682952952616	0.78697943703039
C	26.13267349492362	-1.50265318411309	0.83866850600669
C	27.01125711628034	-0.47362595187692	0.86128245455507
C	23.65836863233508	-1.66680828004017	0.70514027167251
C	22.39371180223136	-1.09298854384062	0.67955488101764
C	21.18927284621518	-1.86152333080927	0.58576134294644
C	20.16026500847653	-0.98554952039316	0.65189120757711
C	20.73520725622295	0.31827084506413	0.79206149359998
N	22.10761920656153	0.24561909321580	0.78198321815457
N	21.90212040397580	3.01950727051476	0.89600633303610
C	20.55964148465536	2.73902838156189	0.99179433379575
C	19.80275691576253	3.94517354460211	1.13821702756313
C	20.68543783121689	4.96927014209357	1.08856789038125
C	21.98561899007218	4.38929132461588	0.93398300084875
C	23.14397247451708	5.14428767709487	0.78806071989666
C	19.98363165661437	1.47804699227902	0.92079660385682
C	16.15281717570497	-18.79012191292724	-0.58925255795580
C	15.73201007025599	-20.91458961055361	-0.81942405932590
N	15.12362214745252	-19.69759244278285	-0.64116951257697
C	13.05943435373070	-23.55657327517533	-0.84675065379691
C	13.71978197057461	-22.28621420420821	-0.79136155678197
N	12.80621101454048	-21.26507090244098	-0.71484295115956
C	11.58001044296993	-21.88185901427400	-0.75139354842268
C	11.72984747655779	-23.30558182397454	-0.82427076162030
C	10.35689270595345	-21.23525341423841	-0.79177394248666
C	10.22951073940573	-19.85255685241980	-0.72629627976114
C	8.96620037680056	-19.17920306657270	-0.76637677349538
C	9.21897255788312	-17.86128208568881	-0.59424088834424
C	10.63549818569865	-17.72900533144894	-0.43583263123272
N	11.25083143530549	-18.95326940028813	-0.54535481455264
N	13.55717569438827	-17.41991190832928	-0.25345148990768
C	17.41594591657737	-19.44432892235975	-0.76595186629620
C	17.15416638476853	-20.76439109818224	-0.90819633057225
C	15.08911879707063	-22.13448118915460	-0.86370738809132
C	16.01924980764118	-17.43926907680679	-0.31951526642275
C	14.78753371041142	-16.82424264528015	-0.12729085637386
C	14.64482993998375	-15.43021149217637	0.16797001474245
C	13.31732943932029	-15.17225505406022	0.20898529746934
C	12.64751988400718	-16.40460302284878	-0.07708210490363
C	-7.13883707956694	-22.59186054111875	-0.29981375650486
C	-6.46159108840780	-19.12137972197509	0.16187257050693
C	-7.89227261951139	-19.16105259674737	0.12150479534681
N	-8.33477751352752	-20.45437363246137	-0.02741938228349
C	-7.19669300064437	-21.21245759019381	-0.13710933957215
C	-6.03000376984503	-20.39079386462560	-0.02121774882870
C	-8.25851282606776	-23.40257473899402	-0.24568535670715
C	-8.21026120678578	-24.83394649971684	-0.30459408264964
C	-9.47465151430283	-25.27415690416554	-0.10631659386487
C	-10.29778991764817	-24.11091157688014	0.04443770398539
N	-9.54626245207075	-22.96721596802713	-0.05049693621703
N	-12.05477956170401	-21.74612547233932	0.12706327080453
C	-12.47578006419513	-23.04486068828959	0.26630417188982
C	-11.66325336919894	-24.15976864170203	0.23762586994216
C	-13.90420666452427	-23.10853662922440	0.35779908501556
C	-14.35887174918175	-21.83895878290406	0.24309034124617
C	-13.20583788802910	-20.99804718055951	0.10802502039929
C	-13.26683287049803	-19.62240620587508	-0.03752920806397
C	-12.13419172739825	-18.81699852026216	-0.08247686127868
N	-10.83321928781556	-19.24348319537420	0.01832165578977
C	-10.08029659803194	-18.09494483759376	0.06723824135073
C	-10.91895026514944	-16.93936325602276	-0.03342582729151
C	-12.19215081836662	-17.38690226256043	-0.13150323843919
C	-14.69094638700270	15.40670621077643	0.24119704253488
C	-13.36696538327291	15.14466663920623	0.33810335814027
C	-12.68691590339521	16.39841031010789	0.21265793966205
N	-13.59049544089676	17.42769883193625	0.09207362979503
C	-14.82278801592643	16.82448242209593	0.09144171330673

C	-16.05272008693230	17.46509208665368	-0.00063791363186
C	-16.18657765655615	18.84183879069557	0.00598761274284
C	-17.44978963236068	19.51908929313781	0.01290507218265
C	-17.18168707380087	20.84088598249206	0.12512162040096
C	-15.75502446094871	20.97100707158972	0.16033833394360
N	-15.15175154168697	19.74149546639645	0.08026549171541
C	-26.64943431930029	-4.44948860753394	0.86692612001508
C	-25.64777461821596	-5.33250930666915	0.64607831680502
C	-24.43146801231690	-4.57495848982005	0.60940701273109
C	-23.17397887334345	-5.14247305240729	0.50633099025339
C	-22.00153615628534	-4.39966050541314	0.58523256177121
C	-20.70260423712082	-4.99742045638201	0.65775623683659
C	-19.81171319441845	-3.98552396314412	0.77443206316238
C	-20.56231684194476	-2.76690058897276	0.73469765781027
C	17.86430184712484	0.68273459518117	1.99182102033622
C	18.50503983795533	1.36716877988896	0.95811478507822
C	17.72713744798684	1.94310274908464	-0.04759739570415
C	16.34712814421093	1.83694633981825	-0.01994902933486
C	15.70204209135774	1.15358225898432	1.01399484516173
C	16.48356889416533	0.57881873090122	2.01933382790196
C	11.26866861046500	-16.51952652177888	-0.18576952892942
C	-19.98412621685454	-1.50496369352820	0.72181459992667
C	-16.50110215643049	-0.61824008590708	1.87301717500237
C	-15.70544024591014	-1.16718511821199	0.86441259233369
C	-12.04428526520045	-2.00737250961683	0.55109163743466
C	-11.30528519344321	16.52420659490002	0.17005607132752
C	-9.57541348580702	15.14295369552257	1.33162116300721
C	-8.79302450065771	14.00455746515311	1.42851272022422
C	-8.87889998629831	12.99328751458534	0.46841021838703
C	-9.77235710013690	13.15880170862203	-0.59287344483202
C	-10.55603026919290	14.29605373061774	-0.69023708863880
C	-10.46978097492503	15.30316730790457	0.27217133240878
C	-8.69651989690308	-18.03046921782761	0.16590513285383
C	-8.31311694502827	-15.87081291599518	1.36630386690904
C	-8.05572972935892	-16.69748294446747	0.27151106225050
C	-7.18973473961233	-16.24121552464379	-0.72359815072896
C	-6.59367913683227	-14.99567348236030	-0.62303482856102
C	-6.84590936310189	-14.16757987260373	0.47353374711272
C	-7.71653286598300	-14.62508422521213	1.46565319464216
C	9.52356610395365	-15.18005952774558	1.00825802422870
C	10.43878073415815	-15.29882674363132	-0.03853791322734
C	10.55695848852343	-14.24701872436486	-0.94819284348057
C	9.77858361044314	-13.10969932133158	-0.81612868375209
C	8.86125685233782	-12.98677687036406	0.23071137137031
C	8.74831692378760	-14.04039651347810	1.14138151985979
C	-7.77432513509833	9.39262942541156	0.30823797240386
C	-6.44548214832214	9.46886320998545	0.73354926178915
C	-6.32628421903344	-10.49308058049405	1.14127705657804
C	-4.99957505807510	-10.30418244304072	0.74585252640269
C	-4.28708565752584	-11.40749712486308	0.26898435699377
C	-4.88361059815336	-12.65297217904504	0.17995318421652
C	-6.21028422149573	-12.84162553652255	0.57534424199137
C	-6.91945380139996	-11.74116565671073	1.06297142836624
C	-4.36846554114394	-8.97451432054773	0.81883999803570
C	4.24221158132904	11.40550145298994	0.08134865641523
C	13.60934218759524	-0.13833516759372	1.45318202782211
C	12.22960083357816	-0.24707558226249	1.46119549085120
C	14.23266221438021	1.04116774124470	1.03816233857091
C	13.42701021223336	2.11114462533994	0.64024414495966
C	12.04737334633733	2.00564924037882	0.65746304054134
C	11.42376365384862	0.82314538925344	1.06409563906750
C	-2.43921583976081	-7.58541521620899	1.26529593281358
C	-3.15473683530051	-6.43575061358188	0.92213146532112
C	-4.48940220864706	-6.57568401915808	0.53392522341140
C	-5.08752382398406	-7.82284685191355	0.48868332111826
C	-3.03414282402007	-8.83377435222677	1.20892240359962
C	-1.62997113354488	-4.76581816279685	1.98315965566607
C	-1.02394502421610	-3.52128740861950	2.00869599644825
C	-1.28105259565060	-2.58456648215697	1.00711585524326
C	-2.16430673939577	-2.92497033571013	-0.01820369617998
C	-2.77344044577982	-4.16750224534032	-0.04103516170129
C	-2.51427330735738	-5.10898351638394	0.95787848835528
C	4.91160531499093	0.96609026227357	2.06814395980114
C	5.67665481446152	0.38676320768384	1.05302656993342
C	5.01605116028841	-0.29412316120904	0.02766943357243
C	3.63510742592147	-0.39018939281269	0.01582980476702
C	3.53101409783905	0.86735994189538	2.05841214296072
C	2.87365154927731	0.18885940814067	1.03151644249286
C	-9.16261144685478	-1.79070014737358	1.39979843723507
C	-7.78310024817815	-1.68175387525751	1.41924671371867
C	-7.15370625231550	-0.49359908155564	1.04011574024575
C	-7.95146815714227	0.58529580018637	0.65135307123138
C	-9.33136410281629	0.47976582541458	0.64256779495522
C	-9.96108925112821	-0.71180360871186	1.01137613746730
C	-3.54408551940885	-0.86426963531030	2.06060279545555
C	-2.88122694315341	-0.18695607320852	1.03651875130851
C	-3.63724056163624	0.39221972740825	0.01674578095956
C	-5.01812013561755	0.29687996152206	0.02149067026994
C	-5.68413818251960	-0.38398149459542	1.04333120819184
C	-4.92487105164375	-0.96291735812286	2.06287841037529
C	6.70016861470508	-11.86316287641604	0.78381742822708
C	8.03039713242541	-11.77697767888275	0.36494907941183
C	8.54991765589776	-10.51400829482956	0.06917612488408
C	7.76685040358441	-9.37900999648095	0.18510735722811
C	6.43332882404117	-9.46600497741840	0.59378357040730
C	5.91488781076515	-10.72884315215797	0.89250979994012
C	5.59467929002162	-8.25876022324780	0.69424770427180
C	4.24795324670598	-8.29106155943686	0.32238102453170
C	6.12559844916128	-7.04975601925360	1.15179668907381
C	5.33960079463313	-5.91334681838073	1.23053586688287
C	3.99666696497529	-5.94336247655895	0.84649948945077
C	3.46409794601856	-7.15297637287717	0.39339777715291
C	3.16604323490985	-4.72764684986518	0.90425402715849
C	3.29495036215770	-3.81690570392769	1.95561835157404
C	2.23513210444731	-4.45550628435312	-0.10136389735495
C	1.46222731462020	-3.30835481006967	-0.05958683152922
C	2.52062798244289	-2.66986963242758	1.99872761850781
C	1.59536072734642	-2.39898447572064	0.99040930979201
C	0.77287583447195	-1.16472803356245	1.02010267579146
C	1.39359145258455	0.09017580126729	1.02348896483309
C	0.61689835222047	1.25540446243567	1.01776988111122
C	-0.78038673165849	1.16554819445850	1.02175972782861
C	-1.40107807566442	-0.08946576363079	1.03033568035230
C	-0.62444058181917	-1.25463511448270	1.02382381070194
C	-1.60433809908717	2.39902251916621	1.00303960824443
C	-1.48011281783154	3.31723118288747	-0.04028996579922
C	-2.25382624045731	4.46450462390890	-0.06580743892997
C	-3.17745835193115	4.72655802831398	0.94897437089337
C	-3.29870808416600	3.80633873179940	1.99283677314219
C	-2.52256785104582	2.66021146499041	2.02045044842769
C	-4.00884188172511	5.94264219859162	0.91086683332632
C	-3.47621506188020	7.16067044920748	0.48136833555284
C	-4.25999341183460	8.30021815849945	0.43446147122304
C	-5.35185485353616	5.90404398654388	1.29357793372870
C	-6.13823523959495	7.04149185846621	1.23695626346082
C	-5.60654496594016	8.25961022364676	0.80559873307085
C	7.14626375361946	0.49515052554293	1.06161247125520
C	7.94704950860985	-0.58242457918254	0.67532678488093
C	9.32715416187476	-0.47915697936026	0.68377093516919
C	9.95469335570210	0.70853064774292	1.06874727649003
C	9.15305271298999	1.78673475660308	1.45277620124133
C	7.77328998665247	1.68030647731012	1.45407254753230
C	1.27392045292919	2.58483844492816	0.98498511632068
C	2.14656607959172	2.91379604717983	-0.05315866097969
C	1.02577039826273	3.53323147862454	1.97772405152953
C	1.62860338981909	4.77877132954217	1.92987897294932
C	2.75240895142639	4.15721018480923	-0.09831782027066
C	2.50057016687538	5.11117850966056	0.89058395564283
C	3.13537042374301	6.43967507898877	0.82814687035965
C	4.46457001056017	6.57847392755710	0.42120727031662
C	5.05694379139810	7.82698242965453	0.34831487511874
C	4.33747932082333	8.98116867731739	0.66885038445167
C	3.00872664161545	8.84161609534018	1.07787562381871
C	2.41957632765566	7.59198405399319	1.16173212048693
C	4.96345795333658	10.31125899940838	0.56590924036073
C	6.29546534100259	10.50883959919658	0.93872288753534
C	6.88559707235107	11.75591731481274	0.82958181027919
C	6.16789234971672	12.84704684822976	0.33327934821472
C	4.83548647640324	12.65008281501948	-0.03806305046535
C	-5.93210787053833	10.72159742404578	1.07923711783811
C	-6.71927592589758	11.85734375119809	1.00234821899184
C	-8.04476003990772	11.78218521315645	0.56714207218960
C	-8.55843084323411	10.52957900597515	0.22180721710353
C	6.80375613448465	14.16959179010230	0.19563154208333
C	6.53229770297038	14.97940442197221	-0.91006410629225
C	7.13666346799480	16.21723961997587	-1.04815901830462
C	8.03306905530155	16.68288384534356	-0.08465205291463
C	8.30215739678409	15.87868728251309	1.02397294194168
C	7.69640956600646	14.64102459478326	1.16137028105321
C	-11.42972899688821	-0.82947546272749	0.98383204271940
C	-12.24341277924822	0.23389985967887	1.38308066545978
C	-13.62265536675028	0.12264352036108	1.35271426636982

C	-14.23677331687038	-1.05196658431417	0.91121016100490
C	-13.42333052179838	-2.11479396213077	0.51029500262203
C	-16.33513137227486	-1.82655637124706	-0.19413749989064
C	-17.71445844197655	-1.93648745637266	-0.24175255691116
C	-18.50644437060620	-1.39093745516922	0.76985088759852
C	-17.88074779759054	-0.72740930565483	1.82643018165278
C	-20.73488080931446	-0.34151204956814	0.61460503412637
C	-20.15729369420452	0.95732956067036	0.44626591385662
C	-21.18435772347234	1.83258655434505	0.34592421256016
C	-22.39143697550600	1.07108242048941	0.46422763403625
C	-23.65323578521770	1.65204897826061	0.52618969750473
C	-24.80731390494809	0.92043038701671	0.74993947059885
C	-26.10542229079161	1.50588508972752	0.91412303814771
C	-26.97011099385737	0.48530117757569	1.12032744723362
C	-26.20442366630415	-0.72387441467201	1.05339109193778
C	-26.76280427786876	-1.98436638984562	1.11295955345602
C	-26.04744915892651	-3.15082263115764	0.93421614215207
N	-24.68824559381589	-3.23540227045656	0.76912210654871
N	-24.87743396526614	-0.44781539355888	0.83870251927385
N	-21.90886160764171	-3.03257970669047	0.65156485441526
N	-22.10681435987684	-0.26397269522947	0.60920424548128
C	-10.67194261359971	17.74706359763648	-0.00783385862208
N	-11.28807536604623	18.97377188823894	-0.07119713482473
C	-10.27240771111629	19.87643614779541	-0.26645788193313
C	-9.01150271307931	19.20353803904326	-0.35716614003096
C	-9.25891591619645	17.88363821766222	-0.19155908703669
N	-12.82839541965236	21.29599863060399	0.02622430765748
C	-13.72887949328640	22.32153233644523	0.17001412487747
C	-15.09972665985751	22.18248688199069	0.24302000542728
C	-13.06165183886179	23.58939997244263	0.15487830193304
C	-11.60642937965781	21.90951491948330	-0.09777697088358
C	-11.74495585873335	23.33447571483154	-0.02652138061203
C	-10.39680817210567	21.26108347597076	-0.27948960980851
C	-9.13154067782645	22.03495108488201	-0.43286258313371
C	-8.58290201978463	22.08879585224834	-1.71990516549205
C	-9.24414943071104	21.66162519596079	-2.92264505847177
C	-8.38444455427816	21.88193025509117	-3.95043339168217
C	-7.17923800333785	22.42008212411946	-3.38393409689148
N	-7.34016806330822	22.55389465109845	-2.03491290037335
C	-8.23840260197135	23.09578914669677	2.89074394739515
C	-7.06889400507323	23.40990680430609	2.11840133439054
N	-7.29304564146738	23.07994302032579	0.81301295388351
C	-5.86696969171316	23.88285203649006	2.64020919329342
C	-4.66294491681579	24.02998864428280	1.96212327437892
N	-4.48526196774819	23.80250016224061	0.62020061613933
C	-3.16317704237430	23.94209578440695	0.35703167315632
C	-2.46020375527433	24.27005375789754	1.57292001751407
C	-3.40182117210685	24.34433766996412	2.56664024846066
N	-4.54290047563795	23.29297825360993	-2.22189040659604
C	-4.77553841784070	23.04261878840634	-3.55139720472531
C	-6.00442706233037	22.67645797497071	-4.08717746484999
C	-3.54251273980761	23.12392510293030	-4.27784829829838
C	-2.56182022515596	23.39396087224357	-3.35855336757118
C	-3.21312262006828	23.50876622164035	-2.07688725291353
C	-2.52184580224896	23.79141819556602	-0.88525972339110
C	-1.06812173721297	23.89812638708194	-0.93421690358944
C	-1.03451609526098	24.37192132953046	1.52561583120987
C	-0.37405772614755	24.14453600840975	0.26381231255226
C	-0.42453202097221	23.71580918029318	-2.17117312856066
C	-1.13660639285423	23.50147249654204	-3.40698567032195
C	-0.04929845130445	24.58133256101493	2.45579316216205
C	1.20098250310241	24.44798404343670	1.67742149964440
N	0.96874342570002	24.19592361800145	0.43812139986867
C	-0.19118343448947	23.37936470912515	-4.39233879954887
C	1.08658706601861	23.48552200473196	-3.75139690907562
N	0.90980792392775	23.70012331029342	-2.40714522192372
N	3.85039924441316	23.87475465682917	0.43866663428048
N	3.79537580521997	23.39168242052136	-2.41815444117359
C	2.31692682496874	23.30705860776721	-4.37243165127211
C	3.56239078953483	23.22948691748833	-3.75318763696692
C	4.78990713577401	22.86518946953181	-4.40436744034332
C	5.74063523517389	22.77154967621025	-3.43950511519179
C	5.11093693047350	23.10905903238925	-2.19199218714254
C	5.73799728939358	23.12204505512399	-0.94131171830310
C	5.15688370661710	23.52191532160505	0.26688954530513
C	5.84110034739677	23.62168376879334	1.52696423283871
C	4.93088178020319	24.04258772104868	2.44257962647700
C	3.67483270062664	24.17591068346435	1.75825562395622
C	2.45605227551463	24.47394683054560	2.36369505829707
C	14.56510460622773	18.90328751239196	-0.27284835693247
C	14.93904592578942	18.54296187368152	1.02673363397044

C	14.28411719225958	18.97735958589660	2.23033285926761
C	14.95281300421648	18.41916884280216	3.27200915898089
C	16.00466848854165	17.61480105197672	2.71527340042359
N	15.98234959129174	17.72818580465797	1.35519677673769
C	15.24619881581080	18.56788601360820	-1.44807715987414
C	14.89658985913994	19.00387041697686	-2.77258275463793
C	15.78856558208708	18.43947501958383	-3.62667661066380
C	16.67296786653491	17.62763087929582	-2.83769473605931
C	17.65484474718185	16.78084526887318	-3.34724135651879
C	16.85218096307310	16.78267769335264	3.44306464183104
C	17.76504917393819	15.86927750425701	2.92973213828413
N	18.03446055613421	15.69507381236791	1.59498278759140
C	18.88326038802717	14.64448777049556	1.48672305737327
C	19.17511050827362	14.12138359462206	2.79871071734257
C	18.49239396164145	14.90088643658990	3.69641480865791
N	18.36836489655593	15.68942927662671	-1.27433630607922
C	18.41186332162743	15.85746508174464	-2.63595304723827
C	19.16231874107325	14.63401388001808	-0.97042714742808
C	19.73760546630854	14.09777299915462	-2.17942180475963
C	19.28350102877445	14.87632319744390	-3.21262014688862
C	19.41498509333308	14.09823661213037	0.30493189390470
C	20.23844523968507	12.89860815726976	0.40378075846219
C	20.73849116322691	12.33003368725287	-0.78110048932944
C	20.54093726846343	12.91836704796388	-2.08302651336886
C	21.16796397330997	12.09942858549548	-2.98635138287903
C	21.71328881264112	11.00432402805381	-2.23910783921125
N	21.44628907716994	11.18097460209185	-0.90434702312196
C	20.46689613333260	12.34593284818076	1.67663841470204
C	19.98762061791323	12.94870659669287	2.89601599643292
C	20.40887936346530	12.14746798898898	3.92572214710265
C	21.10918417240299	11.04766246286917	3.33035601183115
N	21.13565324932277	11.20378502963953	1.96678473546029
C	21.60748936332737	9.94461895718946	4.01322373147149
C	22.13027344448939	8.78167734529537	3.45236468537252
N	22.30175263130881	8.55436413273195	2.11717840031523
N	22.62088273473203	8.52804157861585	-0.76129331277426
C	22.71543649907140	8.72909176065658	-2.10802744928826
C	22.33212019123068	9.88551903694611	-2.78378797529284
C	23.17951760795468	7.52165850439349	-2.73254145855501
C	23.31782013213980	6.59293614548607	-1.75184128402774
C	22.97279089930553	7.23327706561700	-0.51155633641472
C	22.96983796524366	6.62455047900534	0.74851625451382
C	22.70663583357220	7.26076150587548	1.96797930457506
C	22.47954294731756	7.59392291061823	4.18044736736976
C	22.81866044939606	6.65033570035421	3.26464316682742
C	23.70525213294347	-3.15797634703849	0.64833342077211
C	23.73869284004801	-3.74197099582001	-0.62234892388294
C	23.94135615201169	-3.03823534771313	-1.85918644233849
C	23.89383784142102	-3.96027343853630	-2.85488078502578
C	23.63150196016875	-5.23199113339407	-2.24019542504486
N	23.56570700879429	-5.06926623138382	-0.88668489901251
N	23.40067243139082	-5.18237725825586	2.00473036988785
C	23.59193467233525	-3.83885722910288	1.86507297794470
C	23.64717719202594	-3.23332180668110	3.16755976014384
C	23.49514191365523	-4.22988248863971	4.07725942871421
C	23.31499857299636	-5.44982180824431	3.34033413178212
C	23.00865753684846	-6.68890804849705	3.89840643097338
C	22.66268873961970	-7.84819872125571	3.21458513827138
N	22.64422753160528	-7.97765364318155	1.84805616961450
C	23.38823741999602	-6.42100518284523	-2.92420477174734
C	22.96834485622630	-7.62721329469727	-2.37622697646419
C	22.28256080394923	-9.10870083956241	-0.90356748261841
C	22.10492749765510	-9.69806442307826	-2.20791066099986
C	22.55309295781909	-8.77920986785553	-3.12148947611874
C	21.94148874562100	-9.76745372969616	0.29111196021099
C	22.15046012533568	-9.20718232943125	1.56396118867576
C	22.17871070579742	-9.05534139764925	3.81731843130507
C	21.83847200084652	-9.89812988884694	2.79090883068631
C	21.22013973749086	-11.18474995678126	2.70635693643634
C	20.72739321753392	-12.08195327890715	3.61845624434804
C	20.12012374092661	-13.14048524076908	2.86660704390859
N	20.27530587286941	-12.89835682519543	1.52434061403678
C	20.93803049960732	-11.72324863029277	1.39853155078415
C	21.30862507642401	-11.07865351364519	0.20483896030936
C	21.06715686352434	-11.62344765172036	-1.06883753054933
C	21.48293159408844	-10.98315053058011	-2.29247859953232
C	21.08873935473914	-11.80039589801001	-3.32033518017776
C	20.40864494928313	-12.91240884981313	-2.72380474545551
N	20.42367004425353	-12.78067649854639	-1.35757404214443
N	18.55261618983599	-15.22240446195387	1.34361996002469
C	17.78815329249221	-16.12472278087909	3.29621406824465

C	17.14342597629456	-16.75418758607515	2.28028443634148
C	17.63018767289156	-16.18411220547079	1.05372201144440
C	17.21893881793105	-16.55771396496491	-0.23150971098292
C	17.75744682880985	-16.08974930613309	-1.43513119076786
N	18.70624237640614	-15.11519304345481	-1.54703031498280
C	19.75049833795599	-13.92096405378563	-3.41741222971161
C	18.93601738355782	-14.91383056177469	-2.87729504433034
C	18.12505980065017	-15.82344180099953	-3.63784015179181
C	17.38288791825553	-16.53397404611438	-2.75022594624384
C	9.08805023985870	-22.01196869880349	-0.89474684898310
C	8.46219625287779	-22.02503181453199	-2.14730594892535
C	9.04670898058042	-21.55288972665365	-3.37282556686579
C	8.12497151921713	-21.73962567582170	-4.35236311982680
C	6.95815137642989	-22.30175987526215	-3.73153017213322
N	7.20332406272998	-22.48394878241226	-2.40098232537978
C	8.57624611426536	-22.56268458800274	0.28530501310424
C	9.25532350102331	-22.61137920188878	1.55165178754381
C	8.41313088748980	-23.20873869067242	2.43335751358731
C	7.19812799657727	-23.50088354030825	1.72495068815622
N	7.33666684847961	-23.11698163679145	0.42249798652938
C	5.74082898230083	-22.53310018276630	-4.36782507169137
C	4.54856060330401	-22.92166155862040	-3.76890775472563
N	4.40217599129267	-23.22824819404953	-2.43886806052238
C	3.08450613032210	-23.45340008068340	-2.21761188627493
C	2.35167679505891	-23.28368603063518	-3.44811236909890
C	3.27100751137510	-22.97333816517654	-4.41670354605942
N	4.52718331795854	-23.85394693064162	0.37791223452995
C	4.79129607918056	-24.13718460582411	1.69479253927098
C	3.57323595237520	-24.49070554370241	2.36291857892987
C	2.56993255332781	-24.38309007096996	1.43468113210022
C	3.19205617761505	-23.99449094823292	0.19284744901100
C	6.03476117571898	-24.00609097044132	2.30142918932362
C	2.47198297062002	-23.79269975683298	-0.99798344236060
C	1.01850059588364	-23.90608270707451	-0.95854740358380
C	0.29594756169114	-23.66673757553528	-2.14108194365688
C	0.92622547925088	-23.38960613987418	-3.40838059283645
C	-0.08121330975494	-23.21630878829692	-4.32194744940664
C	-1.31444118266442	-23.35437766619849	-3.60453236315717
N	-1.05073836921885	-23.64041473574195	-2.28805843102081
C	0.40380565269552	-24.21587752601926	0.26766017314026
C	1.14484552283963	-24.49891732635876	1.47219276789861
C	0.22223868033400	-24.76814913139463	2.45004198218593
C	-1.07029710739715	-24.61600406055288	1.84909682706843
N	-0.92495935133152	-24.29126343674541	0.52337764091852
N	-3.80875245031282	-24.00895659501835	0.72399846070582
C	-3.54364238308932	-24.38112041824350	2.01018109525036
C	-2.28522369847528	-24.69371777428094	2.51958043985720
C	-4.75212456816885	-24.29848229053459	2.78240245664635
N	-3.92563832705909	-23.33000502691589	-2.09034971168422
C	-2.58177864328490	-23.13715529969804	-4.13157437133182
C	-3.78222823593839	-23.09065236677662	-3.42652092716720
C	-5.22205055061851	-23.06381758171906	-1.76092020121354
C	-5.12598020227172	-23.65817543222973	0.65931856129734
C	-5.77639400195877	-23.17231447116615	-0.48036856996391
C	-5.72330213288613	-23.83360370720539	1.95513290145305
C	-5.04943048903196	-22.69034259890218	-3.97173863680754
C	-5.93339997253316	-22.65597582383516	-2.94158689234288
C	-14.57932080589610	-18.91713614515763	-0.08719703756638
C	-15.24176707338476	-18.70422699755689	1.12726704812849
C	-14.86601480450758	-19.26207317201319	2.39822879915202
C	-15.75346190845084	-18.79913056987587	3.31556623018593
C	-16.65965961215076	-17.92616868450049	2.62302162098362
N	-16.33562583694080	-17.90583777713915	1.29722829499357
C	-17.64759660449832	-17.15278519470827	3.22858395467616
C	-18.42142636702432	-16.16694208298879	2.62822451695014
N	-18.38508850639942	-15.84513018832338	1.29435903692939
C	-19.30006507426828	-15.26644171026957	3.31523849253698
C	-19.76404794121476	-14.37889238405113	2.37888972997696
C	-19.18720185587450	-14.76755235814523	1.11543092432480
N	-16.01826356657971	-17.57568224919703	-1.56254574406675
C	-16.06021281859213	-17.32081819708237	-2.90310769089585
C	-15.02594923710496	-18.07241042981732	-3.55753895040517
C	-14.34693390470146	-18.74147231620883	-2.59046019541047
C	-14.97680281401054	-18.42668887294128	-1.33722555815253
C	-16.90642297748738	-16.40618928153651	-3.52572976847834
C	-17.80564372157400	-15.54716725158721	-2.90548395652645
N	-18.06842899832283	-15.52603664457999	-1.55831312340743
C	-18.91085719299975	-14.49053043797827	-1.32609706776623
C	-19.44182911644099	-14.08648151191289	-0.08813007418125
C	-18.52742588757128	-14.49086833998626	-3.51185234137141
C	-20.26603456391092	-12.88407888036218	-0.04493205798482
C	-20.57672788636012	-13.20272076287402	2.42034347764469
C	-20.77207973628131	-12.46308770647817	1.19769720177664
N	-21.48844155129700	-11.34171632179020	1.45460989489828
C	-21.76640911012800	-11.33088694274951	2.79876475931670
C	-21.21610000255336	-12.50403350118886	3.41175191482998
C	-20.48827770683022	-12.18140144750946	-1.24266718388863
C	-20.00496257051091	-12.63363277855054	-2.52409194125244
C	-20.41171310414250	-11.70761979477337	-3.44965976640063
C	-21.10791574783751	-10.68356741934202	-2.72764425167383
N	-21.14835946125374	-11.00788718909837	-1.39436284329075
N	-22.67576040869006	-8.69697968071149	1.62924485746128
C	-22.40363545874090	-10.29415307551993	3.47012039565866
C	-23.28100018166294	-7.95148256997898	3.70018201763488
C	-23.41166708606995	-6.90785849018122	2.84143757142143
C	-23.03480313483791	-7.38357452157138	1.53821072027343
C	-21.58443057632789	-9.49658939120209	-3.27093546680260
C	-22.10467775515137	-8.40886642401306	-2.57333035682102
N	-22.30744631177868	-8.35672208841754	-1.22449705579098
C	-22.70681051147162	-7.08949230879286	-0.91655369239901
C	-22.77684316531284	-6.31181005861207	-2.12343246146604
C	-22.42317881171434	-7.13103067612404	-3.14696664410177
C	-23.00487478724183	-6.61827712211923	0.36710777088541
C	-23.70075293428058	3.13745622531357	0.40788548003369
C	-23.79815603996441	3.87507661063153	1.59339839153024
C	-24.04199697102036	3.33268957817295	2.90260973284448
C	-24.04650803641305	4.37532531126577	3.77226154501453
C	-23.77102231849073	5.56143010009176	3.01058538571233
N	-23.64594669939197	5.22775435828925	1.69295070332508
N	-23.31909312535962	4.98072976686616	-1.17425842327306
C	-23.17404544887176	5.07978638851908	-2.52811560223953
C	-23.33305938377126	3.77848404385513	-3.11484088039902
C	-23.53274037785723	2.90362129937880	-2.09569223666710
C	-23.52518702009609	3.66590258129168	-0.87697077570623
C	-22.83257280605932	6.23853635538947	-3.22162823115003
C	-22.51452542499338	7.47489231662890	-2.67254978076904
N	-22.57116412034102	7.77697678687082	-1.33467715471921
C	-23.56921056746871	6.83052507512698	3.54862724057123
C	-23.12684744688653	7.96251344873541	2.87479245198779
C	-22.36181495356615	9.25032105868385	1.26556732206524
C	-22.26018190089129	10.00306564826635	2.49161400143715
C	-22.75723005701297	9.20402219688194	3.48871325212795
C	-22.08741838766250	9.03341947082699	-1.18225252229761
C	-21.07973135778486	10.84930027761454	-2.51176825694721
C	-20.87523286424407	11.55341728342501	-1.26977115236219
N	-20.19679490838578	12.70241654871001	-1.50528618184367
C	-19.95366736325773	12.76636924498368	-2.85485142828393
C	-20.52222491595617	11.61932992053241	-3.49962859791910
C	-21.32071546176693	11.06746708319443	-0.02754333627566
C	-21.64640752129732	11.29406452877233	2.44600515956651
C	-21.15625403607875	11.77377360665109	1.17726063931660
N	-20.53272031496709	12.96427454650639	1.35186191620253
C	-21.31869629335023	12.24208298875635	3.38071817535120
C	-20.60451861795279	13.27357657121906	2.68727564893091
N	-18.47622787839708	15.03511887290209	-1.51986213533335
N	-18.82956687535636	15.31533432298358	1.34262609413140
C	-19.15124892723015	15.29465532735101	2.66892971479533
C	-18.39850096805590	16.31008575158470	3.35151726642047
C	-17.59792904026241	16.90177630738018	2.42809379897515
C	-17.87675728518793	16.27573416536772	1.16442099600689
C	-17.25111749868256	16.57710371992633	-0.05028088996297
C	-18.47837578090273	14.77025291928052	-2.85882971281651
C	-19.18909093722801	13.74492602798131	-3.47859551319099
C	-19.99674634367967	14.37279686616327	3.28218959728006
C	-17.55690221159293	15.65544188960745	-3.51525817761331
C	-16.98047214556294	16.41754933632316	-2.55050467335103
H	-21.83489496421418	8.62747854715980	-4.46270801293950
H	19.36570285548400	-14.26520762599295	4.48759857650118
H	-10.12482793485785	22.16601301107752	2.27167821623433
H	9.80569048223503	26.09300063309611	-1.91889420290221
H	7.24746801153720	25.24429628461256	-1.65310043696596
H	12.12959625553808	25.00809295011394	-1.52377520612607
H	15.35843302936691	21.47631507114830	-0.51042833385748
H	14.46339979505262	23.97421425567537	-1.05347810574732
H	10.56956442501629	15.88783966349206	-0.27536602262297
H	13.10433522577566	16.78438526396167	-0.31052072767223
H	5.02134653685775	20.75998018614879	-0.19390510596787
H	5.89161636944221	18.23678198882516	0.13800846761437
H	27.69380553459974	4.65163931434459	0.52774548205216
H	25.64454042345528	6.42084183815263	0.51073390955083
H	27.87091473015273	2.07567565393634	0.74787765747403
H	26.32830976174510	-2.56307367788864	0.85318779853257

H	28.08871827237391	-0.50437217652881	0.89720436291597
H	21.14962355157180	-2.93400562981640	0.48126567891076
H	19.10361219566454	-1.19177157679202	0.60277906678408
H	18.73338829240707	3.99175233762935	1.26338361600906
H	20.48381956169264	6.02627223436208	1.15237438755510
H	13.56780934160361	-24.50600695770096	-0.90389832457921
H	10.90797469267296	-24.00269487172784	-0.86052629269486
H	8.01297242920055	-19.66274770736304	-0.90677421087855
H	8.51715287005390	-17.04360240488231	-0.57729591791608
H	18.36807580482832	-18.93813098805928	-0.76717309480298
H	17.84533477302578	-21.57922775697986	-1.05470934500928
H	15.69585069037759	-23.02670935844128	-0.96557887702273
H	15.46252302603163	-14.74479474227716	0.32208930849051
H	12.82524586875122	-14.23588911523224	0.41518414968273
H	-5.87168659242530	-18.23205763401043	0.31172401159218
H	-5.01508208713394	-20.75333732548843	-0.05605202056175
H	-7.30993436396678	-25.40603086411794	-0.46261654332788
H	-9.84017671901088	-26.28826742380471	-0.07249417788824
H	-12.13074982998717	-25.13329265075195	0.32858046521663
H	-14.46768933972973	-24.02043316235812	0.47640575176094
H	-15.37605714354162	-21.48130301670854	0.25050953659119
H	-10.56712269159398	-15.92071830850170	-0.04148320112091
H	-13.09755558724961	-16.80823916030339	-0.21883897037735
H	-15.51493535329226	14.71254920511121	0.28510150297491
H	-12.88476359000251	14.19166071544373	0.48170395397920
H	-18.40556890582390	19.02329743266697	-0.04678412586494
H	-17.87073648484776	21.66927855552809	0.17145335792138
H	-27.70682488891628	-4.63724794048399	0.96615130952168
H	-25.70087240074507	-6.40341435023073	0.53125758077400
H	-20.51087366499916	-6.05842954651117	0.64268930150070
H	-18.74093785236152	-4.04908743578445	0.87806808252962
H	18.45441790921920	0.24421980735798	2.78897183126642
H	18.21205062943913	2.46169677090321	-0.86727487445881
H	15.76483173543648	2.27218286382407	-0.82467966156455
H	16.00620596873969	0.06152873330442	2.84438110068791
H	-16.03554231509717	-0.11786638250887	2.71506286416708
H	-11.43614270414566	-2.84349438976708	0.22320383666264
H	-9.50704946499899	15.91150517431078	2.09361932892911
H	-8.12158909326280	13.89150821727599	2.27263351361385
H	-9.83986131227128	12.39972302049076	-1.36447830669248
H	-11.22901033174394	14.41660656887302	-1.53195772999140
H	-8.97369289996834	-16.21725765369448	2.15345782238587
H	-6.99918731011928	-16.86135200470153	-1.59253914993076
H	-5.94117257256848	-14.65306805139162	-1.41865000756424
H	-7.91392558841022	-14.00891021461613	2.33601829871171
H	9.43305681978120	-15.98194347920917	1.73265713496429
H	11.25192273156836	-14.33218986912899	-1.77628138546518
H	9.86987058648223	-12.31624923027018	-1.54974021370307
H	8.06052337068629	-13.96102821680483	1.97609858229403
H	-8.19441499890928	8.43458627510115	0.02167606702038
H	-6.89698790605879	-9.65672482182772	1.53022086578392
H	-3.25948388871316	-11.28580492396443	-0.05609361490565
H	-4.30588558520113	-13.49450940101335	-0.18684162674220
H	-7.95445109682468	-11.85826940109259	1.36560278003411
H	3.21007286519562	11.27700743576886	-0.22629368636028
H	14.21129830491157	-0.98859916442016	1.75517840704429
H	11.77154928372418	-1.17126457526269	1.79664311851685
H	13.88473753117412	3.04386669218219	0.32883376552810
H	11.44626601644981	2.84757820424841	0.33149779463632
H	-1.39828322086877	-7.50405054660870	1.55942762537807
H	-5.07107761122308	-5.69633275299287	0.27887022078547
H	-6.12148694200108	-7.90555367099508	0.17159149257594
H	-2.45857662370449	-9.71014631780981	1.48671599673735
H	-1.42839914799908	-5.47251203381701	2.78074143581700
H	-0.34829358753296	-3.26846158247527	2.81819999642307
H	-2.36634083363470	-2.21231697341691	-0.81001330293619
H	-3.44101172936506	-4.41801783664694	-0.85817934869306
H	5.40234488480721	1.48247020433684	2.88585633123984
H	5.58683454244771	-0.73495599611116	-0.78228598176906
H	3.13917748130791	-0.91140805022657	-0.79535189860276
H	2.95377495091529	1.31440035467141	2.86011508071657
H	-9.62675411908235	-2.72182248633169	1.70646318498510
H	-7.18526837657080	-2.53767900683229	1.71361496403232
H	-7.48720324199684	1.52376628926183	0.36780624132139
H	-9.92748356286030	1.32848475659687	0.32509971547751
H	-2.97119748304620	-1.31100520708048	2.86557981128273
H	-3.13684869157479	0.91271624920900	-0.79215496671007
H	-5.58472597195554	0.73744587167060	-0.79153194243760
H	-5.42022410598319	-1.47937656028627	2.87776879743853
H	6.26721567293261	-12.83200542107076	1.00823225347480
H	9.58447725867149	-10.41565292421581	-0.24139938760589
H	8.19213397108726	-8.41322313755419	-0.06550627538658
H	4.88819943646642	-10.82463560943187	1.22889223762145
H	3.81266147175148	-9.21296468972695	-0.04778530146915
H	7.16312171034143	-7.00014604243466	1.46408144387394
H	5.78076752437062	-4.98424776712472	1.57522561498216
H	2.41954679415063	-7.20796351116153	0.10617754303380
H	3.99548510258683	-4.01592418886405	2.75923445749725
H	2.13045522611562	-5.13664480259645	-0.93871577482162
H	0.75420934052861	-3.10716564787130	-0.85579127469025
H	2.62889110373836	-1.97812597017669	2.82668840090573
H	-0.77818390115971	3.12355988078293	-0.84369793766976
H	-2.15559505620271	5.15394832727476	-0.89714834352405
H	-3.99401853833467	3.99769499334617	2.80282690428066
H	-2.62360385194977	1.96141181394665	2.84337861589040
H	-2.43172335468212	7.22074542749185	0.19487608561346
H	-3.82517217351872	9.23017748062726	0.08438450765565
H	-5.79225088564256	4.96793965162965	1.61981462588869
H	-7.17613694697285	6.98592902710910	1.54700345669672
H	7.48493526872082	-1.51854859671816	0.38071732434306
H	9.92538763737374	-1.32706935421826	0.36813534499547
H	9.61459144772042	2.71523471082927	1.77106666144215
H	7.17365970860552	2.53590536985697	1.74567191054256
H	2.34183363824563	2.19156462610862	-0.83796470244416
H	0.35897518076638	3.28941367197926	2.79726350657646
H	1.43354523681174	5.49528987204515	2.72028887387639
H	5.04661289360279	5.69742695204561	0.17297738051087
H	6.08643592649918	7.90842105951122	0.01670364567707
H	2.43302488479243	9.72013716493052	1.34851820817032
H	1.38264141214620	7.51137136086861	1.46989655806463
H	6.87321198223643	9.68017610773102	1.33366474389822
H	7.92500722480472	11.87913784160569	1.11409397598950
H	4.25091846874387	13.48428013169891	-0.41072798051510
H	-4.90869387855317	10.80719386973633	1.42816084248585
H	-6.29197685204193	12.81908078529485	1.26497657245972
H	-9.58937226700402	10.44084224688066	-0.10340225469584
H	5.85842750317422	14.62783958875085	-1.68364696422916
H	6.92912052569892	16.82452983402221	-1.92230085215477
H	8.97863472980178	16.23614213060150	1.79245954731857
H	7.90510791990716	14.04204995294167	2.04105463437040
H	-11.79209226228882	1.15414986998659	1.73792331555432
H	-14.23125681778828	0.96710182977643	1.65767619066032
H	-13.87487688795788	-3.04338834345251	0.17817153642460
H	-15.74107490003130	-2.23927044792437	-1.00216452605239
H	-18.18688298560845	-2.43297199989303	-1.08213690091060
H	-18.48269296510989	-0.30924131861482	2.62567345019135
H	-19.10001929430009	1.16036127508963	0.39674802989447
H	-21.13926866373071	2.90174924961636	0.21496835335537
H	-26.30491416656188	2.56491763436812	0.87806317397503
H	-28.03504375653401	0.52445306652683	1.28622662507429
H	-27.83359704356157	-2.06071608432139	1.26177518955631
H	-8.06258936496206	19.68956294655872	-0.51727204021051
H	-8.55692864114932	17.06599640662775	-0.20464891800146
H	-15.69898751501131	23.08130164280659	0.33012103426598
H	-13.55860559964679	24.54126159203495	0.25550878377861
H	-10.92505096406992	24.03049081947411	-0.10395770201991
H	-10.24320544592842	21.25734356706689	-2.96513699617391
H	-8.53683481607622	21.68461690676924	-5.00007170109662
H	-8.33870142596983	23.26234153390737	3.95192677202482
H	-5.85269669336132	24.09109655968071	3.70574120336037
H	-3.25294452141090	24.56255270750219	3.61234773170304
H	-6.03632913513486	22.50666684070503	-5.15910994878546
H	-3.43702489391397	22.96646289407433	-5.33963053307400
H	-0.15822413834686	24.77794622699288	3.51067552810998
H	-0.34365883179353	23.20580890139085	-5.44587098943996
H	2.30009128268667	23.14088910513980	-5.44528073592208
H	4.89553758471785	22.68498331693343	-5.46280715618097
H	6.77997936860618	22.50724822316434	-3.55383133200482
H	6.88710993123984	23.40586455041612	1.67762742103659
H	5.08218563405945	24.23093350961676	3.49403219925685
H	2.48380072627328	24.68110706818459	3.42914761617606
H	13.43002566924876	19.63524685885742	2.26316608318899
H	14.74866490638248	18.52204745012084	4.32635627365678
H	14.07703276122509	19.66412608460796	-3.00814014093483
H	15.83845660236105	18.54063007201599	-4.69962235159634
H	17.79374248036808	16.79616028429620	-4.42399699150845
H	16.73807423709981	16.80174143323673	4.52263546682795
H	18.46121170056801	14.81038938119758	4.77071575380317
H	19.49591650034923	14.77831777605696	-4.26551476255632
H	21.23021419690475	12.21098125058138	-4.05732645697401
H	20.23704497974856	12.27366544874577	4.98299913059329
H	21.51738327290072	9.96100160065044	5.09510990070068

H	22.46755722755276	9.88177435453028	-3.86109095781310
H	23.35231525183896	7.40285150373090	-3.79081879276818
H	23.63297341554324	5.56626833147515	-1.84981725259680
H	22.44847100849609	7.49852026402453	5.25460423876985
H	23.12956615232939	5.63274453017551	3.44109135525646
H	24.10800347719080	-1.97589313852883	-1.94279518449917
H	24.00545186120947	-3.80069701441614	-3.91601663144622
H	23.79467794271964	-2.18080218973574	3.35136277425364
H	23.48479059883747	-4.15334497468660	5.15334650652392
H	22.96950732666989	-6.73406477802810	4.98251965273994
H	23.46939395736572	-6.38092946857925	-4.00619388722116
H	22.57382305037910	-8.86240513886472	-4.19664454934635
H	22.08405175090622	-9.22365112608763	4.87845103964540
H	20.74744306888248	-12.01862266705444	4.69496704404915
H	21.22031021370175	-11.65046440758776	-4.38029575515151
H	17.68048280276867	-16.28980435383215	4.35689250981448
H	16.41081811926275	-17.54318057421169	2.34417054036117
H	19.82043382253846	-13.89078904956535	-4.50052541864887
H	18.11526663713817	-15.88668222775235	-4.71479842438710
H	16.65118024534896	-17.29845457032447	-2.95812944807581
H	10.04053510308101	-21.14370422740026	-3.46322138961824
H	8.21144072214858	-21.50452537860842	-5.40171199591628
H	10.25208244073565	-22.24126727398849	1.73196987995125
H	8.58207951266868	-23.41740430660284	3.47827168681595
H	5.70407949720966	-22.32150291920415	-5.43212630282308
H	3.09713695966668	-22.77073121257188	-5.46169378337567
H	3.49249381339411	-24.75573601735424	3.40529500928048
H	6.08945508771319	-24.25833200385434	3.35609635845625
H	0.00251033104409	-22.98796509977035	-5.37272490312179
H	0.39938657943524	-25.01774559440783	3.48427203379745
H	-2.24302352350042	-24.95863971437885	3.57170850903922
H	-4.83181043049382	-24.54538191836048	3.82959942673587
H	-2.63524404456586	-22.91149518067277	-5.19227029694465
H	-6.75624959491026	-23.63216277092609	2.19065321944800
H	-5.22535224269369	-22.45000267233566	-5.00865401145476
H	-6.97866177764537	-22.39134925625415	-2.96964152852564
H	-14.03295926547898	-19.92761863915997	2.55909621898438
H	-15.78576007775350	-19.00643144000435	4.37381893718636
H	-17.77609480711179	-17.28642286367885	4.29843317514735
H	-19.50962449054163	-15.29121577460758	4.37295852159176
H	-14.83986922383523	-18.06788309735709	-4.62019345611705
H	-13.50109716225516	-19.40073709300336	-2.70561941034258
H	-16.80272612192106	-16.30722905912964	-4.60198559755061
H	-18.49881062570068	-14.27669264212638	-4.60854493387512
H	-21.28410303458394	-12.74512277547891	4.46082792602283
H	-20.23207984184294	-11.70340111951394	-4.51314513754187
H	-22.55166416881003	-10.42560073693884	4.53770847749021
H	-23.47485795422931	-7.96852838510229	4.76134091774484
H	-23.74068947766301	-5.90448843595211	3.06057096624087
H	-21.47643413060081	-9.37526331394709	-4.34447041276123
H	-23.07215411486288	-5.27552274906603	-2.17139720089865
H	-22.36224144614896	-6.89550708752141	-4.19802461516844
H	-24.19612052155573	2.28775394830445	3.11979765283749
H	-24.19901212496454	4.35062943527563	4.83999220015496
H	-23.27900073016686	3.56868951266629	-4.17169774754508
H	-23.68432364883962	1.83731677228591	-2.15363475981162
H	-22.73956901889279	6.14673812266058	-4.29949602581868
H	-23.70468262137899	6.92623915903535	4.62167469031193
H	-22.83876503022246	9.42331942692547	4.54159514253611
H	-20.47471166015237	11.41589027951041	-4.55776186806374
H	-21.51634968456658	12.23034908451120	4.44095456145562
H	-18.46497745878619	16.52058200146223	4.40754971983179
H	-16.88554370642148	17.69746108270972	2.57807249864529
H	-19.07658214560266	13.66126550036283	-4.55526309989356
H	-20.13877454829975	14.48599760289368	4.35267046859019
H	-17.36822737797799	15.67057495889401	-4.57737470106061
H	-16.23339445140549	17.18678299372017	-2.66700350848352
H	3.41064809626528	4.39871221065267	-0.92567345175468
Ni	10.17761335781910	21.05121073686746	-0.70741118781695
Ni	23.39617716468802	1.73698778217119	0.79877208775792
Ni	13.18437638315878	-19.33387712788850	-0.53848554337983
Ni	-10.19241400144519	-21.10256772975697	0.01613015417373
Ni	-13.21450712729529	19.35972079510542	0.03102929072617
Ni	-23.39524771421947	-1.74474490198563	0.71642651567871
Zn	-5.92077787295276	23.20804664962686	-0.70955075393796
Zn	2.38243795133172	23.81659523486217	-0.99198777359386
Zn	17.19873169002542	16.73206504871173	0.04026053905780
Zn	21.89984287338702	9.87916482898730	0.60607950136828
Zn	23.12791011190336	-6.53150000612608	0.48377343322812
Zn	19.51190291744432	-14.01872787774301	-0.00956054631151
Zn	5.87250089695368	-23.19628758518575	-1.01474356206439
Zn	-2.42919827226146	-23.84378551412229	-0.78782422431193
Zn	-17.22168167270436	-16.73081504376990	-0.13544609188554
Zn	-21.92883225147725	-9.86264851896511	0.11270720306083
Zn	-23.13087832736130	6.51153788210435	0.17484691778356
Zn	-19.53104895704138	14.01881361917311	-0.08601321717700

7. References

[1] C. Villegas, M. Wolf, D. Joly, J. L. Delgado, D. M. Guldi, N. Martin, *Org. Lett.* **2015**, *17*, 5056–5059.

[2] F. C. Grozema, C. Houarner-Rassin, P. Prins, L. D. A. Siebbeles, H. L. Anderson, *J. Am. Chem. Soc.* **2007**, *129*, 13370–13371.

[3] H. Kai, S. Nara, T. Aida, *J. Am. Chem. Soc.* **2008**, *130*, 6725–6727.

[4] Q. Ouyang, Y. Z. Zhu, C.-H. Zhang, K.-Q. Yan, Y.-C. Li, J.-Y. Zheng, *Org. Lett.* **2009**, *11*, 5266–5269.

[5] H. Gotfredsen, J.-R. Deng, J. M. Van Raden, M. Righetto, J. Hergenhahn, M. Clarke, A. Bellamy-Carter, J. Hart, J. O'Shea, T. D. W. Claridge, F. Duarte, A. Saywell, L. M. Herz, H. L. Anderson, *Nat. Chem.* **2022**, *14*, 1436–1442.

[6] M. C. Biesinger, B.P. Payne, L. W. M. Lau, A. R. Gerson, R. St.C. Smart, *Surf. Interface Anal.* **2009**, *41*, 324.

[7] A. P. Grosvenor, M. C. Biesinger, R. St.C. Smart, N. S. McIntrye, *Surf. Sci.* **2006**. *600*, 1771.

[8] M. C. Biesinger, L. W. M. Lau, A. R. Gerson, R. St.C. Smart, *Appl. Surf. Sci.* **2010**, *257*, 887.

[9] A. Rienzo, L. C. Mayor, G. Magnano, C. J. Satterley, E. Ataman, J. Schnadt, K. Schulte, J. N. O'Shea, *J. Chem. Phys.* **2010**, *132*, 084703.

[10] S. Grimme, C. Bannwarth, P. Shushkov, *J. Chem. Theory Comput.* **2017**, *13*, 1989–2009.
